# CUE: A Chemical Uncertainty-Aware Embedding Framework for Multimodal Drug Selectivity Prediction

**DOI:** 10.1021/acs.jcim.6c01761

**Published:** 2026-09-17

**Authors:** Jin Hyuk Kim, Gyeong Hwan Kim, Hyeon Jun Park, Jonghwan Choi

**Affiliations:** Department of Computer Engineering, 26727 Hallym University, 1 Hallymdaehak-gil, Chuncheon-si, Gangwon-do 24252, Republic of Korea

## Abstract

Accurate prediction of drug selectivity is critical for prioritizing candidate compounds with reduced off-target effects in AI-based drug discovery. Although selectivity can be inferred indirectly from drug–target affinity (DTA) prediction, cumulative errors from affinity predictions across multiple targets can reduce reliability, motivating the development of methods that directly predict compound-level selectivity. We propose a Chemical Uncertainty-aware Embedding (CUE) framework that integrates molecular fingerprints and 2D molecular structure image embeddings. The two modalities are combined via Loss Trajectory Analysis for Uncertainty (LTAU)-based weighted fusion, which adaptively reweights features according to their predictive reliability. Across eight benchmark data sets, CUE achieved RMSE values ranging from 0.163 to 1.691 and outperformed affinity-based and quantitative structure–activity relationship (QSAR) baseline models. Furthermore, a virtual screening case study for EGFR­(T790M/C797S) inhibitors further demonstrated its utility for identifying selective hits. The source code is available at https://github.com/jjjabcd/CUE.

## Graphical abstract

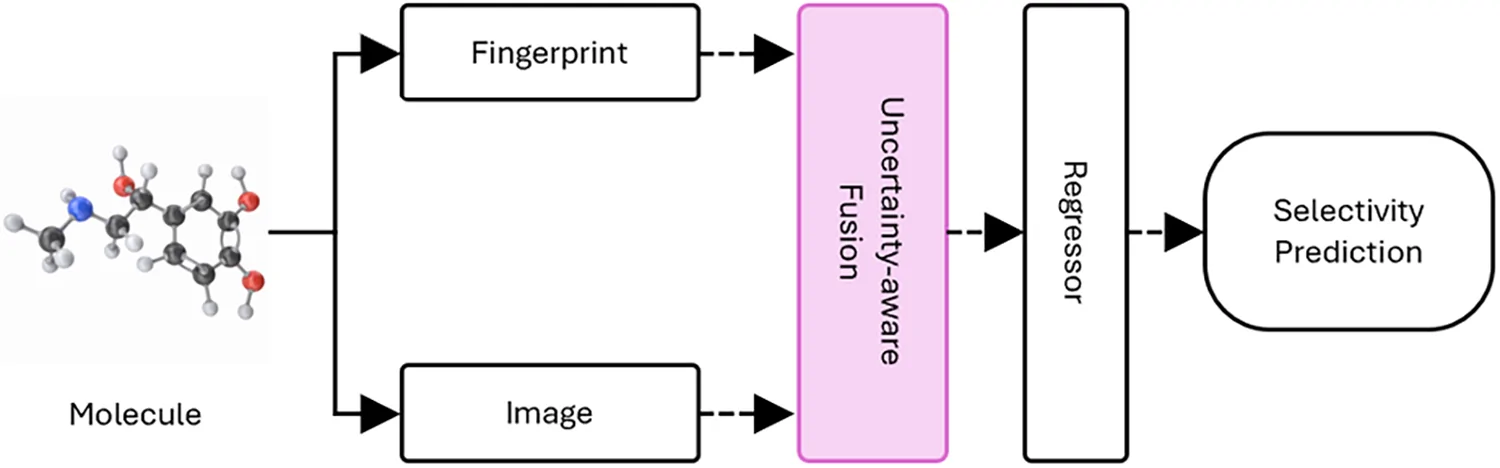

## Introduction

Artificial Intelligence (AI) technologies have emerged as powerful tools for various stages of drug discovery. By analyzing large-scale compound data sets and uncovering complex patterns between molecular structures and physicochemical properties, AI can substantially reduce the time and cost typically required for experimental workflows.^1^ Applications include virtual screening, de novo drug design, and the prediction of molecular properties.^2,3^

Kinase-targeted drug discovery has received particular attention due to the central role of kinases in cellular signaling and their relevance to numerous diseases, especially cancer.^4–6^ A critical challenge in developing kinase inhibitors is ensuring drug selectivity, which refers to the ability of a compound to bind with high affinity to its intended kinase target while minimizing interactions with off-target proteins.^7,8^ Figure 1 illustrates the concept of drug selectivity. Off-target binding can lead to toxic side effects and reduce therapeutic efficacy, and remains a persistent challenge in drug development.^9,10^ Selectivity is therefore a key determinant of both clinical translation and the rational design of kinase inhibitors targeting disease-relevant variants.

**Figure 1:**
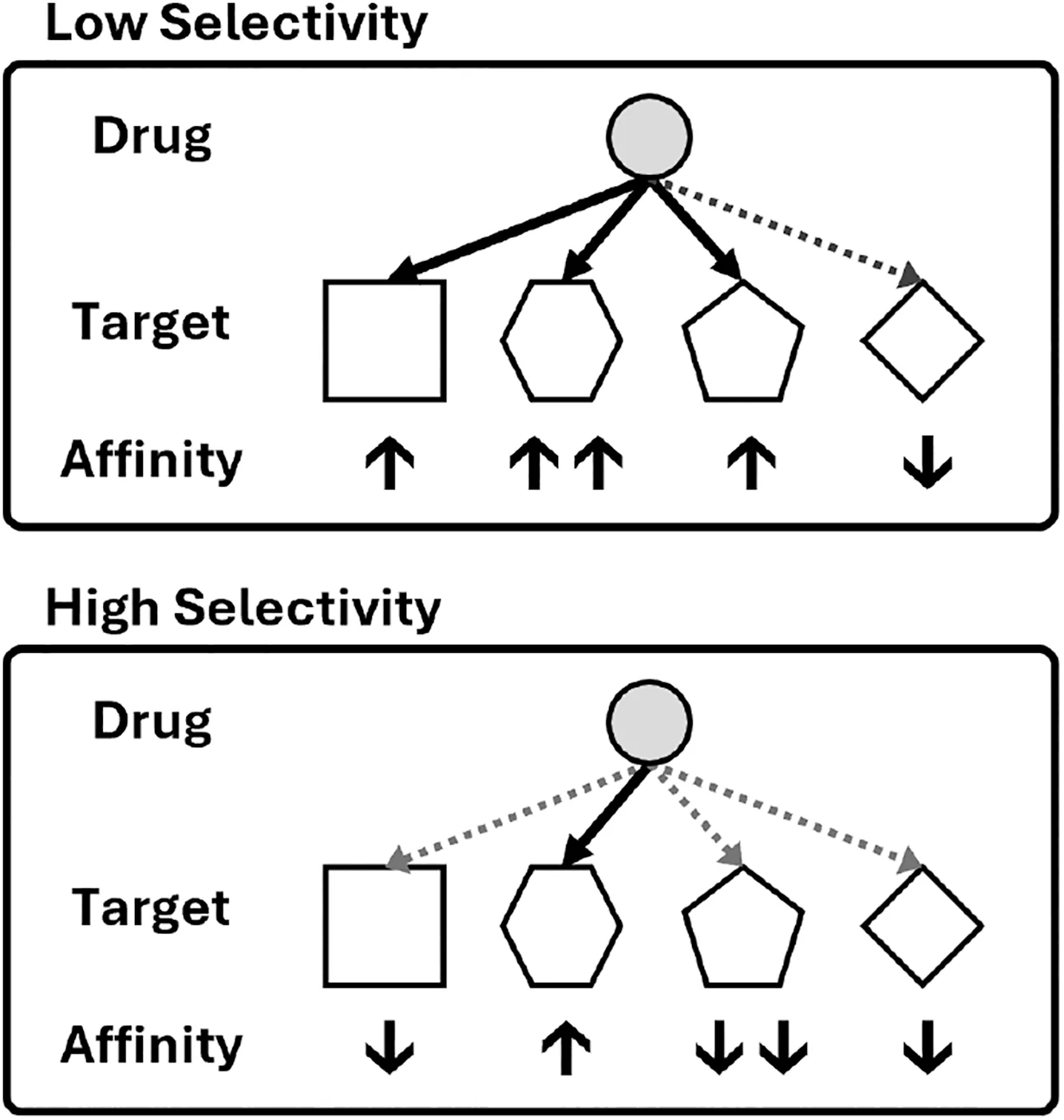
Conceptual example of selectivity in ligand-protein interactions.

Various quantitative metrics have been proposed to assess drug selectivity,^11^ including the Standard Selectivity Score (S), Partition Index (PI), Selectivity Entropy (Ssel), Window Score (WS), and Ranking Score (RS). These metrics are computed based on the binding affinities of a single compound measured across multiple kinases. In practice, evaluating drug selectivity therefore requires the simultaneous consideration of binding affinities across a broad kinase panel, which entails extensive experimental assays that are costly and time-consuming. To alleviate this burden, Drug-Target Affinity (DTA) models have been explored as a computational strategy.^12^ By predicting the binding affinities of a single compound against multiple kinases, DTA models enable indirect estimation of drug selectivity. However, this strategy is inherently limited by the accumulation of prediction errors across individual affinity estimates, which may compromise the reliability of the resulting selectivity scores. These limitations underscore the need for novel modeling approaches capable of directly predicting drug selectivity.

To address the challenges of drug selectivity prediction, we propose a Chemical Uncertainty-aware Embedding (CUE) framework that exploits molecular fingerprints and two-dimensional (2D) molecular structure images for drug selectivity prediction. CUE extracts modality-specific embeddings from molecular fingerprints and 2D molecular structure images, and combines them through uncertainty-aware weighted fusion based on Loss Trajectory Analysis for Uncertainty (LTAU).^13^ The unified embedding is then fed into an MLP-based prediction model to estimate drug selectivity. The main contributions of this study are summarized as follows:

- We propose CUE, a novel framework that directly predicts drug selectivity from molecular structures, circumventing the cumulative-error problem inherent in aggregating multiple DTA predictions.
- We design a multimodal feature integration scheme that exploits the complementary information of fingerprint-based and image-based molecular representations.
- We introduce an uncertainty-aware fusion mechanism based on LTAU, which adaptively weights each modality according to its predictive reliability.
- We comprehensively validate CUE across diverse selectivity metrics and benchmark datasets, and further demonstrate its practical utility through a case study on clinically challenging EGFR­(T790M/C797S) double mutant inhibitors.

## Related Works

### Drug-Target Affinity Prediction for Drug Selectivity

Drug selectivity can be indirectly estimated by aggregating DTA predictions across multiple kinases.^12^ Independently of the selectivity context, deep learning-based DTA models have advanced affinity prediction accuracy by leveraging diverse molecular representations of drugs and proteins, and these advances are expected to translate into improved reliability of DTA-derived selectivity estimates. DeepDTA^4^ proposed an architecture that independently encodes drug SMILES strings^14^ and protein sequences using 1D-CNNs to predict binding affinity. GraphDTA^15^ represented drugs as molecular graphs and employed Graph Neural Networks (GNNs) to more effectively capture structural information. MT-DTA^16^ combined convolutional and transformer architectures with an interactive learning mechanism to capture intra- and intermolecular substructure relationships from drug and protein sequences. TDGraphDTA^17^ applied diffusion-based graph optimization together with multiscale interaction learning to refine drug graph representations and the interaction between drug and target features. TEFDTA^6^ introduced a model that learns drug representations through molecular fingerprints and a transformer encoder, while additionally accommodating covalent binding interactions. MDCT-DTA^18^ represented drugs through multiscale graph diffusion and proteins through a hybrid convolutional–transformer network, and modeled drug–target relationships through interlayer feature interaction. RRGDTA^19^ introduced rotary positional encoding and an information retention mechanism to preserve contextual dependencies and drug–target association information. HMM-DTA^20^ leverages pretrained protein language models to enable efficient affinity prediction without explicit structural information on proteins. Although these models were not specifically designed for selectivity assessment, their per-target affinity estimates can serve as inputs to selectivity metrics, and improvements in their underlying accuracy are expected to propagate to more dependable selectivity scores.

Recent work has extended DTA-based affinity prediction to kinase inhibitor selectivity analysis. MMCLKin^12^ employed a multimodal contrastive learning framework that integrates a geometric graph network and a sequence-based network to improve kinase–inhibitor affinity prediction, and further evaluated kinase inhibitor selectivity from the resulting affinity profiles. However, in this paradigm, selectivity is inferred from predicted affinity profiles rather than serving as an explicit learning objective. Consequently, the model receives no direct supervision to learn structural features that distinguish on-target binding from off-target binding. Moreover, prediction errors from individual affinity estimates can accumulate when aggregated across a kinase panel, further degrading the reliability of the resulting selectivity scores.

### QSAR Methods for Drug Selectivity

Quantitative Structure–Activity Relationship (QSAR) models^21,22^ have been widely studied to predict molecular properties from chemical structures, and diverse molecular representations have been employed to model properties such as toxicity, solubility, and binding affinity. These representations include molecular fingerprints (FPs),^23^ SMILES strings,^14^ molecular structure images,^24^ and molecular graphs.^25^ Although most QSAR studies have targeted general molecular properties rather than selectivity as a direct prediction target, the same modeling paradigm can be naturally extended to selectivity prediction.

FP-based models utilize handcrafted descriptors such as Extended-Connectivity Fingerprint (ECFP)^23^ or Molecular ACCess System (MACCS)^26^ and typically employ machine learning or Multi-Layer Perceptrons (MLPs) to learn structure–property relationships from bit-vector FPs. For example, FANN-QSAR^27^ demonstrated the successful use of MLPs with various FPs for virtual screening, and Ding et al.^28^ combined molecular fingerprints with gradient boosting to predict ionic liquid properties, further interpreting the resulting models with Shapley Additive exPlanations (SHAP).^29^

SMILES-based models treat molecular structures as sequential text. Early approaches employed recurrent architectures, such as Recurrent Neural Networks (RNNs)^30^ and Long Short-Term Memory (LSTM) networks. For instance, DeepCDA^31^ combines a CNN-LSTM encoder with domain adaptation to predict affinities across domains. To better capture global context within SMILES sequences, attention-based architectures have subsequently emerged. Transformer-based models such as ChemBERTa^32^ and SMILES-BERT^33^ have adopted a chemical language modeling perspective, deriving contextual representations through pretraining on large SMILES corpora.

Image-based approaches use CNNs to extract features directly from 2D molecular depictions, mapping visual patterns to predicted properties. For instance, Zhong et al.^34^ developed a molecular-image-CNN model and used Grad-CAM for model interpretability. Similarly, Shi et al.^35^ applied CNNs to 2D molecular images for ADMET property prediction.

Graph-based models represent molecules as graphs with atoms and bonds corresponding to nodes and edges. GNNs are then employed to learn molecular representations through iterative message passing across the graph. For instance, ADMET-AI^36^ combines a GNN-based molecular encoder with physicochemical descriptors computed by RDKit,^37^ such as molecular weight, log *P*, and ring count, and has demonstrated strong performance across diverse ADMET property prediction tasks.

While these single-modality approaches have shown promising performance for general property prediction, each individual representation captures only one aspect of molecular structure. FPs encode predefined substructural patterns but lack continuous or spatial information; SMILES strings convey atomic connectivity in a linear form but obscure the geometric arrangement of atoms; 2D images preserve spatial layout but do not explicitly encode chemical bond properties or substructural identities; and graph representations effectively capture topological connectivity but may not fully capture the visual structural patterns that 2D depictions provide. The complex structure-selectivity relationships, which involve subtle differences among highly similar kinase targets, may be difficult to fully resolve from any single representation alone, suggesting that integrating complementary modalities can provide a more comprehensive basis for selectivity modeling.

### Multimodal QSAR Approaches

Multimodal QSAR approaches integrate diverse molecular representations to leverage complementary information from different structural views of a molecule. As each representation captures different aspects of chemical structure, combining multiple modalities through feature fusion or joint embedding learning can provide a more comprehensive basis for molecular property prediction than using a single representation alone.

Early multimodal QSAR approaches focused on combining structurally complementary representations through relatively simple fusion strategies. MMRLFN^38^ integrates molecular graphs and SMILES sequences through concatenation-based cascade fusion, combining topological information with sequence-level features. Beyond combining structural representations alone, SFMMoE^39^ integrates molecular graph representations with semiempirical molecular descriptors through a multiexpert network, demonstrating that augmenting learned representations with complementary descriptor information can improve molecular property prediction. MMFDL^40^ extends this idea to three modalities (SMILES, ECFP fingerprints, and molecular graphs) and employs a late fusion strategy in which the contribution of each modality is determined by machine learning-based weighting methods. Techniques for integrating heterogeneous representations are also being actively developed in other fields, such as biological interaction modeling^41^ and data augmentation.^42^

More recent approaches have explored richer interaction modeling and broader modality coverage. MCMPP^43^ incorporates 3D molecular conformations alongside SMILES, fingerprints, and graphs, and uses a cross-attention mechanism to model interactions among heterogeneous representations. MMFRL^44^ further broadens the modality set to include molecular images and NMR-based features, and systematically investigates early, intermediate, and late fusion strategies, demonstrating that the optimal fusion stage can vary depending on the downstream task.

These studies demonstrate that integrating heterogeneous molecular representations can improve molecular property prediction by capturing complementary structural information. However, many existing multimodal QSAR approaches rely on predefined or fixed fusion schemes, where modality contributions are determined globally and remain fixed across all input compounds after training. Although such strategies can improve overall predictive performance, they do not explicitly adjust modality weights for each input compound according to molecule-wise modality reliability. This limitation is particularly important because the most informative modality may differ across compounds depending on whether molecular information is better represented by FP-based substructural patterns, sequence-like representations, graph topology, molecular images, or 3D conformational information. Therefore, adaptive fusion strategies that estimate modality reliability at the sample level and assign compound-specific modality weights are needed for more flexible integration of heterogeneous molecular representations.

### Uncertainty Quantification for Adaptive Feature Fusion

Uncertainty Quantification (UQ)^45^ provides a principled framework for estimating the reliability of model predictions under conditions such as noisy inputs, limited training data, or out-of-distribution samples. Recent studies have extended to use uncertainty estimates beyond their conventional use for prediction confidence to enable for adaptive feature fusion. In uncertainty-aware fusion,^46,47^ modality contributions are dynamically adjusted for each input according to their estimated reliability, reducing the influence of less reliable modalities when forming a unified representation.

Among UQ approaches, feature-based methods are particularly relevant to adaptive feature fusion as they estimate reliability from latent representations rather than from final prediction outputs alone. These methods typically assess how well an input sample conforms to the training data distribution in the learned feature space, assigning higher uncertainty to samples that deviate from well-represented regions of the feature space.^48^

LTAU^13^ is a feature-based uncertainty estimation method that incorporates training dynamics. LTAU estimates uncertainty by jointly considering the position of an input in the embedding space and the variation of its loss trajectory during training. This enables LTAU to quantify the model’s reliability on a per-sample basis.

This combination of feature-space awareness and training-dynamics sensitivity make LTAU suitable for estimating modality-specific reliability in uncertainty-aware molecular feature fusion. In drug selectivity prediction, the reliability of each molecular modality can vary across compounds. Therefore, LTAU-derived uncertainty can serve as a molecule-wise modality reliability score, enabling adaptive weighting of heterogeneous molecular representations. This insight motivates the uncertainty-aware fusion strategy at the core of CUE. In the following section, we describe how CUE integrates molecular fingerprints and 2D structural images through LTAU-guided weighted fusion to predict compound-level selectivity.

## Methods

CUE requires two types of molecular input data, FPs and 2D molecular structure images (Figure 2). For feature extraction, CUE uses RDKit^37^ to compute FP feature vectors and the MolScribe encoder^24^ to extract molecular image feature vectors from the molecular structure images. These feature vectors are then mapped into modality-specific embeddings through dedicated MLPs trained to encode selectivity-related molecular information. CUE integrates the two embeddings into a unified embedding through uncertainty-aware weighted summation, where the fusion weights are derived from per-modality uncertainty quantified by LTAU^13^ and normalized to sum to one. The unified embedding is then used to predict drug selectivity scores.

**Figure 2:**
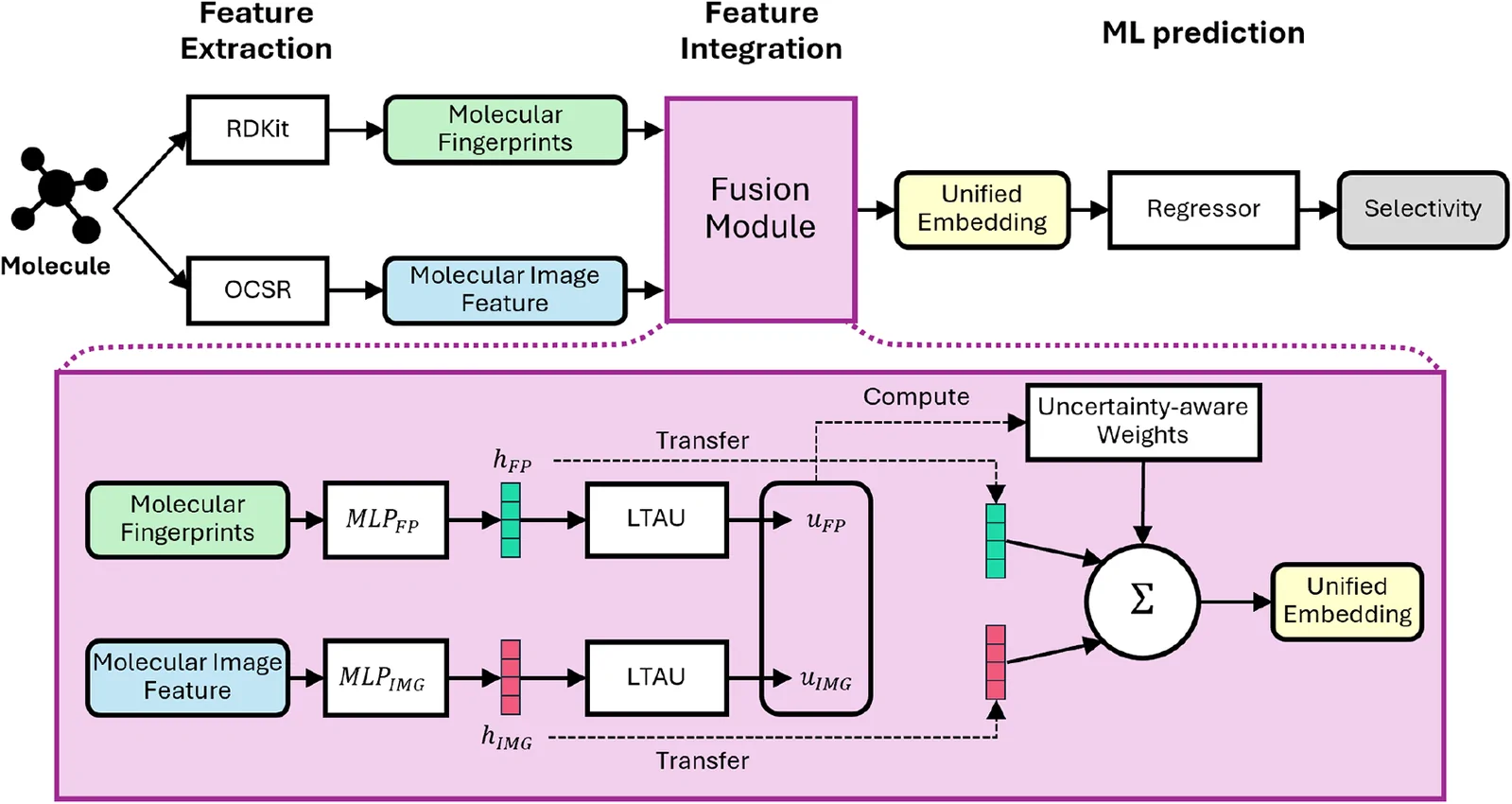
Overview of the proposed CUE framework. Multimodal features from fingerprints and molecular images are extracted by pretrained models and integrated through uncertainty-aware weighted summation for selectivity prediction.

To support this architecture, CUE follows a two-stage training procedure. The first stage trains the modality-specific MLPs on individual molecular modalities and simultaneously collects the embeddings and per-sample loss trajectories needed to construct LTAU-based uncertainty estimates. The second stage uses these uncertainty estimates to assign molecule-wise modality weights for feature fusion, and a final regressor is trained on the resulting unified embeddings to predict drug selectivity scores.

### Molecular Feature Extraction

#### First Modality: Fingerprints

FPs are widely used molecular descriptors that encode structural characteristics of compounds as bit vectors. A bit value of 1 indicates that the molecule contains the substructure or topological feature represented by that bit. Through this bit-level encoding, FPs provide explicit information on the presence or absence of specific chemical substructures, making them useful molecular representations for drug selectivity prediction.

CUE uses ECFP4 fingerprints as one of the two molecular modalities, given their widespread adoption in QSAR studies for molecular property prediction. The fingerprints are computed using RDKit’s Morgan fingerprint generator with a radius of 2 and a length of 2,048 bits.

#### Second Modality: Molecular Image

CUE leverages Optical Chemical Structure Recognition (OCSR) to directly exploit 2D molecular image representations. OCSR is a technique for recognizing chemical structures from 2D molecular images, and its performance has been significantly advanced by recent DL models.^24,49,50^ In this study, we utilized the encoder of MolScribe,^24^ which was pretrained on the large-scale PubChem database.^51^ This enabled CUE to extract 1024-dimensional image features that capture visual and spatial patterns in 2D molecular depictions, complementing the substructure-level information encoded by fingerprints.

### Uncertainty Quantification via LTAU

To quantify the uncertainty of each molecular representation modality, we employed LTAU.^13^ As heterogeneous molecular modalities can show different levels of reliability depending on the molecule, CUE quantifies modality-specific uncertainty and uses it to determine the contribution of each modality in the feature fusion process.

CUE first trains a modality-specific MLP *f* for each modality to predict per-modality selectivity scores from the corresponding molecular feature vector, using mean squared error (MSE) loss. This supervised training serves two purposes: it produces a modality-specific embedding space (from the MLP’s last hidden layer) that encodes selectivity-related molecular information, and it generates per-sample loss trajectories from which LTAU-based uncertainty estimates are derived.

The modality-specific MLPs consist of three hidden layers with ReLU activations, progressively reducing the input feature dimensionality. For the FP modality, the 2,048-dimensional ECFP4 feature vector is mapped through hidden dimensions of 512, 256, and 32. For the molecular image (IMG) modality, the 1,024-dimensional MolScribe feature vector is mapped through hidden dimensions of 256, 128, and 32. The 32-dimensional output of the last hidden layer serves as the modality-specific embedding, denoted as emb­(*x*, *f*) for molecule *x*.

LTAU estimates the predictive uncertainty of a molecule from how its prediction error evolves over the course of training. The loss trajectory of molecule *x*_*i*_ is defined in eq 1

1 $$T_{i}=\left\{\epsilon_{i,1},\epsilon_{i,2},...,\epsilon_{i,E}\right\}$$

where *E* is the total number of training epochs and ϵ_*i*,*t*_ = |*y*_*i*_ – *f* ^(*t*)^(*x*_*i*_)| denotes the prediction error of molecule *x*_*i*_ at epoch *t*, with *f* ^(*t*)^ representing the modality-specific MLP after the *t*-th training epoch.

Each loss trajectory is converted into a discretized empirical error distribution using histogram binning. The bin edges are shared across all molecules within the same modality and training fold, rather than defined per molecule. Specifically, we first collect the loss trajectory set $T=\left\{T_{1},...,T_{N}\right\}$ from all *N* training molecules, and then define a shared set of *B* + 1 bin edges, denoted as *edges* = {*a*_0_, *a*_1_, ..., *a*_*B*_}, from the range of prediction errors observed in T , following the histogram-based Probability Density Function (PDF) construction used in LTAU.

Using these shared bin edges, $\mathrm{Histogram}\left(T_{i},\mathrm{edges}\right)$ returns a discrete probability distribution *p*_*i*_ = {*p*_*i*,*b*_}_*b*=1_^*B*^ over the shared error bins, where *p*_*i*,*b*_ denotes the empirical probability, computed as the fraction of errors in $T_{i}$ that fall within the *b*-th shared error interval [*a*_*b*–1_, *a*_*b*_). As all reference distributions {*p*_*i*_}_*i*=1_^*N*^ are defined on the same error support, they can be averaged bin-wise when estimating the error distribution of a query molecule. In addition, the embedding *h*_*i*_ = emb­(*x*_*i*_, *f*) is stored for nearest neighbor search. Following LTAU, we refer to these discretized empirical error distributions as empirical PDFs. The empirical PDFs and embeddings constructed from the training molecules form the reference PDF set P and the reference embedding set H , respectively. The construction of these reference sets is summarized in Algorithm 1.
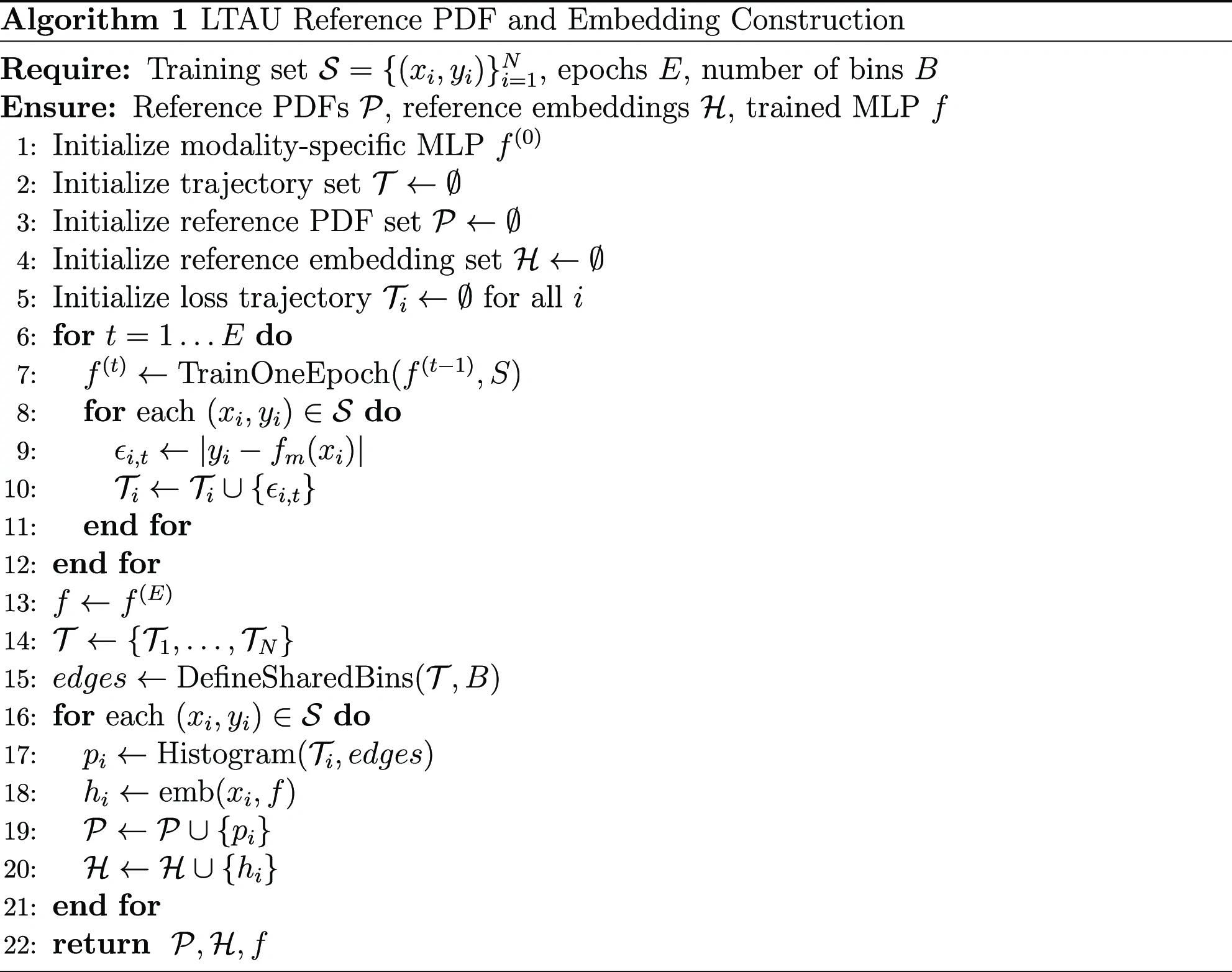


To quantify the uncertainty of a new query molecule *x*, CUE obtains its embedding *h* = emb­(*x*, *f*_*m*_) and identifies the index set N of its *K*-nearest reference embeddings in H . Since all reference PDFs share the same error-bin support, the query error distribution *p̅* can be estimated by averaging the nearest-neighbor PDFs over the shared bins. The uncertainty of molecule *x* is then quantified as the Shannon entropy of the aggregated empirical error distribution *p̂*. A higher entropy indicates a more dispersed error distribution across the shared error bins, corresponding to higher uncertainty in representing molecule *x*. The procedure for quantifying LTAU-based uncertainty is summarized in Algorithm 2. The quantified uncertainty *u* is used to determine the modality contribution in the feature fusion process.
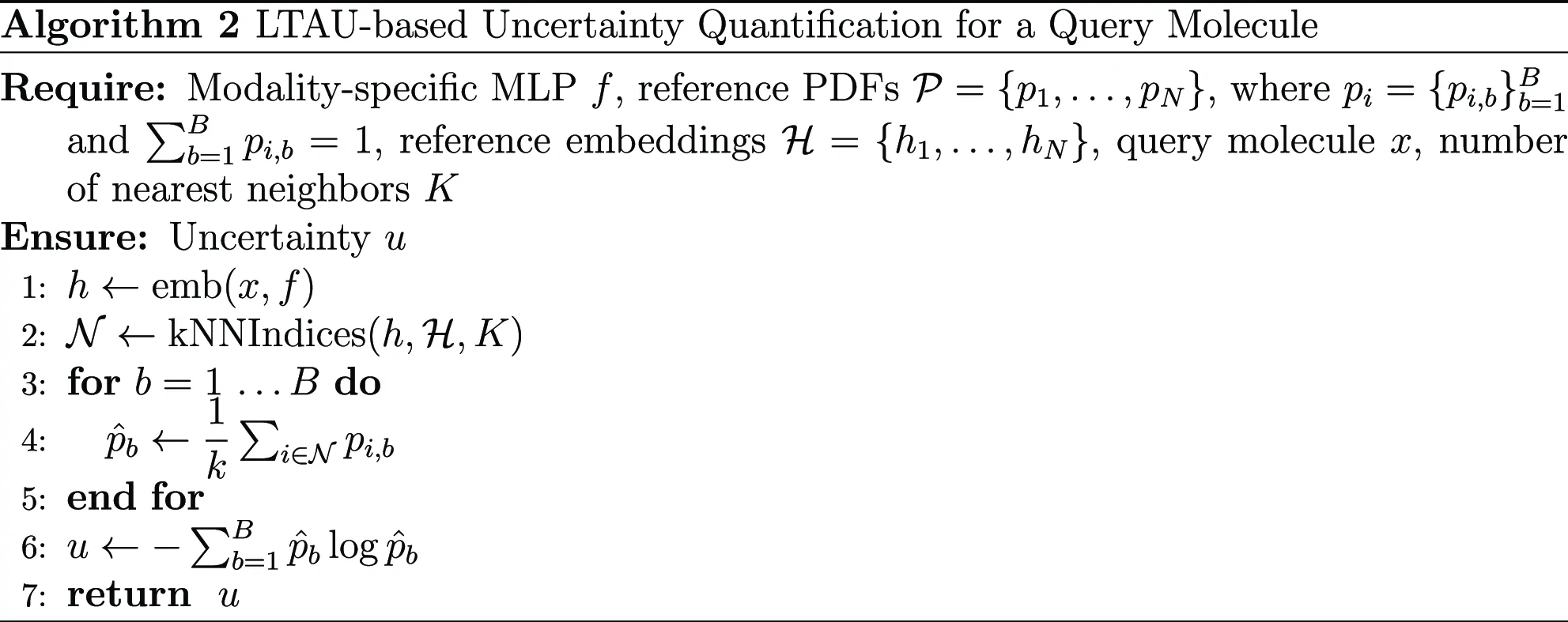


### Chemical Uncertainty-Aware Embedding (CUE)

The LTAU-based uncertainty quantifies how reliably each modality represents a given molecule, and CUE leverages this signal to combine the two modalities through an uncertainty-aware weighted summation, so that more reliable modalities contribute more strongly to the fused representation on a per-molecule basis (Figure 2). Because ECFP4 FPs and the molecular image features extracted by MolScribe have different dimensionalities and reside in different feature spaces, CUE first maps both modalities to embeddings of a common dimensionality (32) using the last hidden layers of the modality-specific MLPs, the same networks from which the LTAU-based uncertainty estimates are obtained.

Given the modality-specific embeddings *h*_FP_ and *h*_IMG_, CUE produces a unified embedding as defined in eq 2

2 $$h_{\mathrm{uni}}=w_{\mathrm{FP}}\cdot h_{\mathrm{FP}}+w_{\mathrm{IMG}}\cdot h_{\mathrm{IMG}}$$

The adaptive fusion weights are computed according to eq 3.

3 $$w_{i}=\frac{\exp\left(-u_{i}\right)}{\sum_{j\in \left\{\mathrm{FP},\mathrm{IMG}\right\}}\exp\left(-u_{j}\right)},i\in \left\{\mathrm{FP},\mathrm{IMG}\right\}$$

Under this uncertainty normalization, a modality receives a weight inversely related to its uncertainty, so that more reliable modalities contribute more strongly to the unified embedding. This enables molecule-wise adaptive feature fusion based on modality reliability and produces an uncertainty-aware unified embedding.

### Drug Selectivity Prediction Model

The unified embedding *h*_uni_ is passed to a selectivity regressor that predicts the final selectivity score. The regressor is implemented as an MLP consisting of three hidden layers with dimensions of 16, 16, and 8, each followed by a ReLU activation, and a final output unit. We train the regressor using the Adam optimizer with the MSE loss.

## Results

### Experimental Setup

#### Benchmark Data Sets

We constructed benchmark data sets from the ChEMBL v35 database,^52^ which provides experimentally measured compound–target affinity values, including dissociation constant (*K*_d_) and inhibition constant (*K*_i_) values. Based on these two affinity measurements, we independently constructed the DIS data set from *K*_d_ values and the INH data set from *K*_i_ values. The fold-wise sample counts for the DIS and INH data sets used in 5-fold cross-validation are summarized in Supporting Table S1. As drug selectivity can be quantified using multiple complementary metrics, we constructed eight benchmarks from the collected data, four for DIS and four for INH, using four selectivity metrics:^11^ PI, Ssel, WS, RS. Each benchmark is named using the Data set-Metric convention, such as DIS-PI and INH-PI.

Through this data collection and benchmark construction process, we obtained 556 compounds for DIS and 1,824 compounds for INH. Detailed data collection and preprocessing procedures, the formal definitions of the selectivity metrics, and the parameter settings used for WS and RS are provided in Supporting Information, Section A1.

#### Implementation Details

In this study, prediction models were implemented using PyTorch (v1.12.1). MolScribe^24^ was used to extract molecular image features. Molecular fingerprints were generated with RDKit (v2023.03.3),^37^ and scikit-learn (v1.3.2)^53^ was used for evaluation, cross-validation splitting, and nearest neighbor search. All experiments and result analysis were conducted on Ubuntu 24.04 with an NVIDIA GeForce RTX 4080 GPU.

#### Baseline Description

Eight models were used as baselines for selectivity prediction: four DTA-based models and four QSAR-based models. The DTA baselines are DeepDTA,^4^ GraphDTA,^15^ TEFDTA,^6^ and HMM-DTA,^20^ which predict target-specific binding affinities from which selectivity is subsequently derived. The QSAR baselines are ADMET-PrInt,^25^ ADMET-AI,^36^ MMFDL,^40^ and MMFRL,^44^ which predict selectivity directly from molecular representations. These four were selected to span the design choices most relevant to the present work: ADMET-PrInt operates on a single molecular representation, whereas MMFDL and MMFRL combine multiple representations through fixed-weight late fusion and through concatenation into a single embedding vector, respectively. Detailed descriptions of all baseline models are provided in Supporting Table S2.

#### Evaluation Metrics

The performance of the prediction models was evaluated using three metrics. Root Mean Squared Error (RMSE) measures the average magnitude of prediction errors, where low RMSE values indicate small prediction errors. As shown in eq 4, RMSE is calculated by squaring the differences between the true value *y*_*i*_ and predicted value *ŷ*_*i*_, averaging those squared differences, and then taking the square root.

4 $$\mathrm{RMSE}=\sqrt{\frac{1}{n}\sum_{i=1}^{n}{\left(y_{i}-{ŷ}_{i}\right)}^{2}}$$

The coefficient of determination (*R* ^2^) quantifies the proportion of variance in the true values that is explained by the model predictions. An *R* ^2^ value closer to 1 indicates better goodness of fit. The *R* ^2^ score is calculated as shown in eq 5

5 $$R^{2}=1-\frac{\sum_{i=1}^{n}{\left(y_{i}-{ŷ}_{i}\right)}^{2}}{\sum_{i=1}^{n}(y_{i}-\mathrm{y̅}{)}^{2}}$$

where *y*_*i*_ denotes the true value, *ŷ*_*i*_ denotes the predicted value, and *y̅* denotes the mean of the true values.

Pearson’s Correlation Coefficient (PCC) measures the linear correlation between predicted and true values, with values ranging from −1 to 1. Positive PCC values indicate positive linear correlation, negative values indicate negative linear correlation, and values close to 0 indicate weak or no linear correlation. PCC is calculated as in eq 6, where μ and μ̂ denote the mean of the true values and the predicted values, respectively.

6 $$\mathrm{PCC}=\frac{\sum_{i}\left(y_{i}-\mu \right)\left({ŷ}_{i}-\mathrm{\mu ̂}\right)}{\sqrt{\sum_{i}{\left(y_{i}-\mu \right)}^{2}}\sqrt{\sum_{i}{\left({ŷ}_{i}-\mathrm{\mu ̂}\right)}^{2}}}$$

### Prediction Performance Comparison

We compared CUE with the four DTA-based models and four QSAR-based baselines on the eight benchmark data sets. For DTA-based baselines, selectivity metrics were computed from predicted affinity values over multiple kinases for each compound. To ensure a fair assessment of generalization performance, compound-level 5-fold cross-validation was conducted for all models. The experimental procedures for DTA-based and QSAR-based models are illustrated in Supporting Figure S1, and the compound-level cross-validation scheme is described in Supporting Figure S2.

#### Performance on Benchmark Data Sets

CUE achieved the lowest RMSE across all eight benchmarks, outperforming both the DTA-based and QSAR-based baselines (Figure 3(a–h)). Relative to the strongest baseline on each benchmark, the reduction in prediction error ranged from 0.2% to 8.4%, with the largest reductions on DIS-WS2 (8.4%) and DIS-PI (6.2%); on DIS-RS2 and INH-Ssel the margin over the closest baseline fell within one standard deviation across folds. CUE also achieved the highest *R* ^2^ on all eight benchmarks (Supporting Tables S3 and S4).

**Figure 3:**
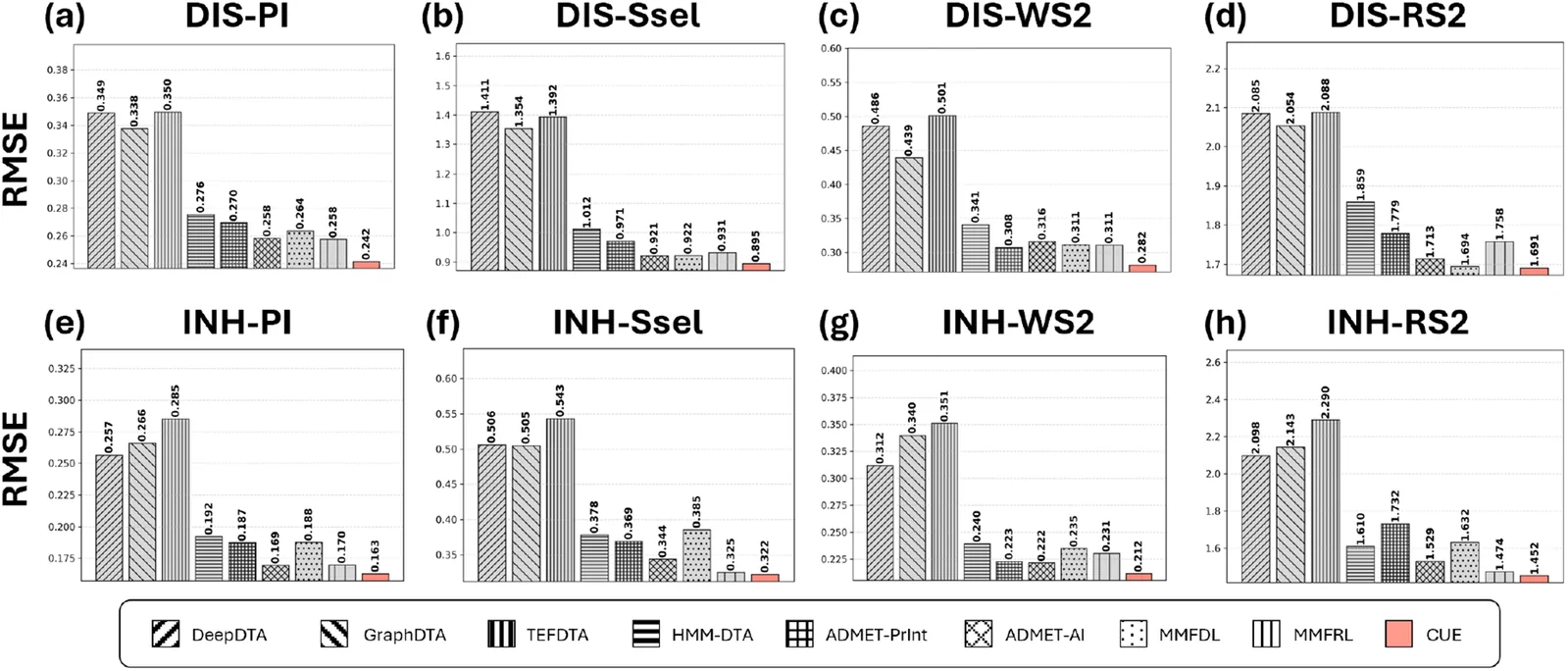
Comparison of RMSE values in drug selectivity prediction across DTA-based and QSAR-based baseline models. (a–d) DIS benchmarks (DIS-PI, DIS-Ssel, DIS-WS2, DIS-RS2), (e–h) INH benchmarks (INH-PI, INH-Ssel, INH-WS2, INH-RS2).

The four DTA-based baselines infer selectivity indirectly from per-target affinity predictions, whereas CUE learns selectivity as a direct prediction target. CUE achieved lower RMSE than every DTA-based baseline on every benchmark, and the *R* ^2^ values of these baselines were negative on most benchmarks, indicating that their predictions did not track the scale of the selectivity values. Their PCC values were nonetheless high on several benchmarks, exceeding those of all QSAR-based models on DIS-PI and DIS-Ssel (Supporting Table S3). Indirectly derived predictions can therefore recover the relative ordering of compounds while failing to reproduce the magnitude of selectivity, which limits their utility when calibrated selectivity estimates are required.

Among the QSAR-based baselines, CUE outperformed the single-modality ADMET-PrInt as well as the two multimodal models, MMFDL and MMFRL. These two models fuse modalities differently—MMFDL through fixed-weight late fusion and MMFRL through concatenation into a single embedding vector—but in both cases the fusion scheme is fixed after training and applies uniformly to every input molecule, whereas CUE weights each modality according to its per-molecule uncertainty. MMFRL moreover draws on six molecular representations (fingerprints, SMILES strings, molecular graphs, molecular images, NMR spectrum, and NMR peak), a broader set than the two used by CUE, which indicates that the advantage observed here follows from how modalities are weighted rather than from how many are available.

Complete performance metrics are provided in Supporting Table S3 for the DIS benchmarks and Supporting Table S4 for the INH benchmarks. Among the QSAR-based baselines, CUE ranked highest in PCC on five of the eight benchmarks, whereas MMFRL attained marginally higher values on DIS-PI, DIS-Ssel, and INH-RS2. The advantage of CUE is thus most consistent in the accuracy of the predicted selectivity values, whereas rank agreement varies across benchmarks and metrics.

#### Performance on an External Test Set

To evaluate whether the proposed model generalizes to data generated by a different experimental source, we constructed an external test set from the DAVIS data set,^8^ in which 72 inhibitors were profiled against a kinome-wide kinase panel. DAVIS reports *K*_d_ values and therefore shares the same affinity type as the DIS benchmarks, which were constructed from *K*_d_ values collected from ChEMBL; drug selectivity labels could thus be computed directly from the DAVIS measurements without unit conversion.

Selectivity metrics depend on the number and identity of the kinases measured for each compound, so differences in panel composition between the two resources propagate into the resulting selectivity labels. We therefore compared the label distributions obtained from the DIS and DAVIS data sets for each metric (Supporting Figure S3). The distributions of PI, Ssel, and WS2 differed substantially between the two data sets, whereas those of RS2 largely overlapped—an outcome consistent with the definition of RS2, which is computed from the relative ranking of kinase affinities rather than from their absolute values. The external evaluation was accordingly restricted to RS2.

The models trained on the DIS-RS2 benchmark were applied to the external test set without additional training or fine-tuning. Predictions were obtained from the five models produced by compound-level 5-fold cross-validation, and the reported performance is the average across folds. Table 1 reports the predictive performance on the external test set.

**Table 1:** Predictive Performance of CUE and the Baseline Models on the External Test Set Derived from the DAVIS Data set, Evaluated by RMSE and PCC^a^

Category	Model	RMSE (↓)	PCC (↑)
DTA	DeepDTA	1.184 ± 0.002	0.143 ± 0.039
	GraphDTA	1.150 ± 0.072	0.241 ± 0.045
	TEFDTA	1.173 ± 0.007	0.216 ± 0.156
	HMM-DTA	1.111 ± 0.065	–0.002 ± 0.089
QSAR	ADMET-PrInt	1.144 ± 0.127	0.096 ± 0.072
	ADMET-AI	**0.982** ± **0.023**	0.224 ± 0.081
	MMFDL	1.082 ± 0.065	0.029 ± 0.114
	MMFRL	1.052 ± 0.155	0.605 ± 0.128
	CUE	1.044 ± 0.179	**0.606** ± **0.147**

a Values are reported as mean ± standard deviation across the five models obtained from compound-level 5-fold cross-validation on the DIS-RS2 benchmark. Bold indicates the best performance and underlining the second best for each metric.

The QSAR-based models achieved substantially lower RMSE (0.982–1.144) than the DTA-based models (1.111–1.184), indicating that predicting drug selectivity directly was more effective on this external test set than deriving it from separately predicted target-specific affinities.

Among the QSAR-based models, ADMET-AI attained the lowest RMSE (0.982), followed by CUE (1.044) and MMFRL (1.052); these values differ by less than the fold-to-fold variability observed for CUE and MMFRL. The models separated more clearly in rank agreement: CUE (0.606) and MMFRL (0.605) achieved comparable correlations, whereas no other model exceeded 0.241. ADMET-AI combined the lowest RMSE with a PCC of 0.224, indicating that its predictions were close to the reference values in absolute terms but did not reproduce the relative ordering of compound selectivity. Absolute error values are not directly comparable between the internal and external evaluations, as the two data sets differ in size and composition. Across both settings, however, CUE was the only model that ranked among the leading models in both RMSE and PCC, whereas the remaining models led on one metric in only one setting.

### Analysis of CUE Embeddings via UMAP

CUE generates a unified embedding vector by integrating modality-specific embeddings. To visually evaluate whether this embedding better captures selectivity-related patterns compared to baseline embeddings, we compared it with embeddings extracted from QSAR-based baseline models using Uniform Manifold Approximation and Projection (UMAP). The embedding extraction procedures for each model are provided in Supporting Figure S4.

Figure 4 shows the UMAP results for DIS-PI (a–e) and INH-PI (f–j). Each compound is represented by a point colored on a blue-to-red scale according to its selectivity score, with red indicating higher selectivity.

**Figure 4:**
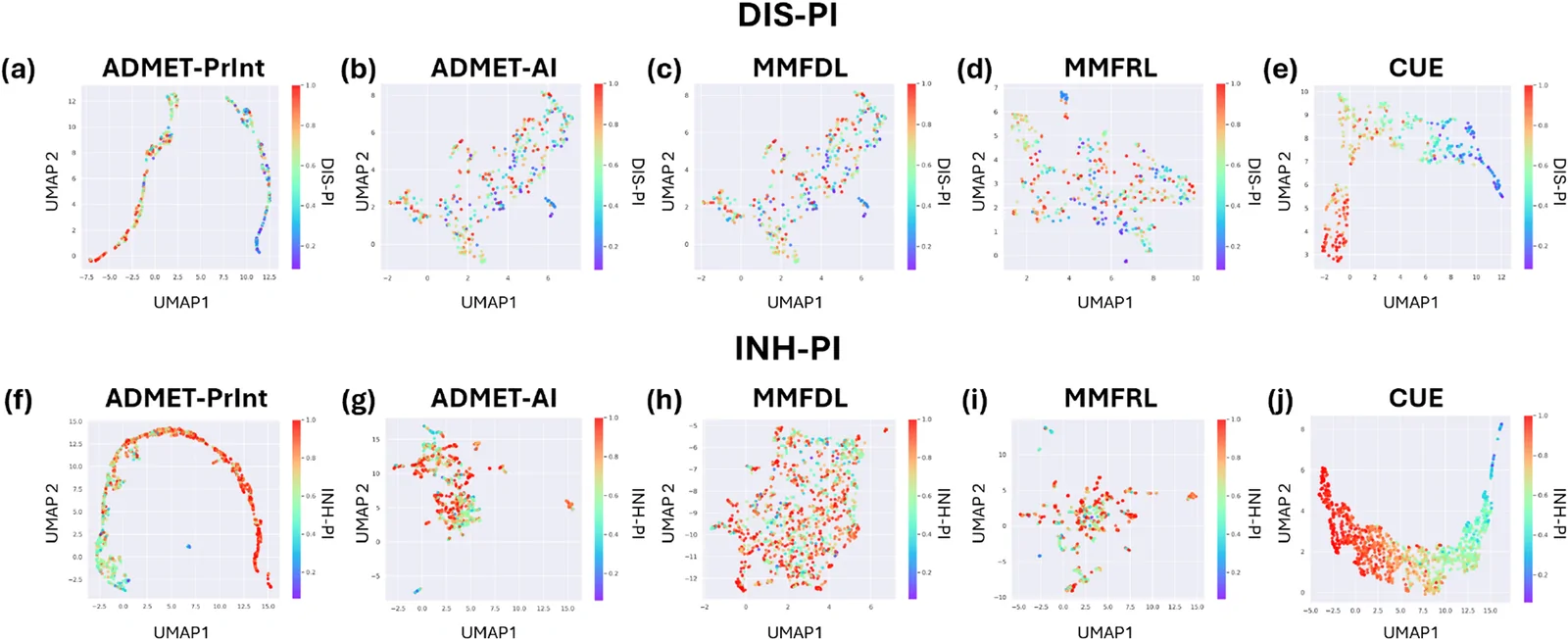
UMAP visualization of molecular embeddings extracted from different models on two benchmarks. (a–e) DIS-PI results for ADMET-PrInt, ADMET-AI, MMFDL, MMFRL, and CUE, respectively. (f–j) INH-PI results for ADMET-PrInt, ADMET-AI, MMFDL, MMFRL, and CUE, respectively. Each point represents a molecule. Colors indicate the DIS-PI score in (a–e) and the INH-PI score in (f–j).

CUE embeddings show a clearer organization aligned with selectivity scores than baseline embeddings (Figure 4). Embeddings extracted from ADMET-AI, MMFDL, and MMFRL exhibit relatively dispersed distributions without a clear color gradient along the projection, whereas ADMET-PrInt forms a more structured embedding distribution but contains compounds with different selectivity scores intermingled within the same regions. In contrast, CUE embeddings form a smooth gradient along selectivity scores, suggesting that the embedding space systematically encodes selectivity-related information. Visualization results for additional benchmarks are provided in Supporting Figures S5–S6.

### Analysis of Uncertainty-Based Modality Weight

The CUE framework adopts an uncertainty-aware weighting strategy that dynamically adjusts the contribution of each modality based on its uncertainty. To verify the validity of this strategy, we analyzed how the relative magnitude of uncertainty between modalities relates to selectivity prediction. The relative magnitude of uncertainty between the two modalities is quantified as a single metric, *r*, computed as follows

7 $$r=\log_{2}\left(\frac{u_{\mathrm{FP}}}{u_{\mathrm{IMG}}}\right)$$

where *u*_FP_ and *u*_IMG_ denote the uncertainty values of the fingerprint modality and the molecular image modality, respectively. *r* represents the disparity in uncertainty between the two modalities, where lower values of *r* indicate that the uncertainty of the fingerprint modality is relatively lower than that of the molecular image modality.

The following analysis was conducted on the DIS-PI data set. Figure 5(a) presents a UMAP visualization of the CUE embedding space, in which each compound is colored according to its corresponding *r* value. The values of *r* exhibit substantial variation across compounds, suggesting that the relative reliability between the two modalities is compound-dependent rather than being consistently dominated by a single modality.

**Figure 5:**
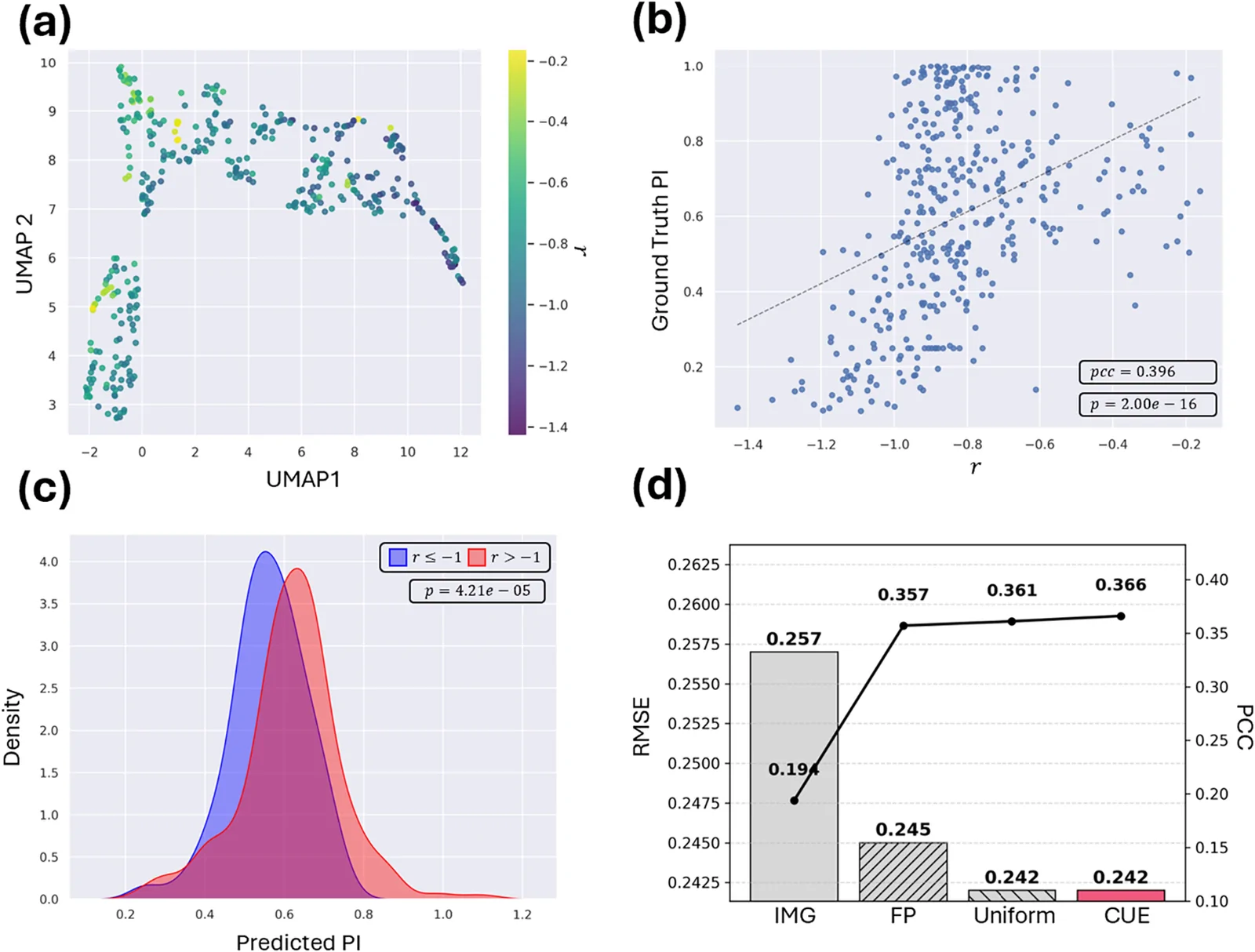
Analysis of multimodal uncertainty and selectivity prediction on the DIS-PI data set. (a) UMAP visualization of compound embeddings colored by the uncertainty ratio *r*. (b) Pearson correlation analysis between the uncertainty ratio and ground-truth PI values. (c) Kernel Density Estimation (KDE) of predicted PI values grouped by the uncertainty ratio threshold *r* = −1. (d) RMSE and PCC comparison across molecular image-only (IMG) fingerprint-only (FP), equal-weighting (Uniform), and CUE models.

Figure 5(b) presents the analysis of the linear relationship between *r* and the ground-truth PI values. Correlation analysis revealed a statistically significant positive relationship (PCC = 0.396, *p* = 2.00 × 10^–16^). We observed that compounds with lower *r* values tended to exhibit low ground-truth selectivity. This suggests that compounds whose FP uncertainty is much lower than their molecular image uncertainty are often less selective. Compounds with high selectivity were mainly observed in regions where the two modalities have similar uncertainty values, suggesting that both modalities may provide useful information for selective compounds.

To further examine this pattern, Figure 5(c) compares the predicted PI distributions of two compound groups partitioned at *r* = −1. This threshold naturally corresponds to the point at which the FP uncertainty is half of the molecular image uncertainty in log_2_ scale. A two-sided *t*-test yielded a *p* = 4.21 × 10^–5^, confirming a statistically significant difference between the two distributions. Compounds with *r* < −1 exhibited a distribution skewed toward lower predicted PI values, whereas compounds with *r* ≥ −1 showed a distribution concentrated at higher predicted PI values. These findings indicate that compounds for which the FP uncertainty falls substantially below the threshold are more likely to exhibit low selectivity, suggesting that substantial differences in intermodal uncertainty are associated with reduced selectivity.

Although the uncertainty analysis showed that one modality often exhibits lower uncertainty than the other for certain compounds, using only one modality was insufficient for accurate selectivity prediction. Figure 5(d) compares the RMSE values of the FP-only model, the IMG-only model, and CUE. CUE achieved the lowest RMSE of 0.242, outperforming both the FP-only model (RMSE = 0.245) and the IMG-only model (RMSE = 0.257). These results indicate that integrating the two modalities is beneficial for more accurate selectivity prediction and support the proposed uncertainty-aware fusion approach. The analysis results for DIS-WS2 are provided in Supporting Figure S7.

We further extended this analysis to all benchmark data sets by comparing four modality weighting strategies. FP and IMG assign a weight of 1 to the fingerprint and molecular image modalities, respectively, while assigning a weight of 0 to the other modality. Uniform assigns equal weights to both modalities. Table 2 shows the RMSE comparison of the modality weighting strategies, while detailed performance results across all evaluation metrics are provided in Supporting Tables S5–S6.

**Table 2:** RMSE Performance Comparison of Different Modality Weighting Methods on the DIS and INH Data sets^a^

Data set	Method	DIS	INH
PI	FP	0.245 ± 0.012	0.166 ± 0.007
	IMG	0.257 ± 0.007	0.177 ± 0.005
	Uniform	**0.242** ± **0.010**	0.164 ± 0.003
	CUE	**0.242** ± **0.010**	**0.163** ± **0.004**
Ssel	FP	**0.887** ± **0.042**	0.327 ± 0.043
	IMG	0.957 ± 0.105	0.366 ± 0.038
	Uniform	0.901 ± 0.074	**0.322** ± **0.034**
	CUE	0.895 ± 0.068	**0.322** ± **0.034**
WS2	FP	0.285 ± 0.018	0.214 ± 0.009
	IMG	0.300 ± 0.009	0.230 ± 0.016
	Uniform	**0.282** ± **0.012**	**0.212** ± **0.010**
	CUE	0.282 ± 0.014	**0.212** ± **0.010**
RS2	FP	1.737 ± 0.195	**1.450** ± **0.045**
	IMG	1.806 ± 0.280	1.615 ± 0.060
	Uniform	1.695 ± 0.209	1.455 ± 0.064
	CUE	**1.691** ± **0.188**	1.452 ± 0.062

a Bold values indicate the best performance, and underlined values indicate the second-best performance.

### Effect of Feature Fusion Strategies

In addition to adaptive weighting, the feature fusion strategy also affects the final predictive performance. To investigate this effect, we compared element-wise summation (Sum), early concatenation (Concat (E)), intermediate concatenation (Concat (I)), and the proposed CUE fusion strategy. Illustrations of the architectures for each feature fusion strategy are provided in Supporting Figure S8.

Table 3 shows the average ranks obtained with different fusion strategies. Element-wise summation directly combines information from the two modalities within the same feature space and therefore does not sufficiently capture interactions between them. Early concatenation and intermediate concatenation allow the modality information to be learned jointly, but they apply the same fusion scheme to all samples.

**Table 3:** Average Rank Comparison of Feature Fusion Strategies (Element-Wise Summation (Sum), Early Concatenation (Concat (E)), Intermediate Concatenation (Concat (I)), and CUE)^a^

	RMSE (↓)				PCC (↑)			
Data set	Sum	Concat (E)	Concat (I)	CUE	Sum	Concat (E)	Concat (I)	CUE
DIS-PI	3.0	1.0	4.0	2.0	3.0	1.0	4.0	2.0
DIS-Ssel	4.0	1.0	3.0	2.0	4.0	1.0	3.0	2.0
DIS-WS2	2.0	4.0	3.0	1.0	2.0	4.0	3.0	1.0
DIS-RS2	3.0	4.0	1.0	2.0	3.0	4.0	2.0	1.0
INH-PI	2.0	3.0	4.0	1.0	2.0	3.0	4.0	1.0
INH-Ssel	2.0	4.0	1.0	3.0	2.0	4.0	3.0	1.0
INH-WS2	3.0	4.0	1.0	2.0	3.0	4.0	2.0	1.0
INH-RS2	2.0	4.0	1.0	3.0	3.0	4.0	2.0	1.0
Average	2.6	3.1	2.2	**2.0**	2.8	3.1	2.9	**1.2**

a Bold values indicate the best performance, and underlined values indicate the second-best performance.

In contrast, CUE first adjusts the contribution of each modality according to its uncertainty and then combines the resulting features, allowing it to adapt more flexibly to varying data characteristics. Consequently, CUE achieved the best average rank for most evaluation metrics. Detailed performance results across all benchmarks and evaluation metrics are provided in Supporting Tables S7–S8.

### Hyperparameter Analysis

To analyze the hyperparameter sensitivity of CUE, we evaluated the number of error bins *B* and the number of nearest neighbors *K* used in LTAU, as well as the softmax temperature τ used to calculate the modality weights. Figure 6 shows the RMSE values across the eight benchmarks for different combinations of *B* and *K*. When *B* is too small, prediction errors are concentrated in a limited number of bins, which may prevent sufficiently fine-grained estimation of the empirical error distribution and uncertainty. Increasing *B* generally improved the performance; however, using an excessively large number of bins oversegmented the error distribution and led to performance degradation on some benchmarks. Overall, *B* = 5000 provided the most stable performance and was therefore selected as the default value.

**Figure 6:**
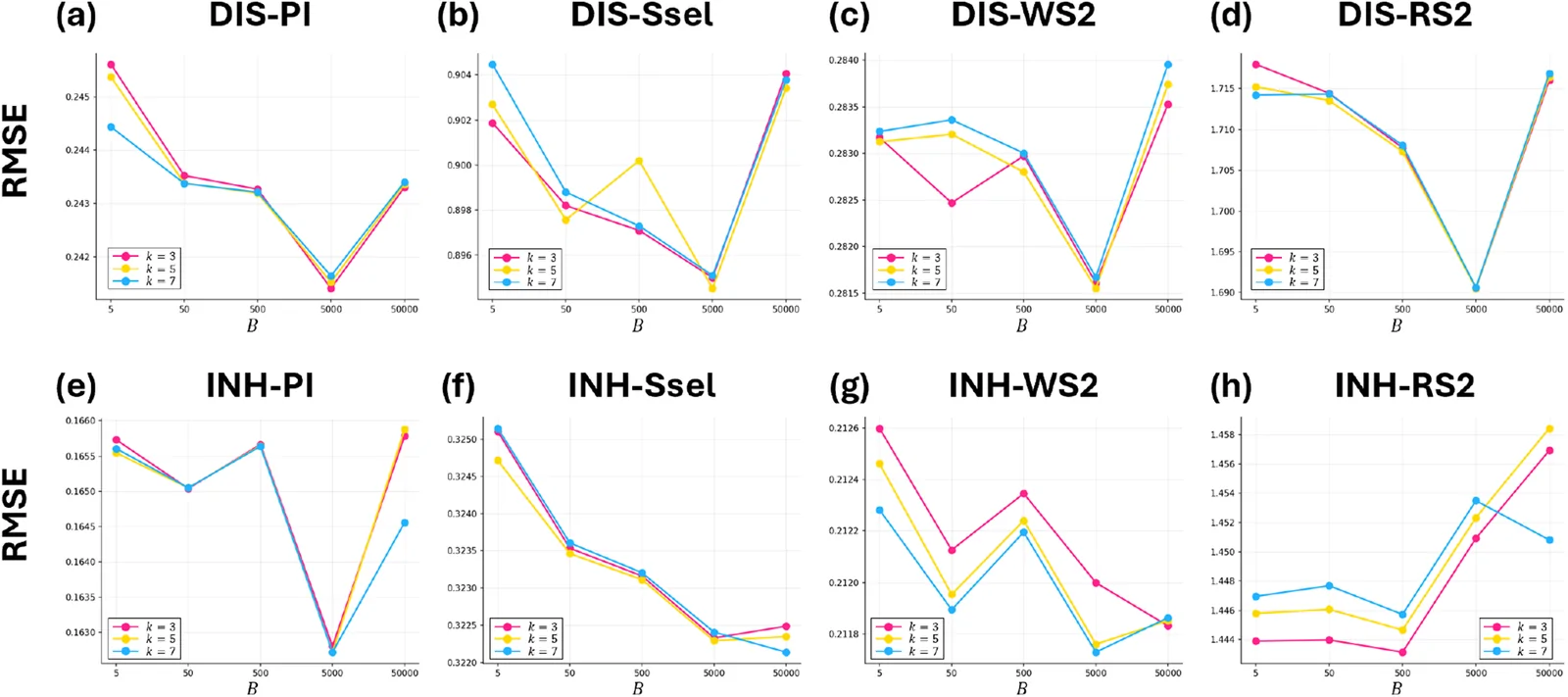
Comparison of RMSE values across different LTAU hyperparameter settings, the number of histogram of bins (*B*) and the number of nearest neighbors (*K*), for selectivity prediction performance. (a–d) DIS benchmarks, (e–h) INH benchmarks.

The performance differences across the evaluated *K* values were small for most benchmarks, with *K* = 3, 5, and 7 showing similar results. This indicates that the performance of CUE is not highly sensitive to *K* within the evaluated range. Accordingly, *K* = 5 was used as the default value. Detailed performance results for all combinations of *B* and *K* are provided in Supporting Tables S9–S10.

We also performed an additional sensitivity analysis of the softmax temperature τ. The differences in predictive performance across the evaluated τ values were generally small, and the ANOVA test showed no statistically significant differences. These results indicate that the performance of CUE is not highly sensitive to τ within the evaluated range. Therefore, τ = 1, a commonly used setting, was selected as the default value. Detailed performance results for each τ value are provided in Supporting Table S11, while the data set-specific *F*-statistics and *p*-values from the ANOVA tests are reported in Supporting Table S12.

### Case Study: Virtual Screening of EGFR­(T790M/C797S) Inhibitors

To demonstrate the practical utility of CUE in targeted therapy applications, we conducted a virtual screening case study targeting EGFR mutants associated with osimertinib resistance. Specifically, we aimed to identify selective candidate compounds that bind to the clinically relevant double mutant T790M/C797S.^54^ The T790M mutation at the gatekeeper position confers resistance to first- and second-generation EGFR inhibitors, and the subsequent emergence of the C797S mutation further abrogates the efficacy of third-generation covalent inhibitors such as osimertinib by preventing covalent bond formation with Cys797. As a benchmark for comparison, we selected compound 19g reported by Lategahn et al.,^54^ which demonstrated potent biochemical activity against the osimertinib-resistant EGFR-T790M/C797S mutant. To our knowledge, compound 19g represents a state-of-the-art inhibitor of this clinically intractable resistance variant.

In this case study, we designed a six-stage hierarchical screening pipeline in which CUE serves as a core component (Figure 7(a)). Each stage progressively narrows the candidate pool from initial molecule generation to the final selection of drug-like candidates.

- **Target-conditional Generation by RxnFlow**: We used RxnFlow^55^ to construct the initial compound pool for EGFR­(T790M/C797S). RxnFlow is a synthesis-oriented generative framework that assembles molecules from approximately 200k building blocks via predefined chemical reaction templates and supports target-conditional generation for structure-based candidate exploration. This step generated 10,000 candidate molecules, which were assigned identifiers from CUE1 to CUE10000.
- **On-Target Filtering by RxnFlow-Proxy**: We used the Proxy model of RxnFlow to evaluate the predicted affinity of both the reference compound 19g and the initial compound pool toward EGFR­(T790M/C797S). Molecules with higher predicted affinity than 19g were retained, resulting in approximately 6,750 candidate molecules.
- **Mutation-aware Filtering by HMM-DTA**: HMM-DTA was employed to predict binding affinities against EGFR variants including EGFR wild-type, EGFR­(T790M), and EGFR­(C797S). Molecules exhibiting higher predicted affinity toward any other EGFR variant than toward EGFR­(T790M/C797S) were excluded, retaining compounds predicted to preferentially bind to the on-target EGFR­(T790M/C797S) mutant. This step yielded 64 candidate molecules.
- **Selectivity Filtering by CUE**: CUE was used as the central selectivity prediction module to prioritize molecules with selective kinase binding profiles. The remaining candidate molecules were evaluated using the CUE model trained on the DIS-PI benchmark. We used the PI as the selectivity metric to estimate whether each molecule showed a target-focused binding profile across kinases. Molecules with predicted PI values greater than 0.5 were retained, thereby filtering candidates based on predicted kinase selectivity rather than on-target affinity alone. This step yielded 45 candidate molecules.
- **Drug-likeness Filtering by Lipinski**’**s Rule**: Lipinski’s rule of five is a widely used criterion for evaluating oral drug-likeness based on molecular weight, log *P*, hydrogen bond donors, and hydrogen bond acceptors. We applied Lipinski’s rule using the thresholds of molecular weight <500 Da, log *P* < 5, hydrogen bond donors ≤ 5, and hydrogen bond acceptors ≤ 10. This step retained 39 candidate molecules.
- **Synthetic Accessibility Filtering by RDKit**: Synthetic accessibility was assessed using the Synthetic Accessibility Score (SAScore),^56^ calculated with RDKit. Lower SAScore values indicate greater predicted synthetic accessibility. Molecules with SAScore values lower than 4 were retained, resulting in 13 final candidate compounds (Figure 7(b) and Supporting Table S13).

**Figure 7:**
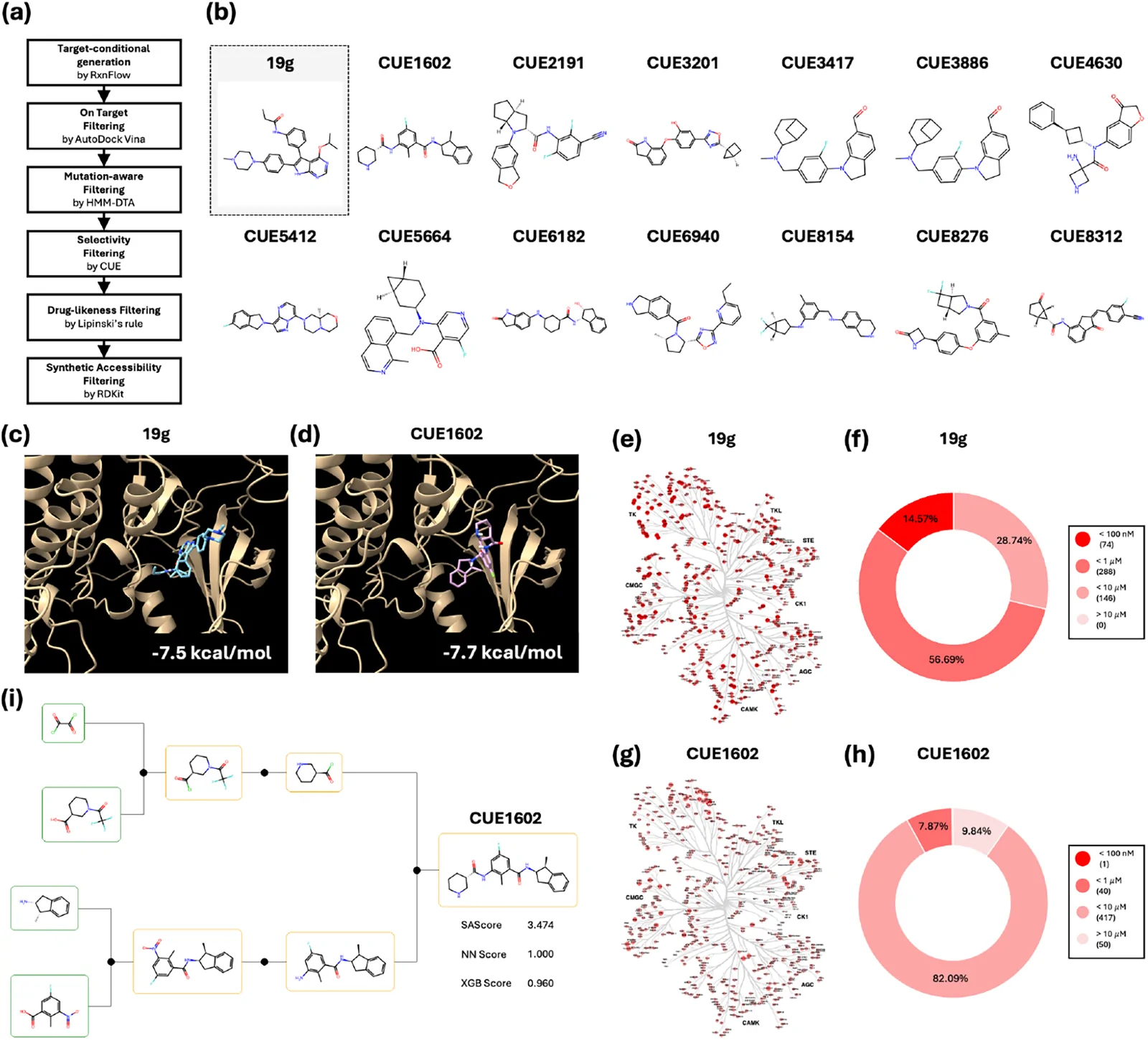
Target-selective drug screening using CUE1602. (a) Workflow of the screening process. (b) Chemical structures of the selected candidate molecules. (c, d) 3D visualizations of EGFR­(T790M/C797S)–ligand complexes for the reference compound 19g and CUE1602, generated using AutoDock Vina and visualized with ChimeraX. (e, g) kinome maps of the reference compound 19g and the CUE-selected compound CUE1602. (f, h) Distributions of predicted kinase binding affinities across predefined affinity ranges for 19g and CUE1602. (i) Retrosynthesis analysis of candidate CUE1602: predicted retrosynthetic route generated by AiZynthFinder, with SAScore and RAScore­(NN Score, XGB Score).

To evaluate the final 13 molecules, we performed molecular docking simulations using AutoDock Vina^57^ and reported their predicted binding affinities in Figure 7 and Supporting Figure S9. The resulting protein–ligand binding poses were visualized using ChimeraX.^58^ Figure 7(c),(d) show representative docking results for the reference compound 19g and the candidate molecule CUE1602, respectively. The docking simulations yielded predicted binding affinities of −7.5 kcal/mol for 19g and −7.7 kcal/mol for CUE1602.

To visualize the kinase binding profiles of the candidate molecules, kinome maps were generated using CORAL^59^ based on affinity values predicted by HMM-DTA across 508 kinases. Figure 7(e),(g) present the kinome maps for 19g and CUE1602, respectively. To complement these visualizations, Figure 7(f),(h) show the distributions of predicted kinase binding affinities across predefined affinity ranges for 19g and CUE1602, respectively. For 19g, 14.57% (74 kinases) and 56.69% (288 kinases) of the 508 kinases exhibited predicted affinities of <100 nM and <1 μM, respectively, indicating a broad kinase binding profile. In contrast, CUE1602 showed reduced proportions of 0.20% (1 kinase) at <100 nM and 7.87% (40 kinases) at <1 μM, reflecting a narrower kinase binding profile. These results suggest that CUE1602 has reduced predicted interactions with potential off-target kinases while retaining high predicted affinity for EGFR­(T790M/C797S). The predicted kinase affinity distributions for the remaining candidate molecules, excluding CUE1602, are provided in Supporting Figure S10.

To further assess the synthetic feasibility of CUE1602, retrosynthesis planning was performed using AiZynthFinder,^60^ and the resulting synthetic route is presented in Figure 7(i). Synthetic accessibility was additionally evaluated using the SAScore and the Retrosynthetic Accessibility score (RAScore).^61^ CUE1602 achieved an SAScore of 3.474, an NN-based RAScore of 1.000, and an XGB-based RAScore of 0.960, indicating favorable predicted synthetic accessibility. The retrosynthesis planning results for the other candidate molecules are provided in Supporting Figure S11.

Overall, these results suggest that the CUE-based screening pipeline can identify candidate molecules with high kinase selectivity by jointly accounting for on-target affinity and off-target selectivity.

## Conclusion

In this study, we proposed CUE, an uncertainty-aware multimodal molecular feature extraction framework for drug selectivity prediction. CUE extracts modality-specific embeddings from molecular fingerprints and 2D molecular structure images, and integrates them through LTAU-based uncertainty-weighted summation to predict drug selectivity.

Experimental results demonstrate that CUE consistently outperforms DTA-based models and QSAR-based models across all eight benchmarks in the DIS and INH groups, achieving the lowest RMSE on every data set. UMAP visualization further indicates that CUE embeddings effectively encode selectivity-related information in the learned representation space. Analysis of the uncertainty-based modality weighting reveals that the relative magnitude of uncertainty between modalities correlates with drug selectivity, and that single-modality predictions underperform the integrated approach, supporting the value of uncertainty-aware adaptive weighting for effective multimodal integration.

In a case study targeting the EGFR­(T790M/C797S) mutant, the CUE-based screening pipeline demonstrated its utility in identifying candidate molecules with desired binding affinity and selectivity profiles. In particular, the selected candidates exhibited improved selectivity profiles compared to the reference compound.

Although the proposed framework is applicable to diverse molecular modalities, this study focused on molecular fingerprints and 2D molecular structure images. Among the single modalities we evaluated (Supporting Tables S14–S16), molecular fingerprints achieved the best overall single-modality performance and were adopted as the primary representation, and 2D molecular structure images, which were among the strongest of the remaining single modalities, were incorporated as a complementary representation.

This uncertainty-aware fusion strategy is not limited to drug selectivity prediction. In broader QSAR tasks, modality-specific uncertainty may vary depending on the compound and the target property. This suggests that LTAU-based adaptive weighting could serve as a general strategy for constructing reliable molecular embeddings in multimodal QSAR modeling.

In future work, we plan to synthesize the final candidates identified in the case study and validate their activity and selectivity through in vitro experiments in collaboration with researchers in chemistry and medicinal chemistry. Beyond this experimental validation, and building on the two modalities used in this study, we will extend CUE to integrate three or more molecular modalities and develop it into a general multimodal QSAR framework for broader drug property prediction tasks, including ADMET, bioactivity, and toxicity prediction. We will also advance the uncertainty estimation itself by exploring alternative uncertainty quantification methods, such as ensemble- and Monte Carlo-dropout-based approaches, to further improve the reliability and interpretability of uncertainty-aware modality fusion.

## Supplementary Material



## Data Availability

Our data and source code are available at https://github.com/jjjabcd/CUE.

## Notes

Detailed data collection, preprocessing, and cross-validation procedures, including the formal definitions of the selectivity metrics (partition index, selectivity entropy, window score, and ranking score) and the parameter settings used for WS and RS (Section A1); illustration of experimental pipelines for selectivity prediction (Figure S1); schematic illustration of the data split strategy for cross-validation under a drug cold-start scenario (Figure S2); distributions of the four selectivity metrics (PI, Ssel, WS2, and RS2) computed from the DIS data set, derived from ChEMBL, and from the DAVIS data set (Figure S3); illustration of embedding extraction points used for embedding analysis across different models (Figure S4); UMAP visualization of molecular embeddings for additional benchmarks on the DIS data set (Figure S5); UMAP visualization of molecular embeddings for benchmarks on the INH data set (Figure S6); analysis of multimodal uncertainty and selectivity prediction on the DIS-WS2 data set (Figure S7); schematic illustration of the feature fusion strategies compared in the ablation study (Figure S8); docking simulation analysis of EGFR­(T790M/C797S)-ligand complexes for the CUE-selected inhibitors (Figure S9); distribution of predicted kinase binding affinities across CUE-selected compounds (Figure S10); retrosynthesis analysis of CUE-selected candidates predicted by AiZynthFinder, with SAScore and RAScore (Figure S11); distributions of continuous selectivity scores across DIS and INH data sets (Figure S12); number of samples in the selectivity data set (compound-level) and the DTA data set (compound–kinase pair-level) used in 5-fold cross-validation (Table S1); summary of baseline models used for selectivity prediction performance comparison, including model category, input representation, and official implementation repository (Table S2); predictive performance of CUE and the baseline models on the four DIS benchmarks, evaluated by RMSE, *R* ^2^, and PCC (Table S3); predictive performance of CUE and the baseline models on the four INH benchmarks, evaluated by RMSE, *R* ^2^, and PCC (Table S4); comparison of fingerprint-only (FP), molecular image-only (IMG), equal-weighting (uniform), and CUE models on the DIS data set via 5-fold cross-validation (Table S5); comparison of fingerprint-only (FP), molecular image-only (IMG), equal-weighting (Uniform), and CUE models on the INH data set via 5-fold cross-validation (Table S6); comparison of feature fusion strategies (element-wise summation, early concatenation, intermediate concatenation, and CUE) on the DIS data set via 5-fold cross-validation (Table S7); comparison of feature fusion strategies (element-wise summation, early concatenation, intermediate concatenation, and CUE) on the INH data set via 5-fold cross-validation (Table S8); performance of CUE across different LTAU hyperparameter settings, the number of histogram bins (*B*) and the number of nearest neighbors (*K*), on the DIS data set via 5-fold cross-validation (Table S9); performance of CUE across different LTAU hyperparameter settings, the number of histogram bins (*B*) and the number of nearest neighbors (*K*), on the INH data set via 5-fold cross-validation (Table S10); performance of CUE across different values of the uncertainty-weighting temperature parameter τ on the DIS and INH benchmark data sets via 5-fold cross-validation (Table S11); statistical significance test (*F*-statistic and *p*-value) of performance variation across hyperparameter settings for each DIS and INH benchmark (Table S12); summary of compounds identified through virtual screening for EGFR­(T790M/C797S)-selective inhibitors (Table S13); performance comparison across single-modalities on DIS data set (Table S14); performance comparison across single-modalities on INH data set (Table S15), and average rank comparison of selectivity prediction across single-modalities (Table S16) (PDF)

## Abbreviations

DTA: drug–target affinity
QSAR: quantitative structure–activity relationship
CUE: chemical uncertainty-aware embedding
LTAU: loss trajectory analysis for uncertainty
OCSR: optical chemical structure recognition
ECFP: extended-connectivity fingerprint
UQ: uncertainty quantification
EGFR: epidermal growth factor receptor
PI: partition index
Ssel: selectivity entropy
WS: window score
RS: ranking score
SMILES: simplified molecular-input line-entry system
MLP: multilayer perceptron
RMSE: root mean squared error
PCC: Pearson’s correlation coefficient
UMAP: uniform manifold approximation and projection
