# Supplementary material for: CUE: A Chemical Uncertainty-Aware Embedding Framework for Multimodal Drug Selectivity Prediction
Source: J Chem Inf Model. 2026 Sep 17;66(19):12402–16. doi: 10.1021/acs.jcim.6c01761 (PMC13663480; doi:10.1021/acs.jcim.6c01761)
Supplement: Supplementary file 1 [file ci6c01761_si_001.pdf]

Supporting Information

CUE: A Chemical Uncertainty-aware Embedding Framework for Multi-modal Drug Selectivity Prediction

Jin Hyuk Kim,[1] Gyeong Hwan Kim,[1] Hyeon Jun Park,[1] and Jonghwan Choi[1,*]

[1]Department of Computer Engineering, Hallym University, 1 Hallymdaehak-gil, Chuncheon-si, Gangwon-do 24252, Republic of Korea

[*]Corresponding author: jonghwanc@hallym.ac.kr

Table of Contents

A1 Supplementary Methods **5**

A1.1 Dataset Collection 5
A1.2 Dataset Preprocessing 5
A1.3 Data Split for Cross-Validation 8
A1.4 Dataset Statistics 8

A2 Supplementary Figures **9**

S1 Illustration of experimental pipelines for selectivity prediction. 9
S2 Schematic illustration of the data split strategy for cross-validation under a drug cold-start scenario. 10
S3 Distributions of the four selectivity metrics (PI, Ssel, WS2, and RS2) computed from the DIS dataset, derived from ChEMBL, and from the DAVIS dataset. 11
S4 Illustration of embedding extraction points used for embedding analysis across different models. 12
S5 UMAP visualization of molecular embeddings for additional benchmarks on the DIS dataset. 13
S6 UMAP visualization of molecular embeddings for benchmarks on the INH dataset. 14
S7 Analysis of multi-modal uncertainty and selectivity prediction on the DIS-WS2 dataset. 15
S8 Schematic illustration of the feature fusion strategies compared in the ablation study. Element-wise summation (Sum), Early concatenation (Concat(E)), Intermediate concatenation (Concat(I)), and CUE. 16
S9 Docking simulation analysis of candidates: 3D visualizations of EGFR(T790M/C797S)-ligand complexes for the 12 CUE-selected inhibitors, generated using AutoDock Vina and visualized with ChimeraX, with corresponding docking scores (kcal/mol) indicated for each compound. 17
S10 Distribution of predicted kinase binding affinities across CUE-selected compounds. 18

S11 Retrosynthesis analysis of CUE-selected candidates: predicted retrosynthetic route generated by AiZynthFinder, with SAScore and RAScore(NN Score, XGB Score). 19
S12 Distributions of continuous selectivity scores across DIS and INH datasets 23

A3 Supplementary Tables 24

S1 Number of samples in the selectivity dataset (compound-level) and the DTA dataset (compound–kinase pair-level) used in five-fold cross-validation. 24
S2 Summary of baseline models used for selectivity prediction performance comparison, including model category, input representation, and official implementation repository. . . . 25
S3 Predictive performance of CUE and the baseline models on the four DIS benchmarks (DIS-PI, DIS-Ssel, DIS-WS2, and DIS-RS2), evaluated by RMSE, R^2, and PCC. 26
S4 Predictive performance of CUE and the baseline models on the four INH benchmarks (INH-PI, INH-Ssel, INH-WS2, and INH-RS2), evaluated by RMSE, R^2, and PCC. 27
S5 Comparison of fingerprint-only (FP), molecular image-only (IMG), equal-weighting (Uniform), and CUE models on the DIS dataset via 5-fold cross-validation. 28
S6 Comparison of fingerprint-only (FP), molecular image-only (IMG), equal-weighting (Uniform), and CUE models on the INH dataset via 5-fold cross-validation. 29
S7 Comparison of feature fusion strategies (Element-wise summation (Sum), Early concatenation (Concat(E)), Intermediate concatenation (Concat(I)), and CUE) on the DIS dataset via 5-fold cross-validation. 30
S8 Comparison of feature fusion strategies (Element-wise summation (Sum), Early concatenation (Concat(E)), Intermediate concatenation (Concat(I)), and CUE) on the INH dataset via 5-fold cross-validation. 31
S9 Performance of CUE across different LTAU hyperparameter settings, the number of histogram bins (B) and the number of nearest neighbors (K), on the DIS dataset via 5-fold cross-validation. 32

S10 Performance of CUE across different LTAU hyperparameter settings, the number of histogram bins (B) and the number of nearest neighbors (K), on the INH dataset via 5-fold cross-validation. 34
S11 Performance of CUE across different values of the uncertainty-weighting temperature parameter τ on the DIS and INH benchmark datasets via 5-fold cross-validation. 36
S12 Statistical significance test (F-statistic and p-value) of performance variation across hyperparameter settings for each DIS and INH benchmark. 38
S13 Summary of compounds identified through virtual screening for EGFR(T790M/C797S)-selective inhibitors 39
S14 Performance comparison across single-modalities on DIS dataset 40
S15 Performance comparison across single-modalities on INH dataset 41
S16 Average rank comparison of selectivity prediction across single modalities 42

A1 Supplementary Methods

In this study, we constructed datasets for drug selectivity prediction by collecting large-scale compound–kinase affinity data and subsequently deriving selectivity datasets. Since selectivity is defined at the compound level, pair-level affinity data were reorganized into compound-level kinase binding profiles. In addition, because the CUE and QSAR baseline models[1,2] use data formats different from those of DTA baseline models[3–6], we jointly designed data preprocessing and cross-validation strategies to enable fair performance comparisons.

A1.1 Dataset Collection

The primary data sources containing compound, kinase, and affinity information were obtained from the ChEMBL v35 database[7]. To restrict the analysis to human kinases, we collected a list of 555 *Homo sapiens* kinases from the KLIFS database[8] and used this list to filter kinase targets in the ChEMBL dataset. As a result, only compound–kinase affinity data associated with human kinases were retained. K_d and K_i affinity measurements were collected and processed independently, resulting in a K_d affinity dataset of 2,062 compounds associated with up to 485 kinases, and a K_i affinity dataset of 7,804 compounds associated with up to 267 kinases.

A1.2 Dataset Preprocessing

Using the human kinase list obtained from the KLIFS database, we further filtered the ChEMBL dataset to retain only targets corresponding to human kinases. Although ChEMBL provides mutation annotations for kinases, these mutations are not directly reflected in the corresponding FASTA sequences. To address this issue, we applied mutation annotations for amino acid substitutions, deletions, and insertions to wild-type FASTA sequences and reconstructed sequence-level representations.

During the mutation application process, entries in which the mutation position exceeded 1,000 residues relative to the FASTA sequence length were excluded, as such cases exceed the maximum input length of the DTA models and are effectively treated as identical to wild-type sequences. In addition, kinase entries corresponding to recombinant constructs used in kinase profiling

experiments were excluded, as they hinder direct comparison with wild-type kinases and may compromise the consistency of selectivity profiles.

For compound–kinase pairs with multiple reported affinity values, the values were averaged to obtain a single representative affinity. Based on the resulting refined compound–kinase affinity dataset, compound-level kinase binding profiles were constructed.

Drug selectivity was computed using the distribution of affinity values for each compound across multiple kinases. To remove entries that do not satisfy the definition of selectivity, compounds associated with affinity information for fewer than two kinases were excluded from the dataset. This filtering step ensured that each compound retained in the dataset had affinity values for at least two kinases. After applying all preprocessing steps, the K_d-based selectivity dataset comprised 556 compounds (**DIS**), and the K_i-based selectivity dataset comprised 1,824 compounds (**INH**).

Notation for Selectivity Metrics. Before introducing each metric, we define the notations used throughout the formulations. Let x denote a compound and D denote a panel of kinases. The function $f_{BA}(x, y)$ represents the experimentally measured binding affinity (K_d or K_i) of compound x against kinase $y \in D$. To avoid numerical instability arising from extremely small affinity values, we introduce a lower-bounded affinity function:

$$g_{BA}(x, y) = \max\{f_{BA}(x, y),\ \varepsilon\} \tag{1}$$

where $\varepsilon > 0$ is a small positive constant that serves as a lower bound on the binding affinity. In this study, we set $\varepsilon = 10^{-9}$. The kinase-specific binding probability of compound x across the panel D is defined as:

$$h_{BA}(x, y) = \frac{1/g_{BA}(x, y)}{\sum_{z \in D} 1/g_{BA}(x, z)} \tag{2}$$

The resulting value represents the relative binding probability of compound x to kinase y among all kinases in D. Based on these notations, the four selectivity metrics used in this study are defined as follows.

Partition Index (PI): The Partition Index estimates the maximum fraction of compound binding assigned to the most potently inhibited kinase relative to the remaining kinases in the panel. It is defined as the maximum binding probability across the kinase panel:

$$P(x) = \max_{y \in D} h_{BA}(x, y) \tag{3}$$

A value close to 1 indicates that the binding profile is dominated by one kinase, suggesting that the compound preferentially binds to a specific kinase in the panel. When several kinases show comparable binding affinity, the partitioning of the compound is distributed across multiple targets, resulting in a lower PI value.

Selectivity Entropy (Ssel): Selectivity Entropy is calculated from the entropy of the binding probability distribution $h_{BA}(x, y)$ across the kinase panel:

$$Ssel(x) = -\sum_{y \in D} h_{BA}(x, y) \log h_{BA}(x, y) \tag{4}$$

A lower entropy value indicates that the compound binding is concentrated on a smaller number of kinases, corresponding to a more selective profile. Because Selectivity Entropy is derived from an affinity-based thermodynamic formulation, it accounts for the distribution of binding affinities across targets rather than relying solely on rank ordering.

Window Score (WS): The Window Score is calculated by counting the kinases whose affinity values fall within a predefined activity window from the strongest binding affinity:

$$W(x; \alpha) = \frac{|\{y \in D \mid f_{BA}(x, y) \leq \alpha \cdot \min_{z \in D} f_{BA}(x, z)\}|}{|D|} \tag{5}$$

A lower WS value indicates that fewer kinases are included in the defined binding window, suggesting that the compound preferentially binds to a limited number of kinases. In our experiments, the window parameter α was fixed to 2.

Ranking Score (RS): The Ranking Score is defined as the log-ratio between the k-th smallest binding affinity and the minimum binding affinity across the kinase panel:

$$R(x; k) = \ln \left(\frac{\overset{k}{\min\limits_{y \in D}} f_{BA}(x, y)}{\min\limits_{z \in D} f_{BA}(x, z)} \right) \tag{6}$$

where $\min_{y \in D}^{k} f_{BA}(x, y)$ denotes the affinity value of the k-th ranked kinase after sorting the affinity values in ascending order. A higher RS value reflects a larger activity gap between the most strongly bound kinase and the k-th ranked kinase, indicating a more selective compound. In our experiments, the rank parameter k was fixed to 2.

A1.3 Data Split for Cross-Validation

To evaluate selectivity prediction performance under realistic drug discovery settings, we designed a cross-validation strategy that considers a drug cold-start scenario. Because the selectivity dataset is defined at the compound level, compounds included in the test set were strictly excluded from the training process of the DTA baseline models.

Specifically, compounds were first split into training and test sets at the compound level. Compound–kinase pair data corresponding to test set compounds were then extracted and used as the test set for DTA baseline models. The validation set was randomly sampled from the training set, comprising 10% of the training compounds. An overview of the data splitting strategy is illustrated in Supplementary Figure S2.

This splitting strategy prevents information leakage regarding test compounds and enables a more rigorous performance comparison between the selectivity prediction models and the DTA baseline models.

A1.4 Dataset Statistics

The distributions of continuous selectivity scores for both datasets are presented in Supplementary Figure S12. Supplementary Table S1 summarizes the number of samples in the training, validation, and test sets across five folds for both the selectivity dataset (compound-level) and the DTA dataset (compound–kinase pair-level).

A2 Supplementary Figures

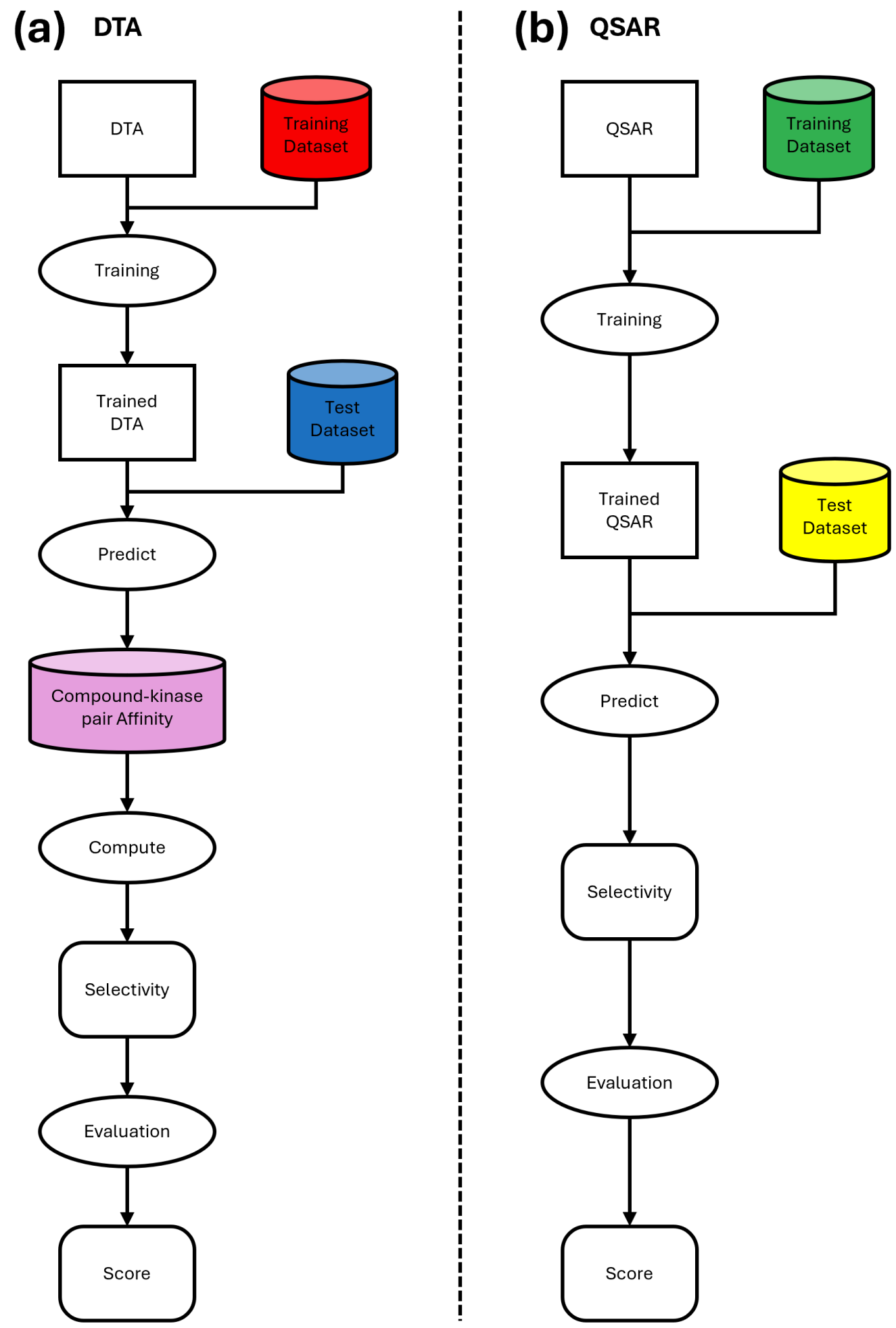

Supplementary Figure S1: Illustration of experimental pipelines for selectivity prediction. (a) DTA-based pipeline, where compound–kinase affinities are predicted and subsequently used to compute selectivity metrics. (b) QSAR-based pipeline, where selectivity is directly predicted from molecular representations.

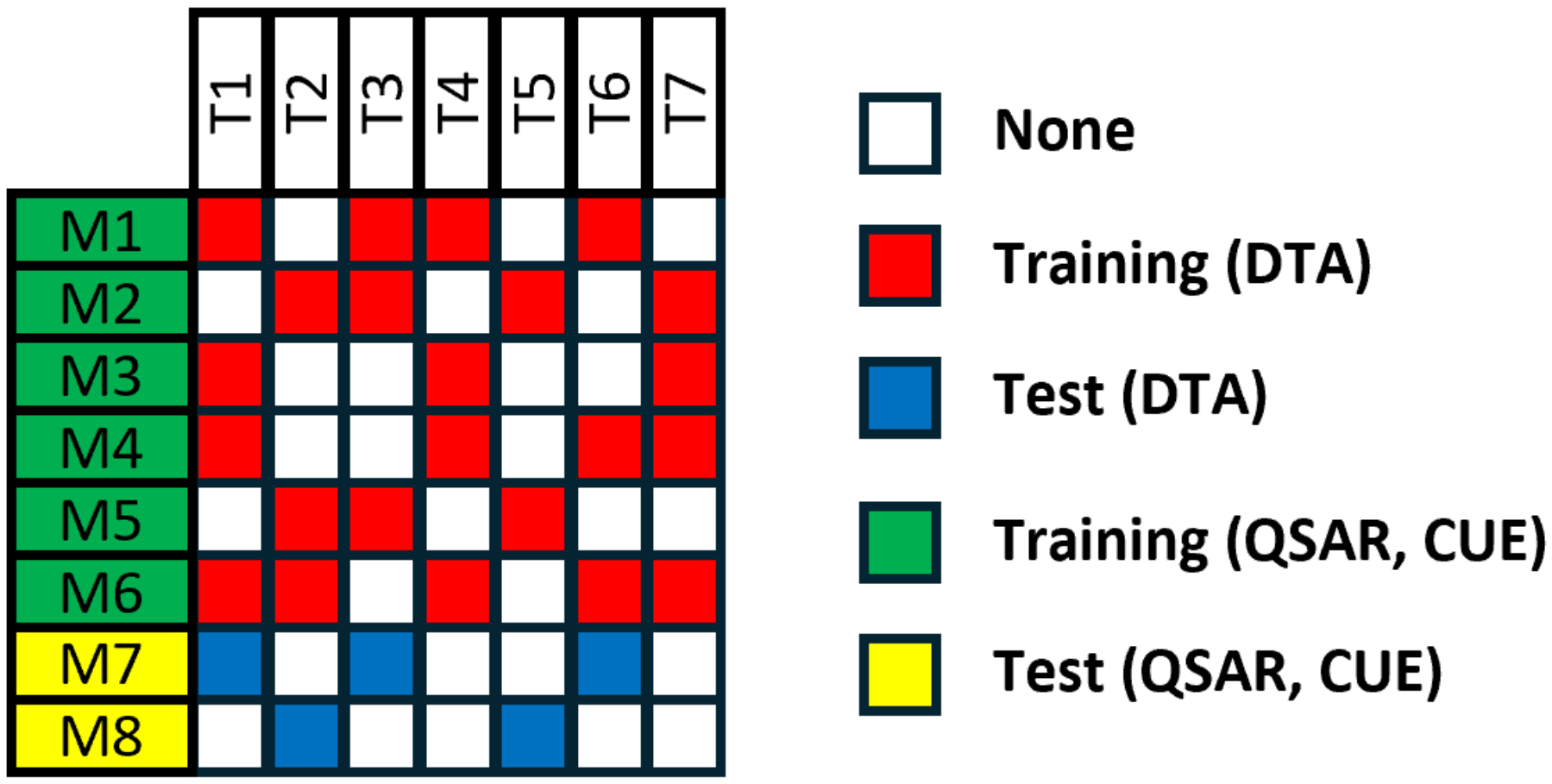

Supplementary Figure S2: Schematic illustration of the data split strategy for cross-validation under a drug cold-start scenario. Rows represent compounds (M1–M8), and columns represent kinases (T1–T7). Green and yellow blocks indicate compounds used for training and testing in the selectivity prediction task, respectively. Red and blue blocks denote compound–kinase pairs used for training and testing in DTA baseline models, respectively. White blocks indicate compound–kinase pairs for which no experimental binding affinity values are available. Compounds included in the selectivity test set are strictly excluded from the DTA training set to prevent information leakage.

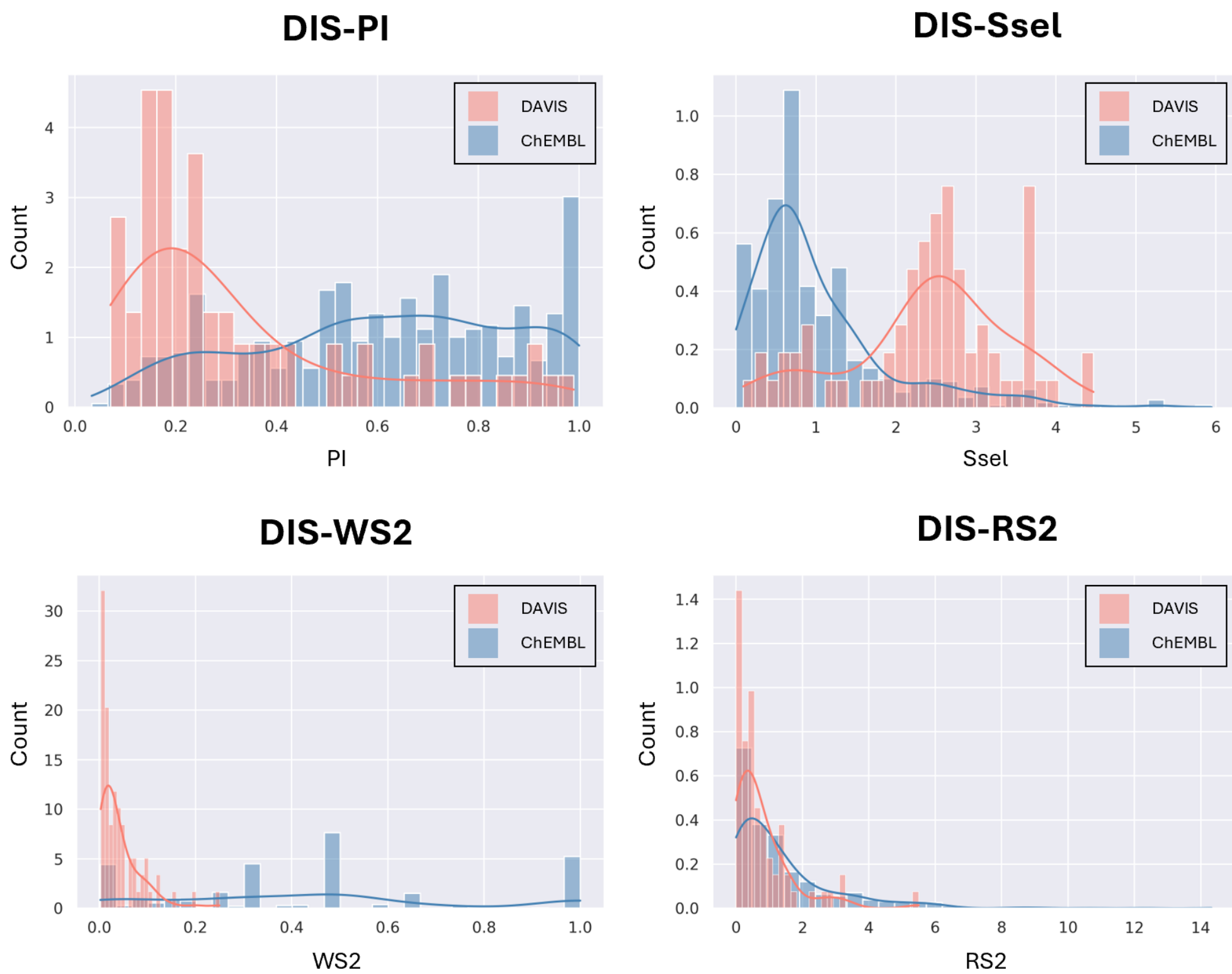

Supplementary Figure S3: Distributions of the four selectivity metrics (PI, Ssel, WS2, and RS2) computed from the DIS dataset, derived from ChEMBL, and from the DAVIS dataset. The distributions of PI, Ssel, and WS2 differ substantially between the two datasets, whereas those of RS2 largely overlap, reflecting that RS2 is computed from the relative ranking of kinase affinities rather than from their absolute values. Densities are shown for both datasets to account for the difference in the number of compounds.

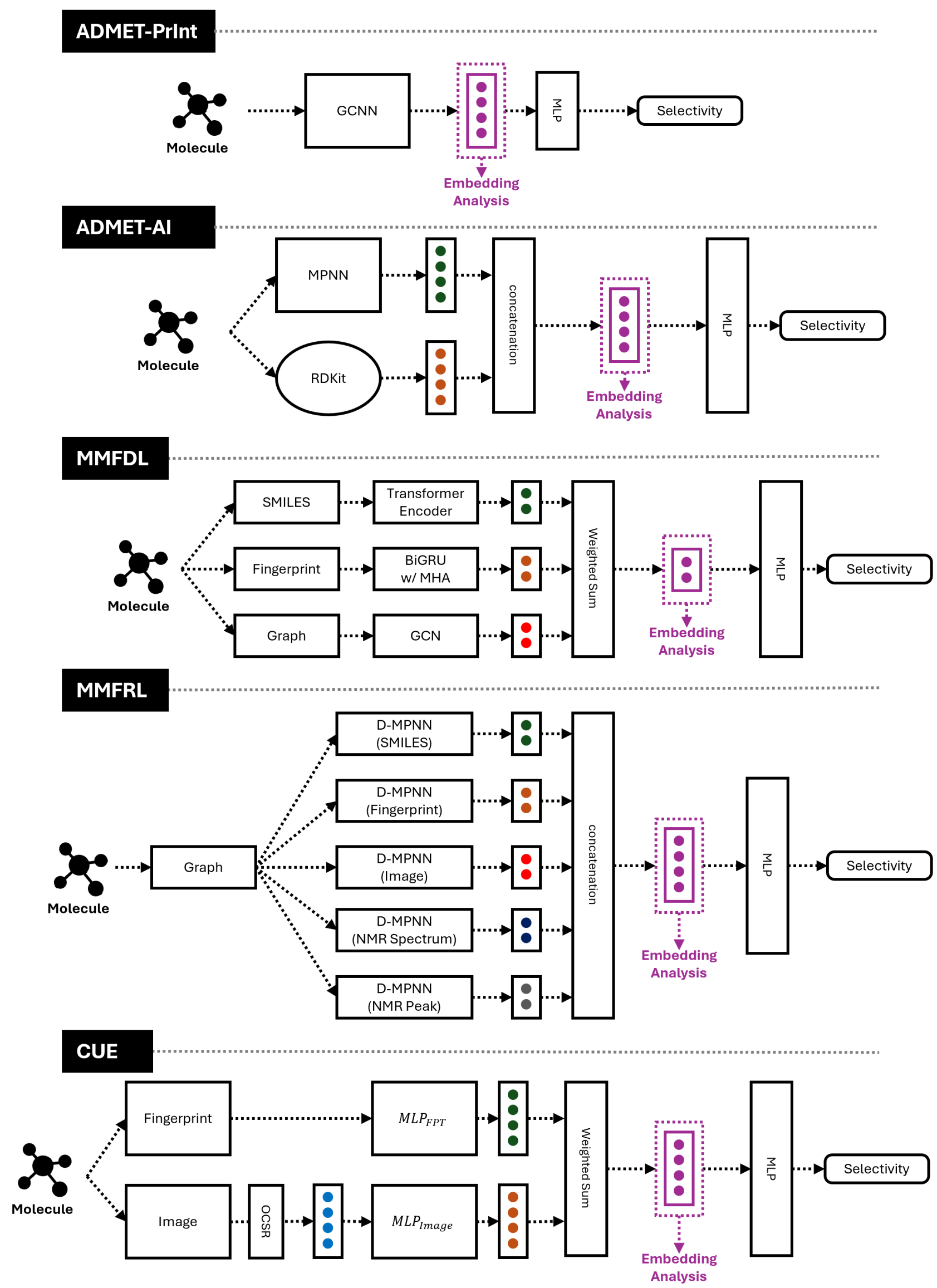

Supplementary Figure S4: Illustration of embedding extraction points used for embedding analysis across different models. The figure highlights where molecular embeddings are extracted from ADMET-PrInt, ADMET-AI, MMFDL, MMFRL, and CUE architectures.

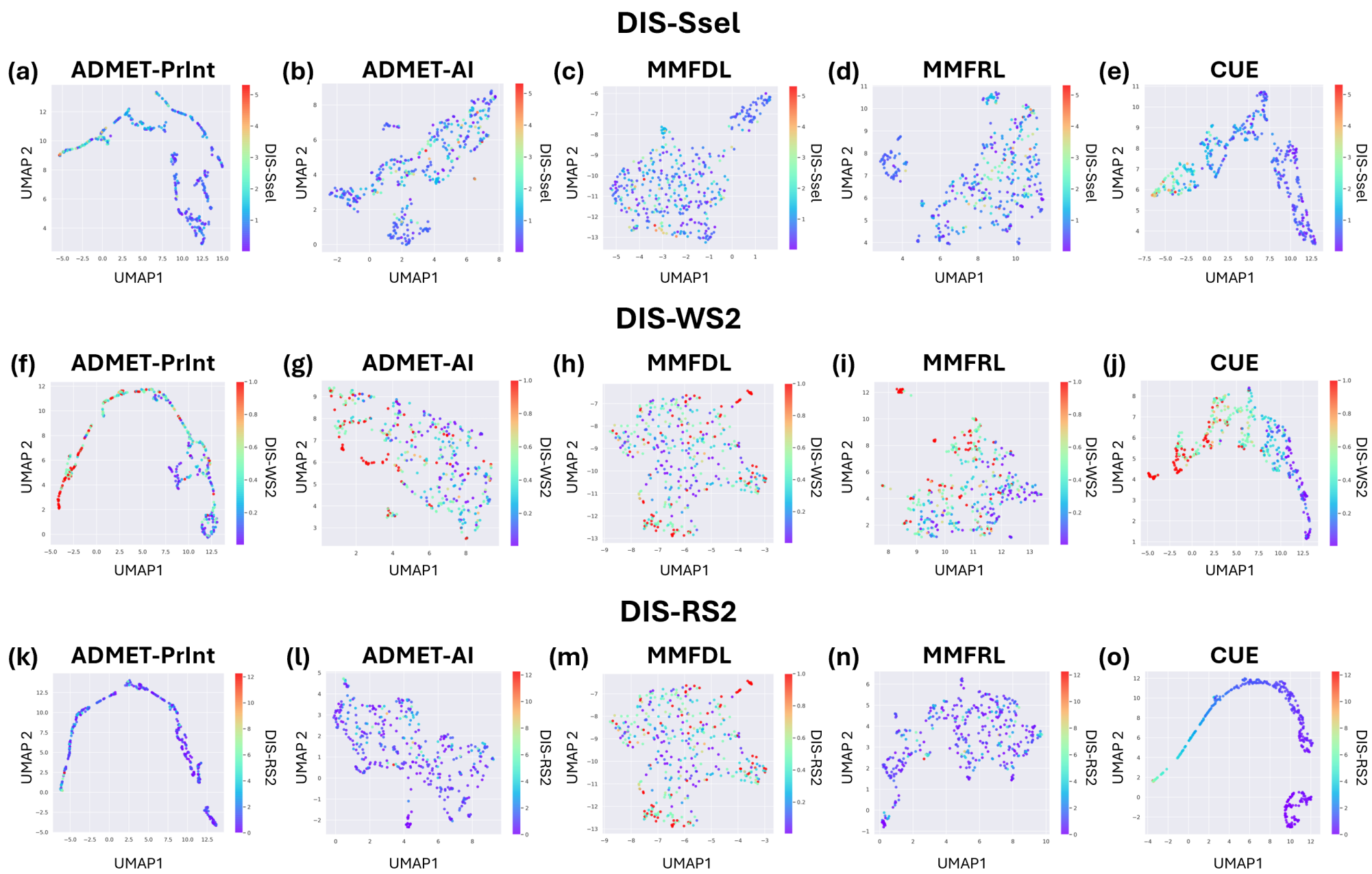

Supplementary Figure S5: UMAP visualization of molecular embeddings for additional benchmarks on the DIS dataset. (a–c) DIS-Ssel, (d–f) DIS-WS2, (g–i) DIS-RS2. Each point represents a molecule, and colors indicate the corresponding selectivity scores.

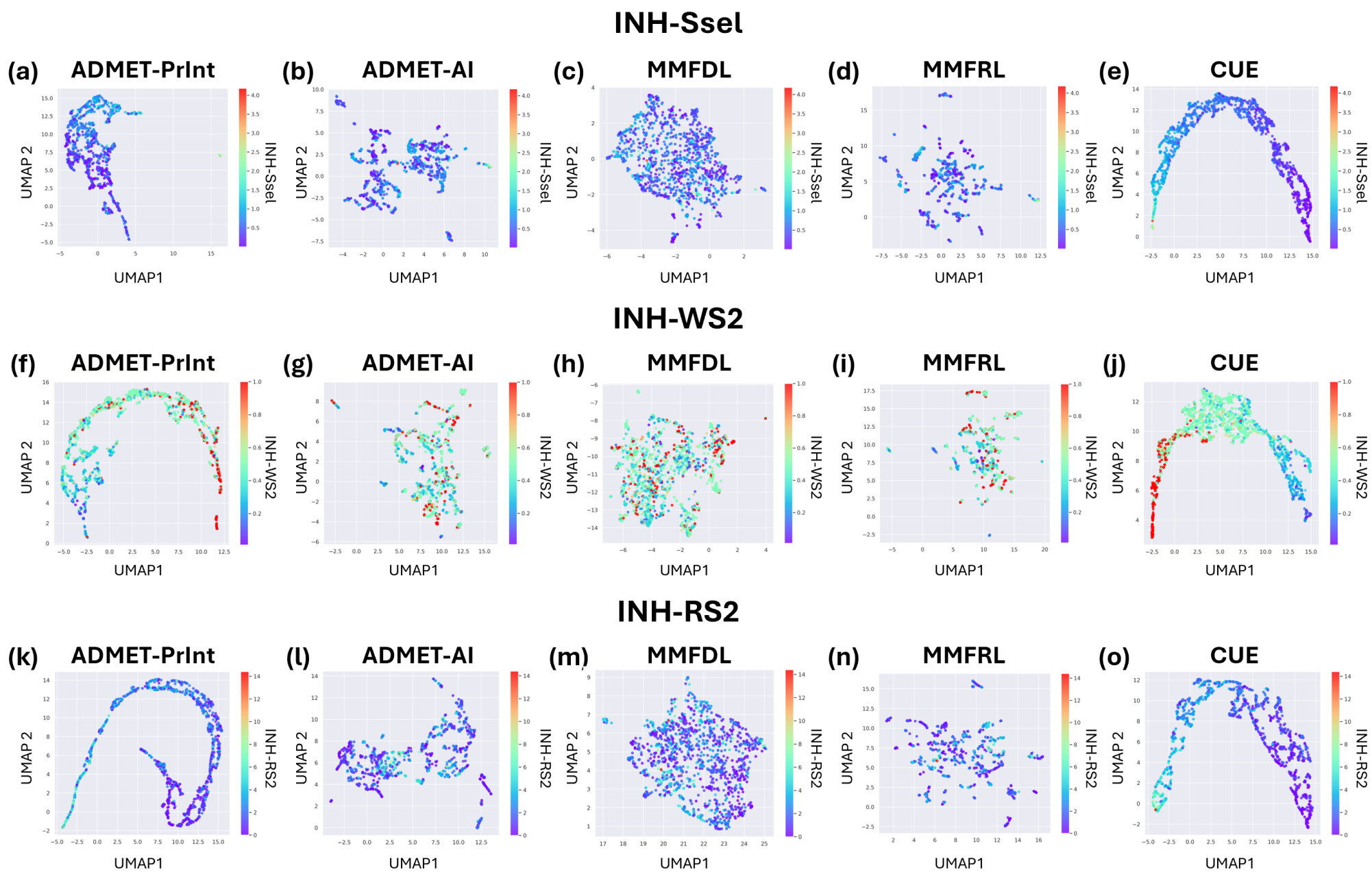

Supplementary Figure S6: UMAP visualization of molecular embeddings for benchmarks on the INH dataset. (a–c) INH-Ssel, (d–f) INH-WS2, (g–i) INH-RS2. Each point represents a molecule, and colors indicate the corresponding selectivity scores.

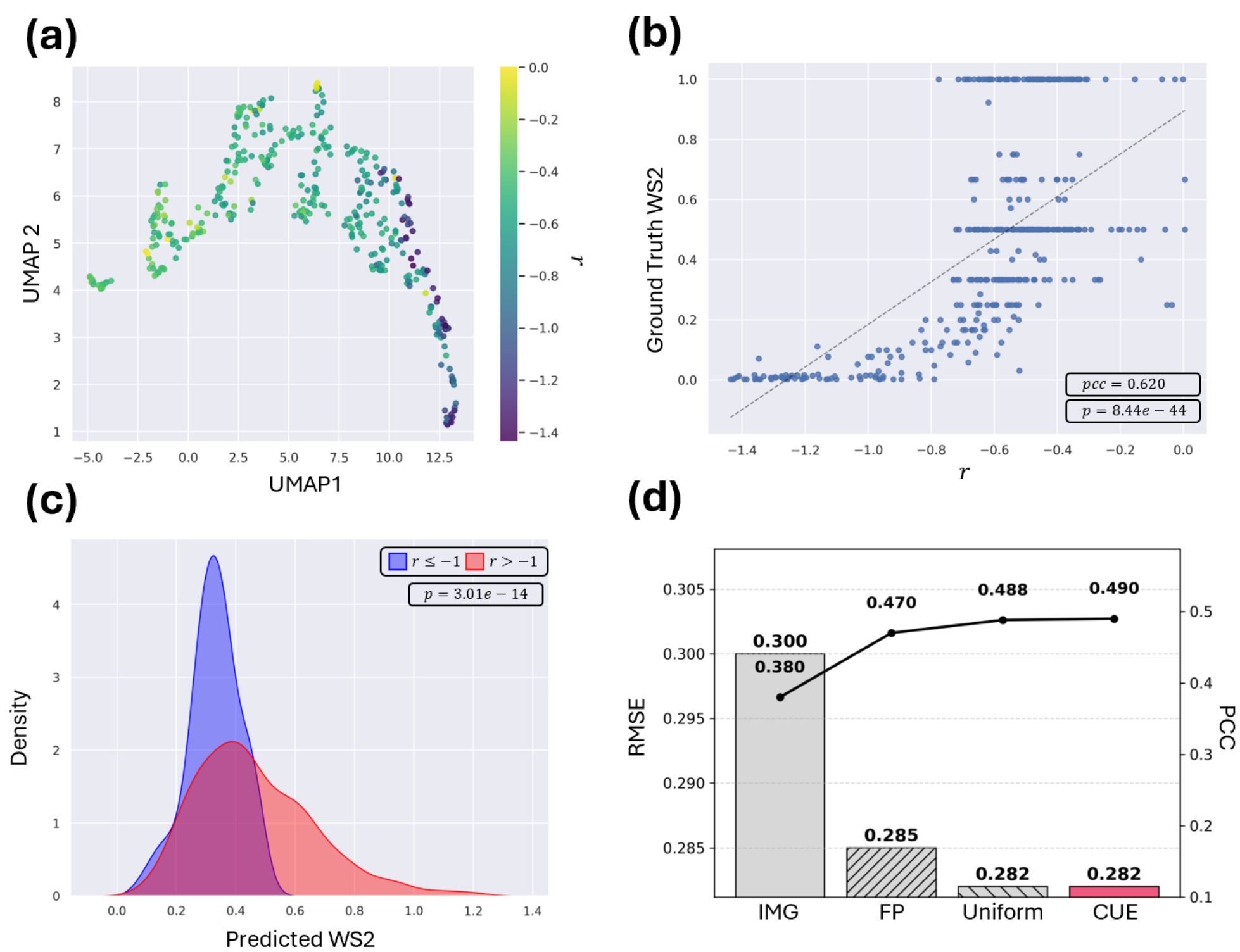

Supplementary Figure S7: Analysis of multi-modal uncertainty and selectivity prediction on the DIS-WS2 dataset. (a) UMAP visualization of compound embeddings colored by the uncertainty ratio r. (b) Pearson correlation analysis between the uncertainty ratio and ground-truth WS2 values. (c) Kernel Density Estimation (KDE) of predicted WS2 values grouped by the uncertainty ratio threshold $r = -1$. (d) RMSE and PCC comparison across molecular image-only (IMG) fingerprint-only (FP), equal-weighting (Uniform), and CUE models.

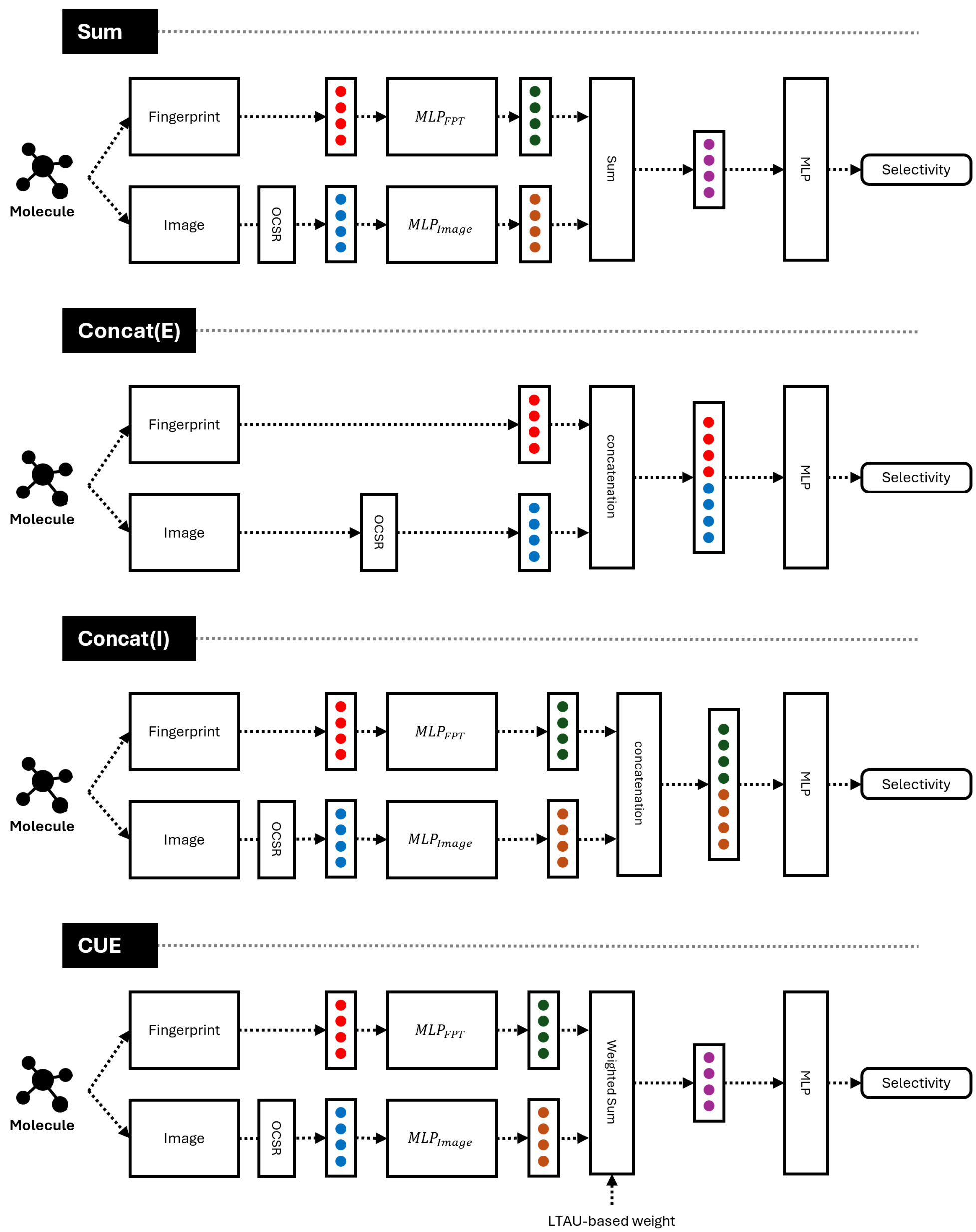

Supplementary Figure S8: Schematic illustration of the feature fusion strategies compared in the ablation study. Element-wise summation (Sum), Early concatenation (Concat(E)), Intermediate concatenation (Concat(I)), and CUE.

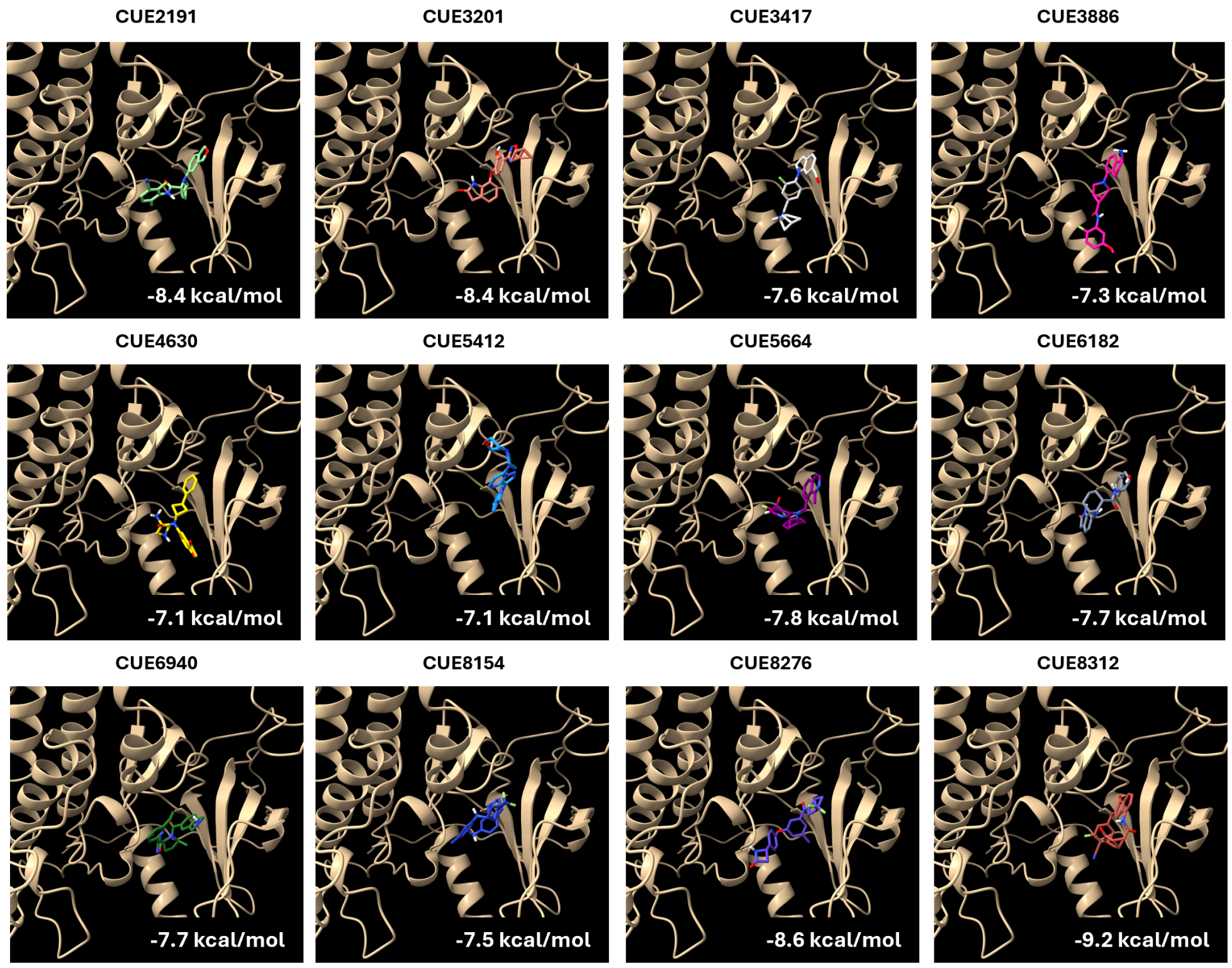

Supplementary Figure S9: Docking simulation analysis of candidates: 3D visualizations of EGFR(T790M/C797S)-ligand complexes for the 12 CUE-selected inhibitors, generated using AutoDock Vina and visualized with ChimeraX, with corresponding docking scores (kcal/mol) indicated for each compound.

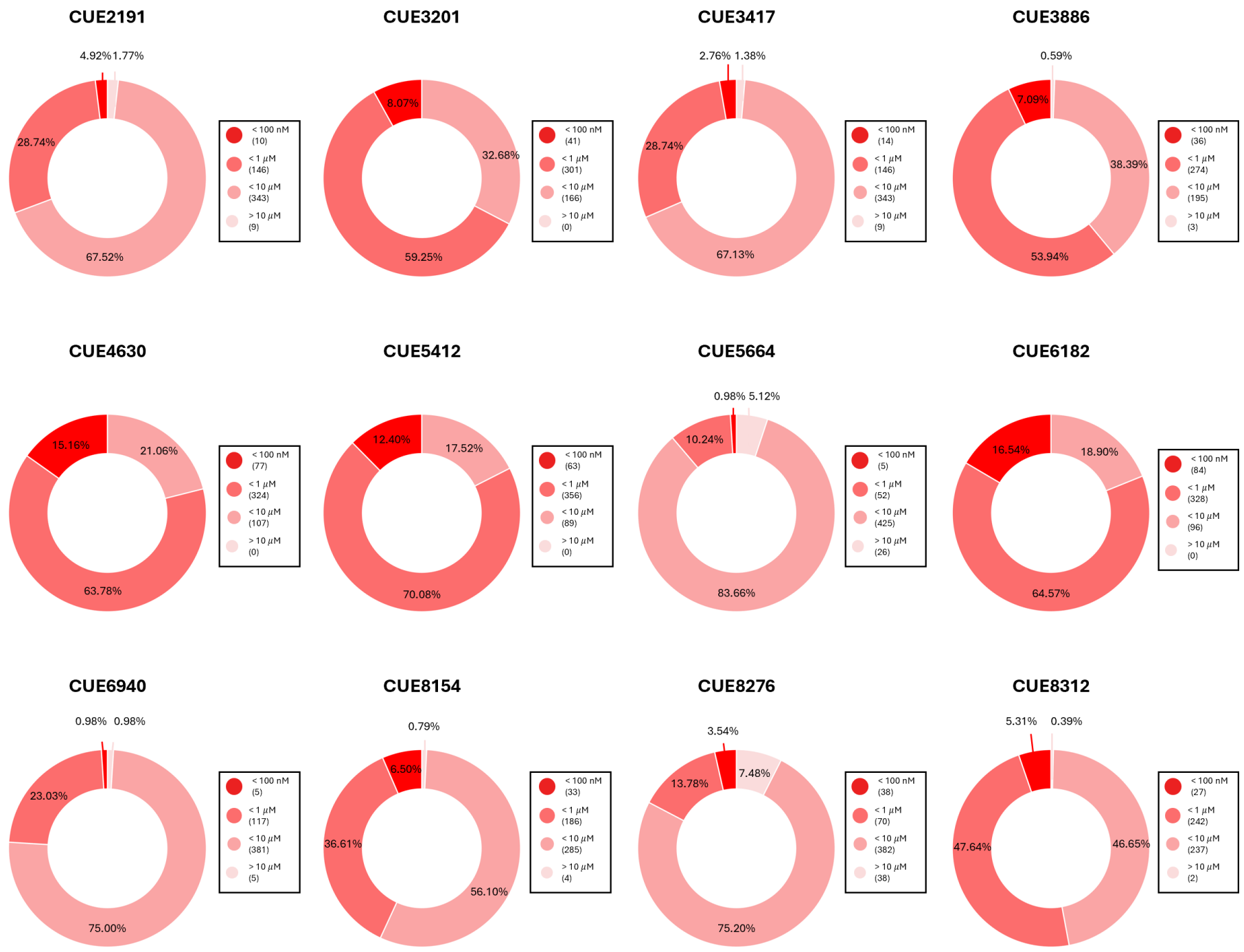

Supplementary Figure S10: Distribution of predicted kinase binding affinities across CUE-selected compounds.

CUE2191

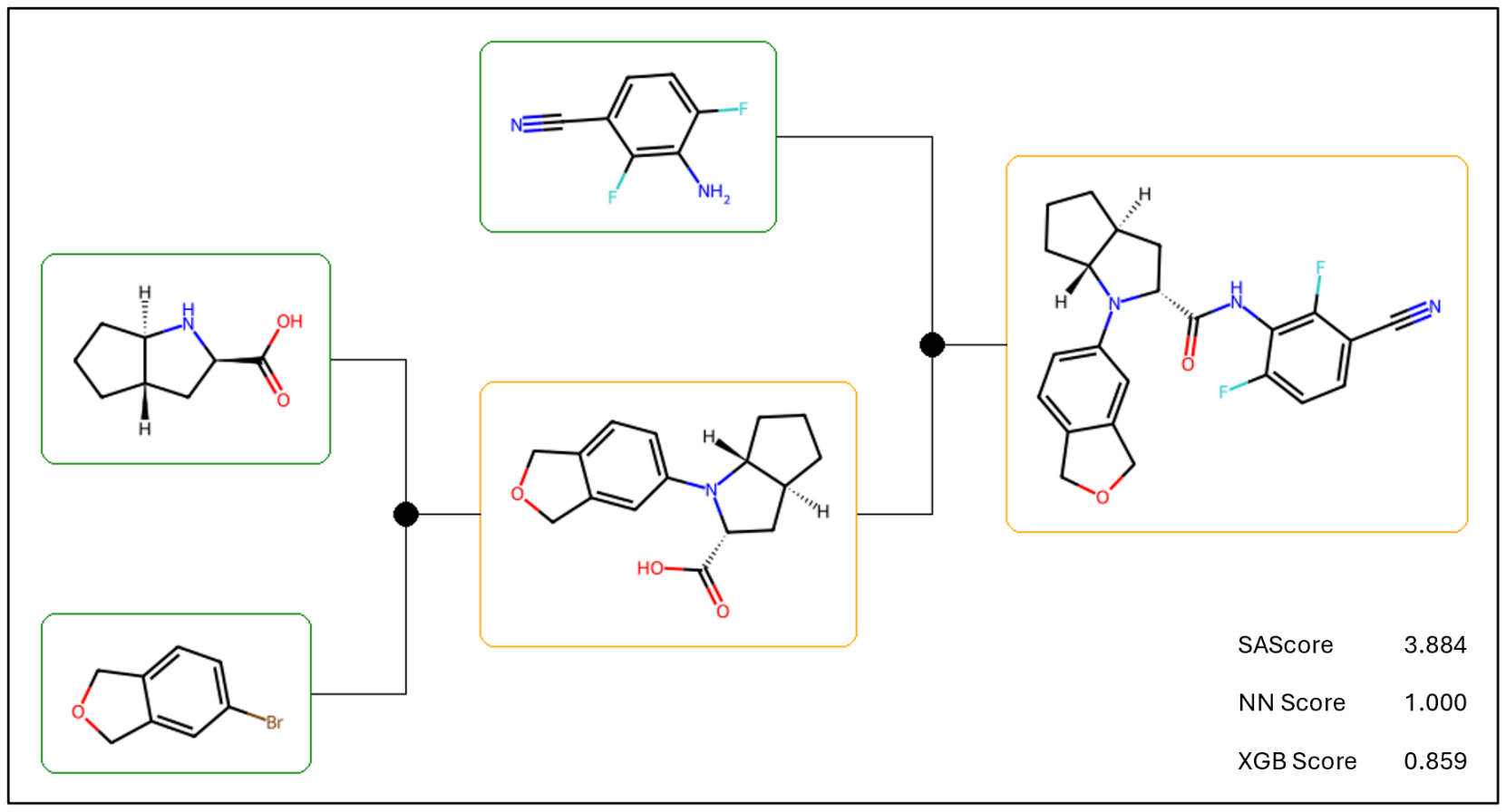

CUE3201

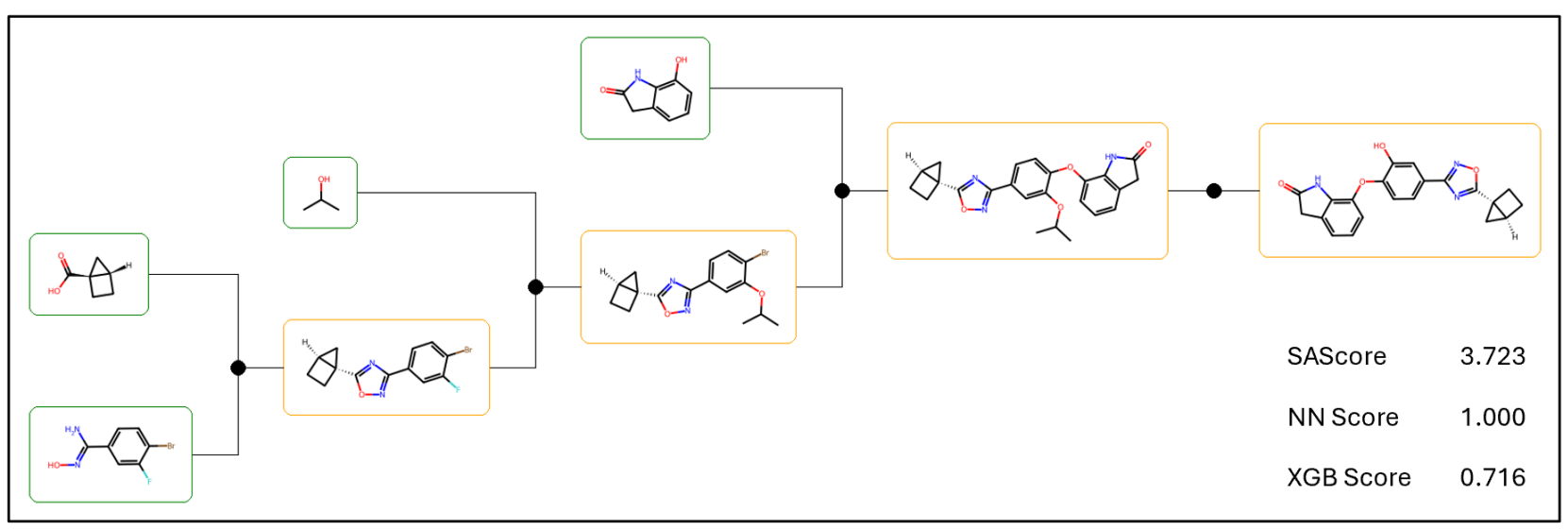

CUE3417

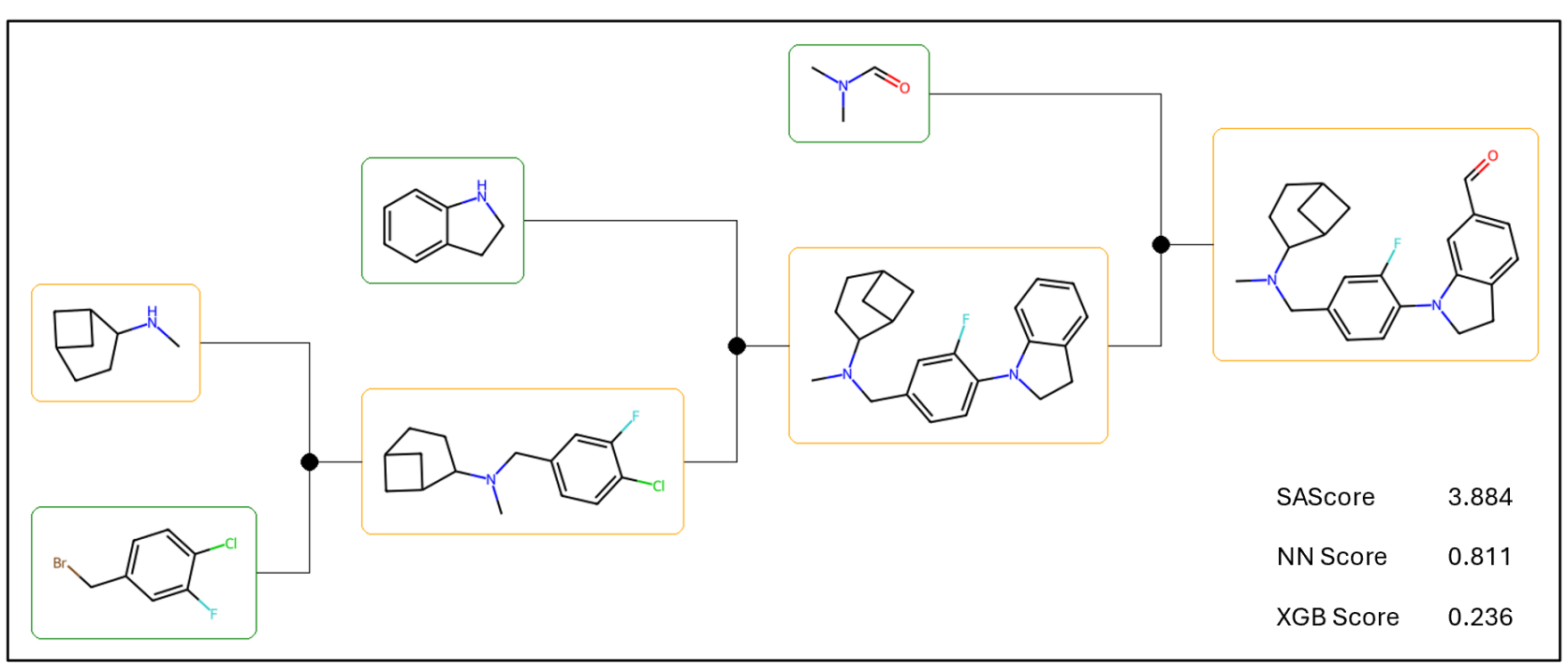

Supplementary Figure S11: Retrosynthesis analysis of CUE-selected candidates: predicted retrosynthetic route generated by AiZynthFinder, with SAScore and RAScore(NN Score, XGB Score).

CUE3886

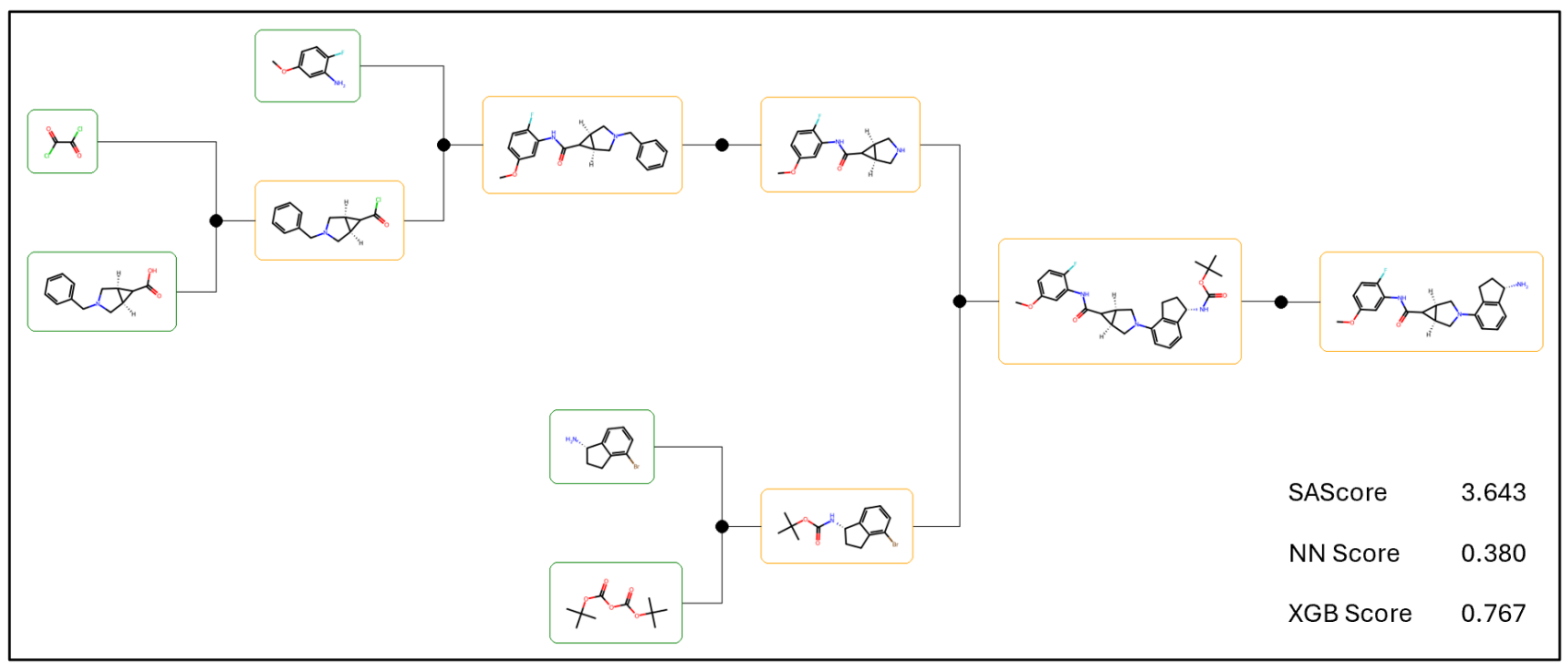

CUE4630

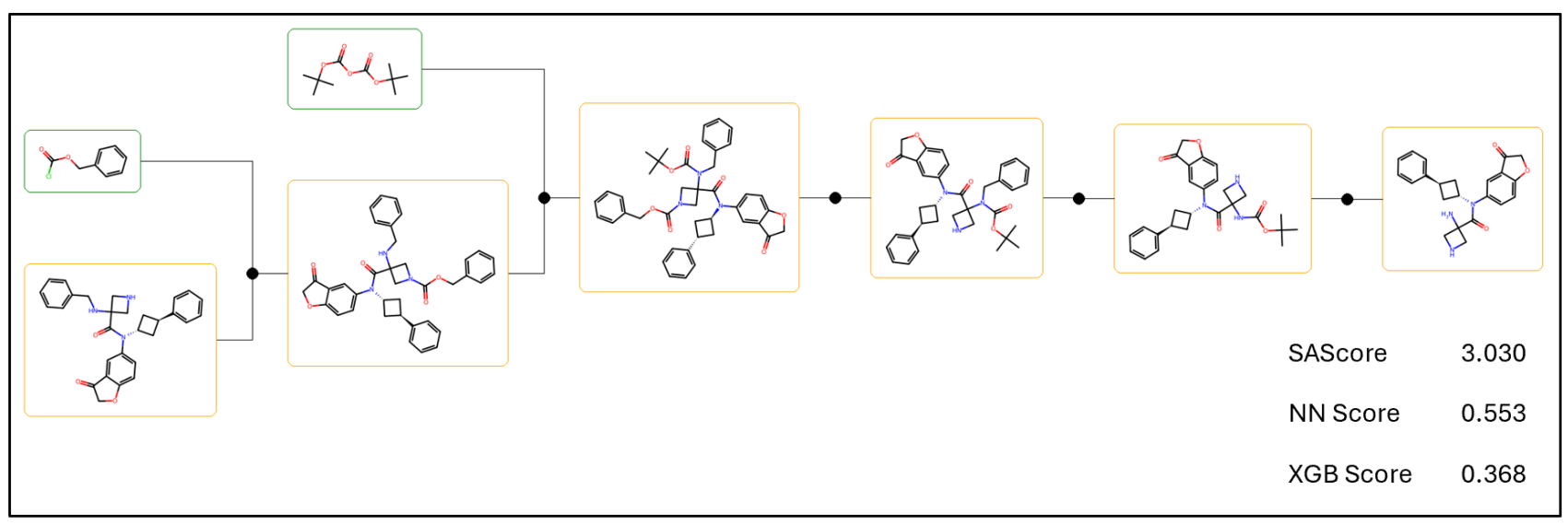

CUE5412

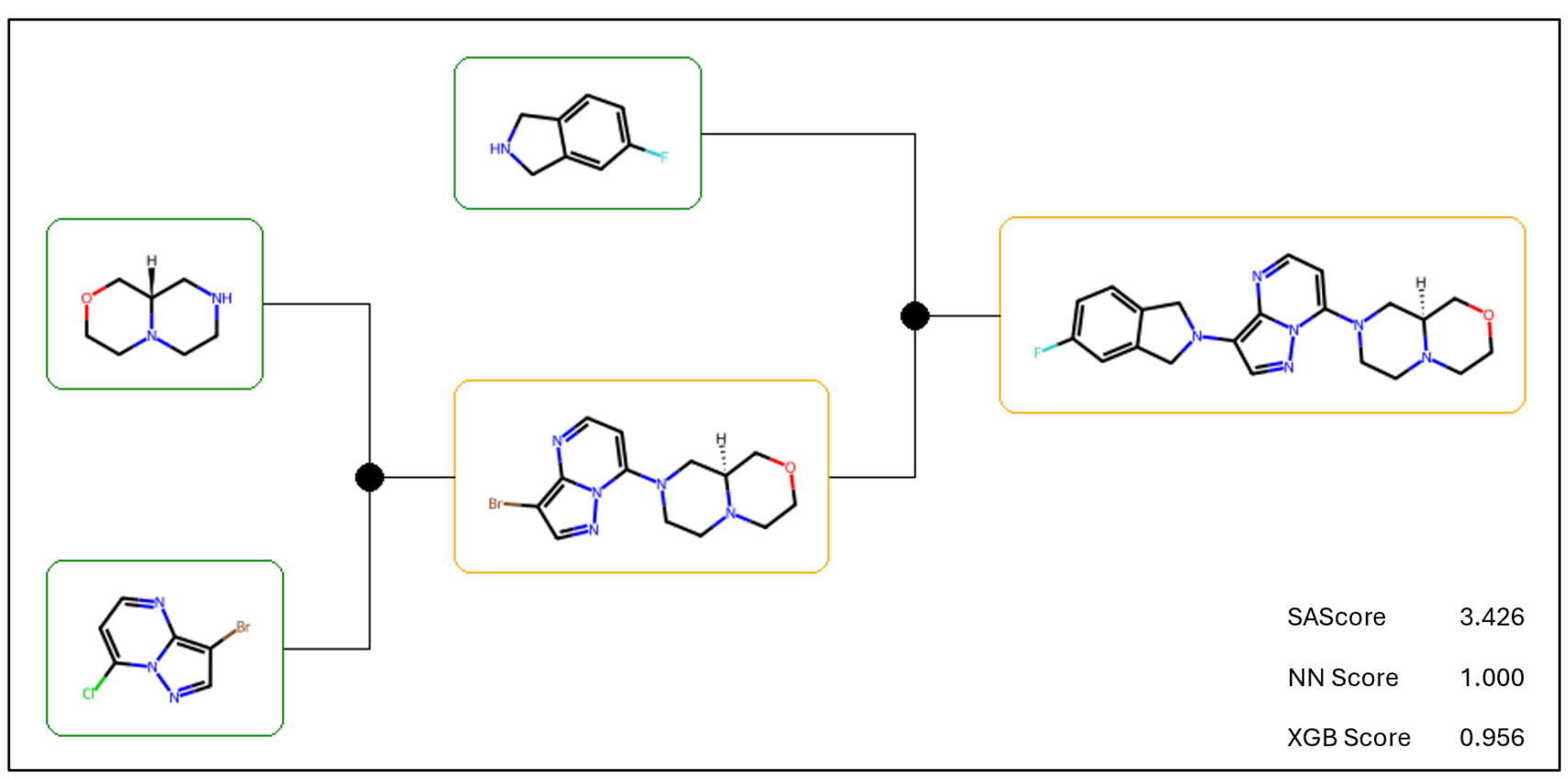

Supplementary Figure S11(Cont.): Retrosynthesis analysis of CUE-selected candidates: predicted retrosynthetic route generated by AiZynthFinder, with SAScore and RAScore(NN Score, XGB Score).

CUE5664

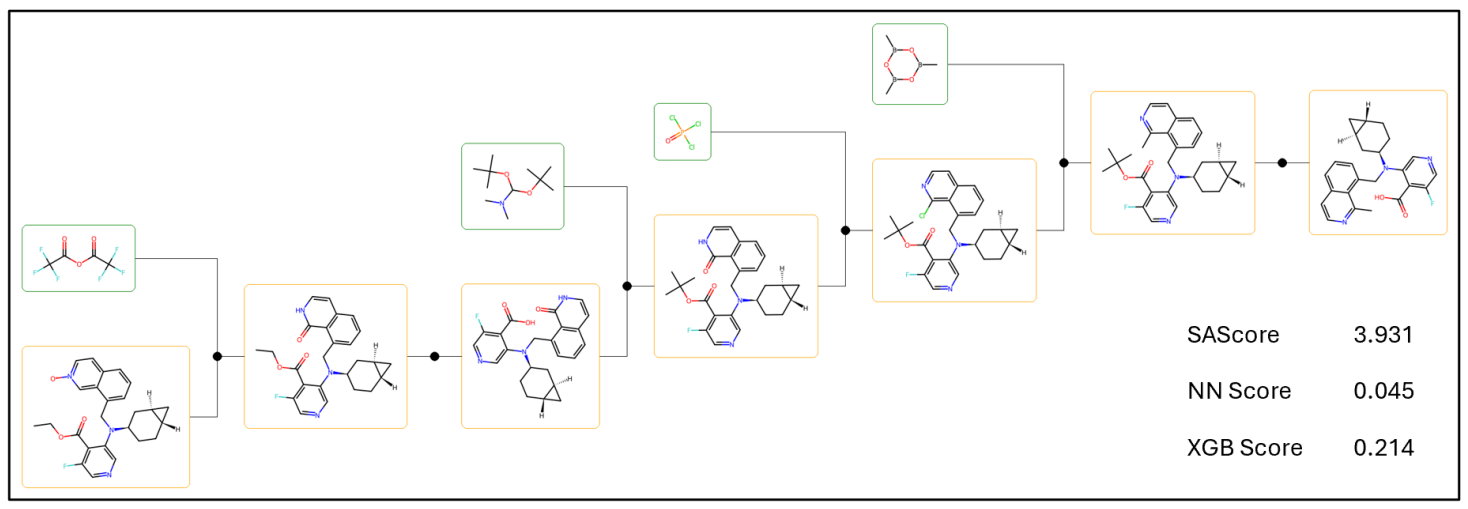

CUE6182

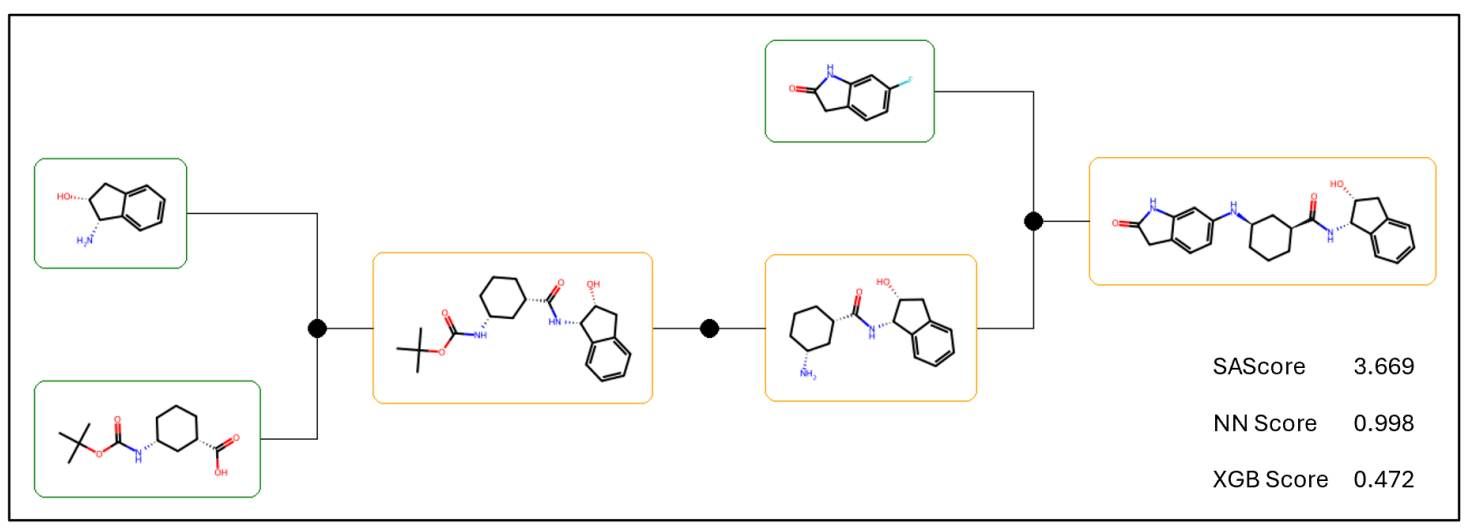

CUE6940

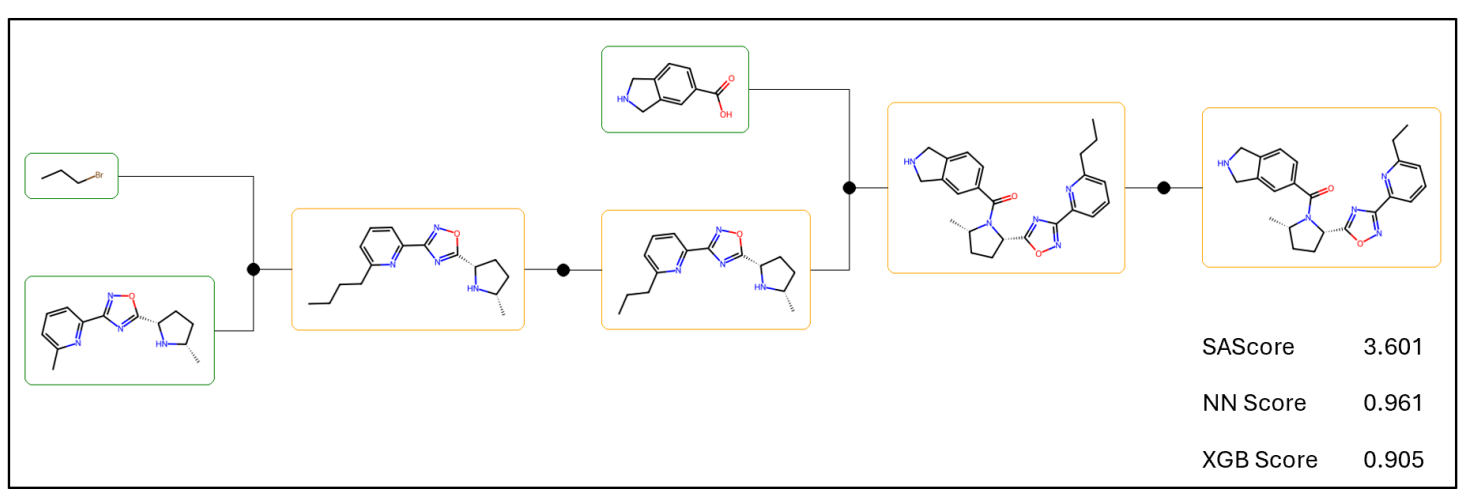

CUE8154

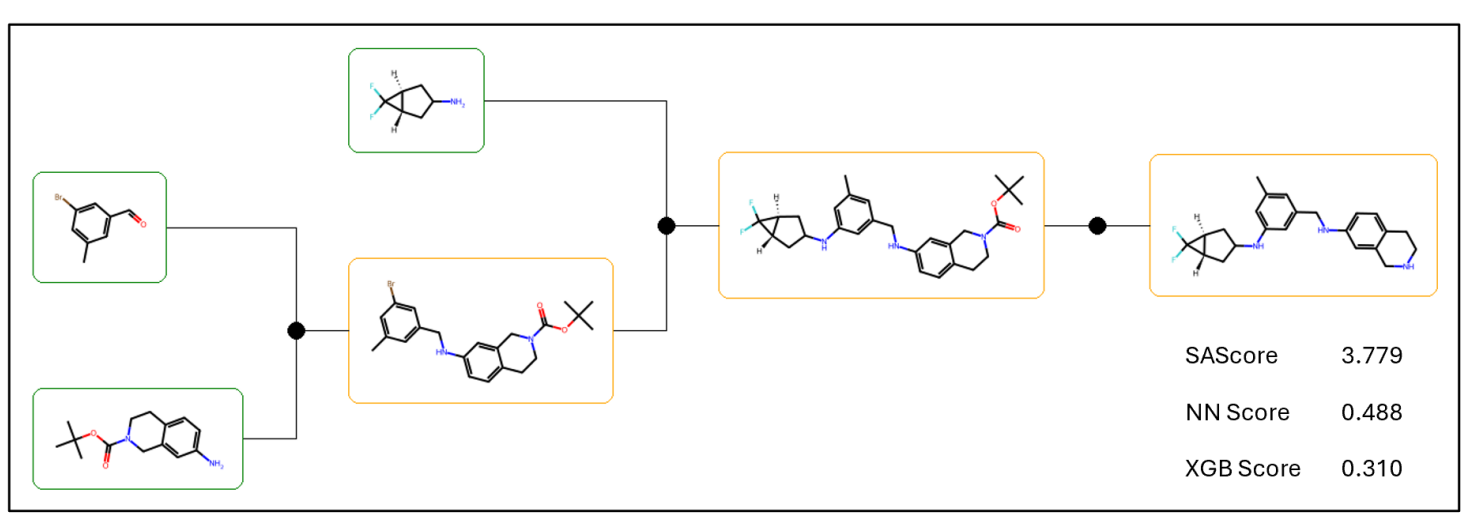

Supplementary Figure S11(Cont.): Retrosynthesis analysis of CUE-selected candidates: predicted retrosynthetic route generated by AiZynthFinder, with SAScore and RAScore(NN Score, XGB Score).

CUE8276

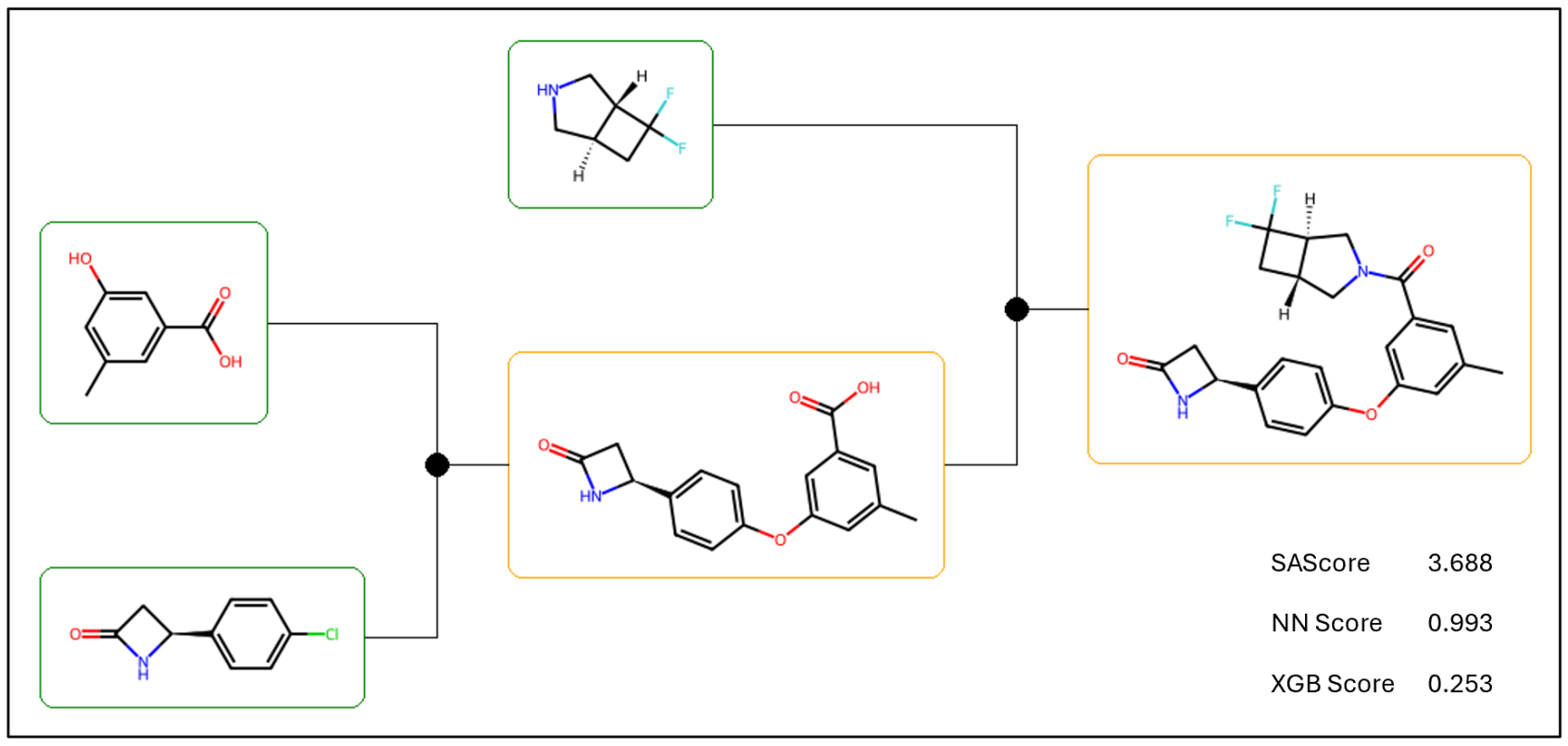

CUE8312

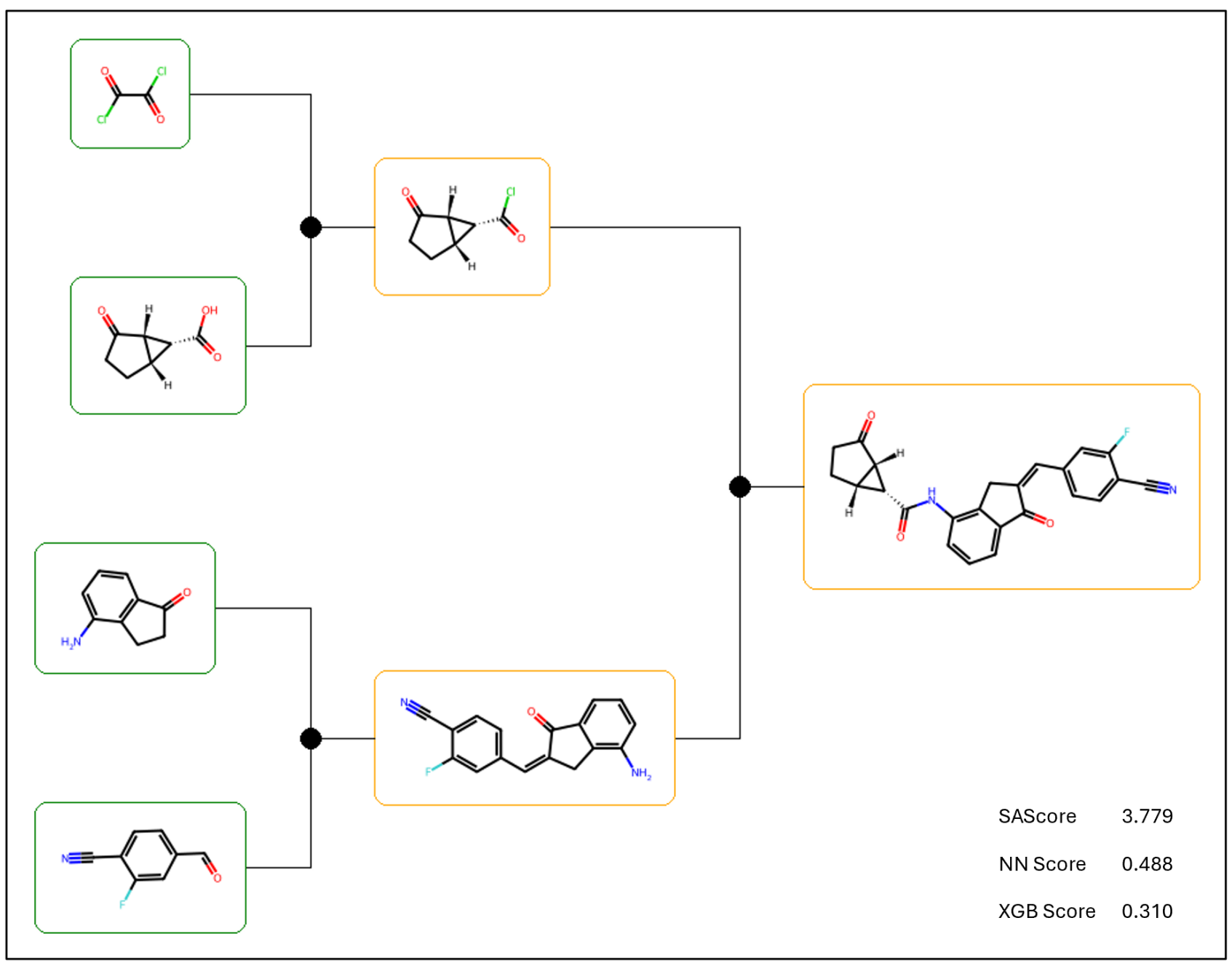

Supplementary Figure S11(Cont.): Retrosynthesis analysis of CUE-selected candidates: predicted retrosynthetic route generated by AiZynthFinder, with SAScore and RAScore(NN Score, XGB Score).

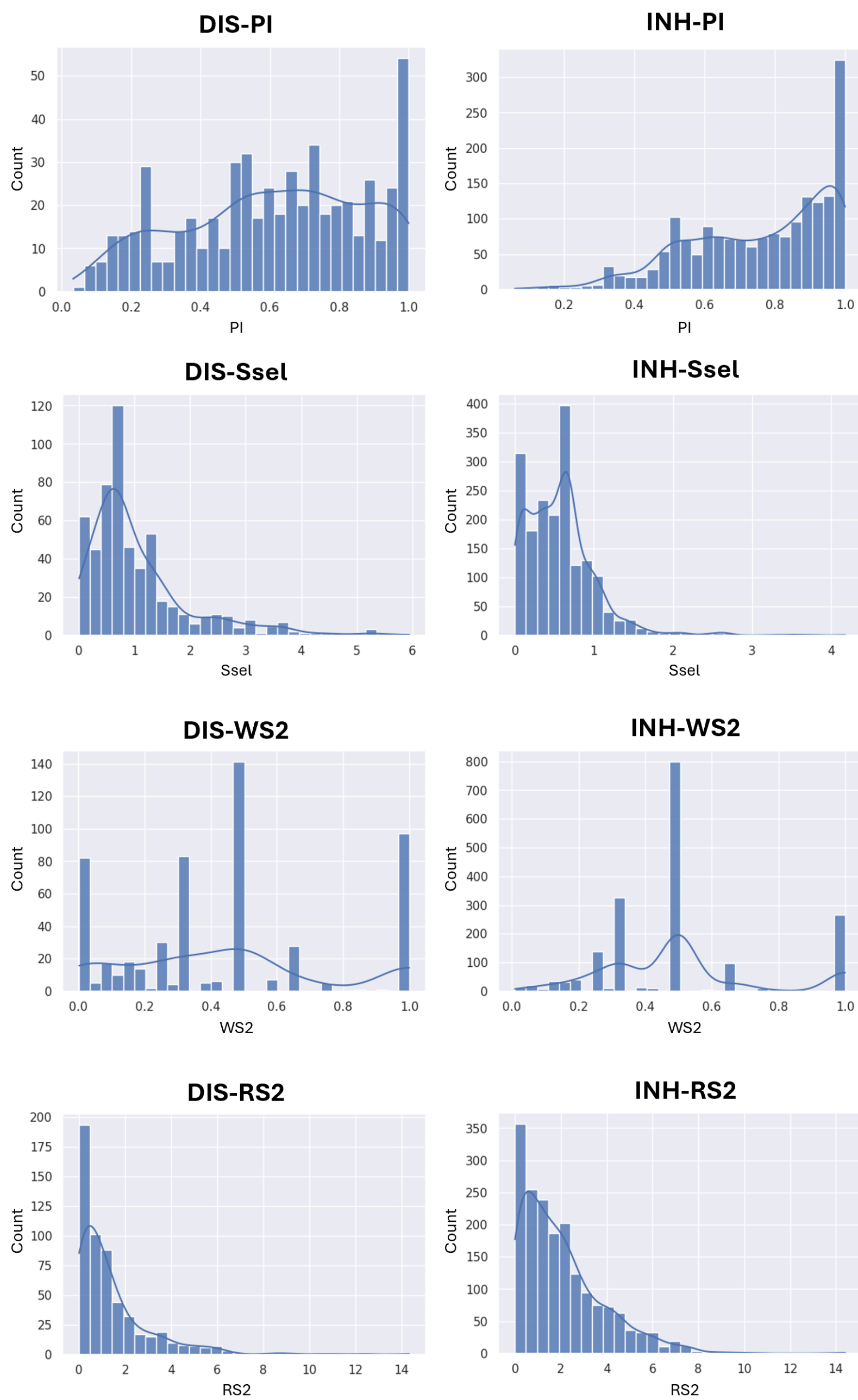

Supplementary Figure S12: Distributions of continuous selectivity scores across DIS and INH datasets

A3 Supplementary Tables

Supplementary Table S1: Number of samples in the selectivity dataset (compound-level) and the DTA dataset (compound–kinase pair-level) used in five-fold cross-validation. DIS: K_d-based dataset; INH: K_i-based dataset.

Group	Fold	Selectivity			DTA		
		Train	Validation	Test	Train	Validation	Test
DIS	1	399	45	112	26,120	2,708	5,589
	2	400	45	111	23,893	3,398	7,126
	3	400	45	111	22,207	2,609	9,601
	4	400	45	111	25,826	3,517	5,074
	5	400	45	111	23,172	4,218	7,027
INH	1	1,313	146	365	5,029	575	1,185
	2	1,313	146	365	4,723	634	1,432
	3	1,313	146	365	4,770	605	1,414
	4	1,313	146	365	5,034	555	1,200
	5	1,314	146	364	4,739	492	1,558

Supplementary Table S2: Summary of baseline models used for selectivity prediction performance comparison, including model category, input representation, and official implementation repository.

Category	Model	Input Representation	Official Repositories
DTA	DeepDTA	SMILES<br>Protein Sequence (FASTA)	`https://github.com/hkmztrk/DeepDTA`
	GraphDTA	Molecular Graph<br>Protein Sequence (FASTA)	`https://github.com/thinng/GraphDTA`
	TEFDTA	Molecular Graph<br>Protein Sequence (FASTA)	`https://github.com/RefDawn-XD/TEFDTA`
	HMM-DTA	SMILES<br>Protein Sequence (FASTA)	`https://github.com/amirhallaji/StructureFree-DTA`
QSAR	ADMET-PrInt	Molecular Graph	`https://github.com/JamEwe/ADMET-PrInt`
	ADMET-AI	Molecular Graph	`https://github.com/swansonk14/admet_ai`
	MMFDL	ECFP4 Fingerprints<br>SMILES<br>Molecular Graph	`https://github.com/AIMedDrug/MMFDL`
	MMFRL	ECFP4 Fingerprints<br>SMILES<br>Molecular Graph<br>Molecular Image<br>NMR spectrum<br>NMR peaks	`https://github.com/zhengyjo/MMFRL`

Supplementary Table S3: Predictive performance of CUE and the baseline models on the four DIS benchmarks (DIS-PI, DIS-Ssel, DIS-WS2, and DIS-RS2), evaluated by RMSE, R^2, and PCC. Values are reported as mean ± standard deviation across compound-level 5-fold cross-validation. Bold indicates the best performance and underlining the second best for each metric within each benchmark.

Dataset	Category	Model	RMSE(↓)	R^2(↑)	PCC(↑)
DIS-PI	DTA	DeepDTA	0.349 ± 0.018	-0.832 ± 0.223	$\underline{0.567 \pm 0.032}$
		GraphDTA	0.338 ± 0.037	-0.732 ± 0.411	$\mathbf{0.574 \pm 0.036}$
		TEFDTA	0.350 ± 0.021	-0.834 ± 0.174	0.566 ± 0.037
		HMM-DTA	0.276 ± 0.042	-0.153 ± 0.313	0.543 ± 0.090
	QSAR	ADMET-PrInt	0.270 ± 0.012	-0.096 ± 0.149	0.219 ± 0.047
		ADMET-AI	$\underline{0.258 \pm 0.010}$	-0.005 ± 0.046	0.181 ± 0.125
		MMFDL	0.264 ± 0.008	-0.043 ± 0.059	0.222 ± 0.052
		MMFRL	$\underline{0.258 \pm 0.010}$	$\underline{0.005 \pm 0.058}$	0.372 ± 0.058
		CUE	$\mathbf{0.242 \pm 0.010}$	$\mathbf{0.125 \pm 0.055}$	0.366 ± 0.092
DIS-Ssel	DTA	DeepDTA	1.411 ± 0.216	-1.389 ± 0.751	$\mathbf{0.800 \pm 0.044}$
		GraphDTA	1.354 ± 0.211	-1.194 ± 0.658	$\mathbf{0.800 \pm 0.044}$
		TEFDTA	1.392 ± 0.235	-1.340 ± 0.815	$\mathbf{0.800 \pm 0.044}$
		HMM-DTA	1.012 ± 0.262	-0.251 ± 0.571	$\underline{0.791 \pm 0.053}$
	QSAR	ADMET-PrInt	0.971 ± 0.109	-0.100 ± 0.175	0.208 ± 0.091
		ADMET-AI	$\underline{0.921 \pm 0.076}$	0.007 ± 0.042	0.220 ± 0.112
		MMFDL	0.922 ± 0.060	$\underline{0.010 \pm 0.075}$	0.197 ± 0.107
		MMFRL	0.931 ± 0.075	-0.016 ± 0.141	0.345 ± 0.087
		CUE	$\mathbf{0.895 \pm 0.068}$	$\mathbf{0.065 \pm 0.107}$	0.314 ± 0.104
DIS-WS2	DTA	DeepDTA	0.486 ± 0.033	-1.374 ± 0.630	$\underline{0.599 \pm 0.050}$
		GraphDTA	0.439 ± 0.068	-0.990 ± 0.830	$\mathbf{0.622 \pm 0.049}$
		TEFDTA	0.501 ± 0.090	-1.485 ± 0.727	0.585 ± 0.026
		HMM-DTA	0.341 ± 0.019	-0.148 ± 0.147	0.554 ± 0.047
	QSAR	ADMET-PrInt	$\underline{0.308 \pm 0.020}$	$\underline{0.069 \pm 0.106}$	0.413 ± 0.112
		ADMET-AI	0.316 ± 0.020	0.016 ± 0.044	0.269 ± 0.088
		MMFDL	0.311 ± 0.018	0.049 ± 0.064	0.312 ± 0.072
		MMFRL	0.311 ± 0.018	0.048 ± 0.116	0.407 ± 0.072
		CUE	$\mathbf{0.282 \pm 0.014}$	$\mathbf{0.221 \pm 0.061}$	0.490 ± 0.052
DIS-RS2	DTA	DeepDTA	2.085 ± 0.240	-0.512 ± 0.053	0.177 ± 0.123
		GraphDTA	2.054 ± 0.261	-0.470 ± 0.143	0.197 ± 0.193
		TEFDTA	2.088 ± 0.230	-0.520 ± 0.110	0.171 ± 0.136
		HMM-DTA	1.859 ± 0.275	-0.202 ± 0.165	0.217 ± 0.151
	QSAR	ADMET-PrInt	1.779 ± 0.255	-0.098 ± 0.100	0.136 ± 0.058
		ADMET-AI	1.713 ± 0.203	-0.014 ± 0.047	0.147 ± 0.045
		MMFDL	$\underline{1.694 \pm 0.186}$	$\underline{0.001 \pm 0.015}$	0.152 ± 0.019
		MMFRL	1.758 ± 0.138	-0.082 ± 0.072	$\underline{0.280 \pm 0.072}$
		CUE	$\mathbf{1.691 \pm 0.188}$	$\mathbf{0.002 \pm 0.115}$	$\mathbf{0.293 \pm 0.058}$

Supplementary Table S4: Predictive performance of CUE and the baseline models on the four INH benchmarks (INH-PI, INH-Ssel, INH-WS2, and INH-RS2), evaluated by RMSE, R^2, and PCC. Values are reported as mean $\pm$ standard deviation across compound-level 5-fold cross-validation. Bold indicates the best performance and underlining the second best for each metric within each benchmark.

Dataset	Category	Model	RMSE(↓)	R^2(↑)	PCC(↑)
INH-PI	DTA	DeepDTA	0.257 ± 0.025	-0.636 ± 0.304	0.411 ± 0.088
		GraphDTA	0.266 ± 0.030	-0.754 ± 0.351	0.371 ± 0.036
		TEFDTA	0.285 ± 0.017	-1.010 ± 0.181	0.324 ± 0.034
		HMM-DTA	0.192 ± 0.012	0.081 ± 0.150	0.575 ± 0.062
	QSAR	ADMET-PrInt	0.187 ± 0.004	0.134 ± 0.052	0.471 ± 0.081
		ADMET-AI	0.169 ± 0.004	0.288 ± 0.082	0.556 ± 0.059
		MMFDL	0.188 ± 0.005	0.129 ± 0.067	0.386 ± 0.067
		MMFRL	0.170 ± 0.004	0.287 ± 0.062	0.588 ± 0.036
		CUE	**0.163 ± 0.004**	**0.344 ± 0.053**	**0.597 ± 0.040**
INH-Ssel	DTA	DeepDTA	0.506 ± 0.053	-0.357 ± 0.330	0.609 ± 0.065
		GraphDTA	0.505 ± 0.066	-0.335 ± 0.279	0.600 ± 0.046
		TEFDTA	0.543 ± 0.046	-0.542 ± 0.197	0.580 ± 0.050
		HMM-DTA	0.378 ± 0.039	0.252 ± 0.121	0.723 ± 0.040
	QSAR	ADMET-PrInt	0.369 ± 0.047	0.290 ± 0.110	0.589 ± 0.061
		ADMET-AI	0.344 ± 0.034	0.388 ± 0.060	0.643 ± 0.041
		MMFDL	0.385 ± 0.045	0.230 ± 0.067	0.485 ± 0.064
		MMFRL	0.325 ± 0.039	0.451 ± 0.074	0.695 ± 0.042
		CUE	**0.322 ± 0.034**	**0.460 ± 0.061**	**0.696 ± 0.039**
INH-WS2	DTA	DeepDTA	0.312 ± 0.032	-0.657 ± 0.401	0.361 ± 0.105
		GraphDTA	0.340 ± 0.049	-0.990 ± 0.698	0.310 ± 0.062
		TEFDTA	0.351 ± 0.023	-1.085 ± 0.386	0.331 ± 0.036
		HMM-DTA	0.240 ± 0.010	0.034 ± 0.117	**0.521 ± 0.053**
	QSAR	ADMET-PrInt	0.223 ± 0.008	0.165 ± 0.058	0.430 ± 0.033
		ADMET-AI	0.222 ± 0.017	0.175 ± 0.090	0.453 ± 0.081
		MMFDL	0.235 ± 0.012	0.076 ± 0.040	0.301 ± 0.056
		MMFRL	0.231 ± 0.011	0.110 ± 0.046	0.469 ± 0.043
		CUE	**0.212 ± 0.010**	**0.249 ± 0.030**	0.509 ± 0.028
INH-RS2	DTA	DeepDTA	2.098 ± 0.206	-0.353 ± 0.209	0.288 ± 0.139
		GraphDTA	2.143 ± 0.169	-0.415 ± 0.221	0.255 ± 0.069
		TEFDTA	2.290 ± 0.082	-0.612 ± 0.142	0.118 ± 0.090
		HMM-DTA	1.610 ± 0.074	0.200 ± 0.117	0.553 ± 0.075
	QSAR	ADMET-PrInt	1.732 ± 0.100	0.070 ± 0.171	0.422 ± 0.057
		ADMET-AI	1.529 ± 0.061	0.282 ± 0.038	0.537 ± 0.033
		MMFDL	1.632 ± 0.062	0.182 ± 0.061	0.428 ± 0.070
		MMFRL	1.474 ± 0.030	0.331 ± 0.059	**0.618 ± 0.032**
		CUE	**1.452 ± 0.062**	**0.350 ± 0.071**	0.608 ± 0.041

Supplementary Table S5: Comparison of fingerprint-only (FP), molecular image-only (IMG), equal-weighting (Uniform), and CUE models on the DIS dataset via 5-fold cross-validation.

Dataset	Model	RMSE(↓)	R^2(↑)	PCC(↑)
DIS-PI	FP	<u>0.245 ± 0.012</u>	0.098 ± 0.059	0.357 ± 0.085
	IMG	0.257 ± 0.007	0.008 ± 0.043	0.194 ± 0.084
	Uniform	**0.242 ± 0.010**	<u>0.121 ± 0.059</u>	<u>0.361 ± 0.097</u>
	CUE	**0.242 ± 0.010**	**0.125 ± 0.055**	**0.366 ± 0.092**
DIS-Ssel	FP	**0.887 ± 0.042**	**0.081 ± 0.073**	**0.326 ± 0.072**
	IMG	0.957 ± 0.105	-0.072 ± 0.188	0.189 ± 0.095
	Uniform	0.901 ± 0.074	0.051 ± 0.118	0.303 ± 0.110
	CUE	<u>0.895 ± 0.068</u>	<u>0.065 ± 0.107</u>	<u>0.314 ± 0.104</u>
DIS-WS2	FP	0.285 ± 0.018	0.203 ± 0.052	0.470 ± 0.044
	IMG	0.300 ± 0.009	0.114 ± 0.098	0.380 ± 0.096
	Uniform	**0.282 ± 0.012**	<u>0.215 ± 0.069</u>	<u>0.488 ± 0.057</u>
	CUE	<u>0.282 ± 0.014</u>	**0.221 ± 0.061**	**0.490 ± 0.052**
DIS-RS2	FP	1.737 ± 0.195	-0.053 ± 0.122	**0.299 ± 0.039**
	IMG	1.806 ± 0.280	-0.131 ± 0.167	0.113 ± 0.046
	Uniform	<u>1.695 ± 0.209</u>	<u>-0.001 ± 0.117</u>	0.287 ± 0.063
	CUE	**1.691 ± 0.188**	**0.002 ± 0.115**	<u>0.293 ± 0.058</u>

*Bold: best performance. Underline: second-best performance.

Supplementary Table S6: Comparison of fingerprint-only (FP), molecular image-only (IMG), equal-weighting (Uniform), and CUE models on the INH dataset via 5-fold cross-validation.

Dataset	Model	RMSE(↓)	R^2(↑)	PCC(↑)
INH-PI	FP	0.166 ± 0.007	0.317 ± 0.058	0.585 ± 0.033
	IMG	0.177 ± 0.005	0.220 ± 0.072	0.486 ± 0.066
	Uniform	<u>0.164 ± 0.003</u>	<u>0.335 ± 0.056</u>	<u>0.590 ± 0.040</u>
	CUE	**0.163 ± 0.004**	**0.344 ± 0.053**	**0.597 ± 0.040**
INH-Ssel	FP	<u>0.327 ± 0.043</u>	0.446 ± 0.069	<u>0.691 ± 0.038</u>
	IMG	0.366 ± 0.038	0.302 ± 0.083	0.577 ± 0.048
	Uniform	**0.322 ± 0.034**	<u>0.459 ± 0.066</u>	0.691 ± 0.040
	CUE	**0.322 ± 0.034**	**0.460 ± 0.061**	**0.696 ± 0.039**
INH-WS2	FP	<u>0.214 ± 0.009</u>	0.234 ± 0.029	0.497 ± 0.025
	IMG	0.230 ± 0.016	0.114 ± 0.072	0.327 ± 0.116
	Uniform	**0.212 ± 0.010**	<u>0.246 ± 0.028</u>	<u>0.507 ± 0.028</u>
	CUE	**0.212 ± 0.010**	**0.249 ± 0.030**	**0.509 ± 0.028**
INH-RS2	FP	**1.450 ± 0.045**	**0.353 ± 0.053**	0.605 ± 0.034
	IMG	1.615 ± 0.060	0.197 ± 0.076	0.450 ± 0.080
	Uniform	1.455 ± 0.064	0.348 ± 0.071	<u>0.607 ± 0.041</u>
	CUE	<u>1.452 ± 0.062</u>	<u>0.350 ± 0.071</u>	**0.608 ± 0.041**

*Bold: best performance. Underline: second-best performance.

Supplementary Table S7: Comparison of feature fusion strategies (Element-wise summation (Sum), Early concatenation (Concat(E)), Intermediate concatenation (Concat(I)), and CUE) on the DIS dataset via 5-fold cross-validation.

Dataset	Model	RMSE(↓)	R^2(↑)	PCC(↑)
DIS-PI	Sum	$\underline{0.242 \pm 0.010}$	0.124 ± 0.057	0.363 ± 0.096
	Concat(E)	$\mathbf{0.237 \pm 0.012}$	$\mathbf{0.159 \pm 0.064}$	$\mathbf{0.419 \pm 0.077}$
	Concat(I)	0.242 ± 0.011	0.119 ± 0.060	0.361 ± 0.093
	CUE	$\underline{0.242 \pm 0.010}$	$\underline{0.125 \pm 0.055}$	$\underline{0.366 \pm 0.092}$
DIS-Ssel	Sum	0.904 ± 0.074	0.046 ± 0.119	0.300 ± 0.109
	Concat(E)	$\mathbf{0.893 \pm 0.060}$	$\mathbf{0.071 \pm 0.079}$	$\mathbf{0.334 \pm 0.067}$
	Concat(I)	0.899 ± 0.072	0.056 ± 0.107	0.302 ± 0.105
	CUE	$\underline{0.895 \pm 0.068}$	$\underline{0.065 \pm 0.107}$	$\underline{0.314 \pm 0.104}$
DIS-WS2	Sum	$\underline{0.283 \pm 0.013}$	$\underline{0.211 \pm 0.076}$	$\underline{0.487 \pm 0.059}$
	Concat(E)	0.293 ± 0.024	0.154 ± 0.127	0.459 ± 0.059
	Concat(I)	0.284 ± 0.014	0.206 ± 0.067	0.482 ± 0.057
	CUE	$\mathbf{0.282 \pm 0.014}$	$\mathbf{0.221 \pm 0.061}$	$\mathbf{0.490 \pm 0.052}$
DIS-RS2	Sum	1.717 ± 0.221	-0.030 ± 0.155	0.285 ± 0.066
	Concat(E)	1.727 ± 0.133	-0.050 ± 0.138	0.276 ± 0.040
	Concat(I)	$\mathbf{1.689 \pm 0.191}$	$\mathbf{0.004 \pm 0.116}$	$\underline{0.291 \pm 0.059}$
	CUE	$\underline{1.691 \pm 0.188}$	$\underline{0.002 \pm 0.115}$	$\mathbf{0.293 \pm 0.058}$

*Bold: best performance. Underline: second-best performance.

Supplementary Table S8: Comparison of feature fusion strategies (Element-wise summation (Sum), Early concatenation (Concat(E)), Intermediate concatenation (Concat(I)), and CUE) on the INH dataset via 5-fold cross-validation.

Dataset	Model	RMSE(↓)	R^2(↑)	PCC(↑)
INH-PI	Sum	**0.163 ± 0.002**	<u>0.341 ± 0.058</u>	<u>0.593 ± 0.044</u>
	Concat(E)	**0.163 ± 0.002**	0.339 ± 0.062	0.592 ± 0.040
	Concat(I)	0.165 ± 0.004	0.329 ± 0.057	0.587 ± 0.041
	CUE	<u>0.163 ± 0.004</u>	**0.344 ± 0.053**	**0.597 ± 0.040**
INH-Ssel	Sum	<u>0.321 ± 0.035</u>	<u>0.463 ± 0.069</u>	<u>0.695 ± 0.042</u>
	Concat(E)	0.331 ± 0.041	0.430 ± 0.075	0.677 ± 0.041
	Concat(I)	**0.321 ± 0.034**	**0.464 ± 0.062**	0.692 ± 0.038
	CUE	0.322 ± 0.034	0.460 ± 0.061	**0.696 ± 0.039**
INH-WS2	Sum	**0.212 ± 0.010**	0.244 ± 0.031	0.505 ± 0.028
	Concat(E)	<u>0.216 ± 0.012</u>	0.216 ± 0.059	0.488 ± 0.043
	Concat(I)	**0.212 ± 0.010**	**0.249 ± 0.029**	<u>0.507 ± 0.028</u>
	CUE	**0.212 ± 0.010**	<u>0.249 ± 0.030</u>	**0.509 ± 0.028**
INH-RS2	Sum	<u>1.452 ± 0.061</u>	<u>0.351 ± 0.069</u>	<u>0.608 ± 0.040</u>
	Concat(E)	1.481 ± 0.058	0.325 ± 0.067	0.588 ± 0.043
	Concat(I)	**1.446 ± 0.050**	**0.356 ± 0.059**	**0.608 ± 0.039**
	CUE	1.452 ± 0.062	0.350 ± 0.071	0.608 ± 0.041

*Bold: best performance. Underline: second-best performance.

Supplementary Table S9(Cont.): Performance of CUE across different LTAU hyperparameter settings, the number of histogram bins (B) and the number of nearest neighbors (K), on the DIS dataset via 5-fold cross-validation.

Target	BIN	K	RMSE($\downarrow$)	R^2($\uparrow$)	PCC($\uparrow$)
DIS-PI	5	3	0.246 ± 0.012	0.096 ± 0.061	0.345 ± 0.097
		5	0.245 ± 0.012	0.098 ± 0.060	0.346 ± 0.094
		7	0.244 ± 0.011	0.104 ± 0.062	0.346 ± 0.095
	50	3	0.244 ± 0.011	0.111 ± 0.059	0.354 ± 0.087
		5	0.243 ± 0.011	0.112 ± 0.059	0.355 ± 0.087
		7	0.243 ± 0.011	0.112 ± 0.059	0.356 ± 0.090
	500	3	0.243 ± 0.011	0.113 ± 0.059	0.356 ± 0.089
		5	0.243 ± 0.011	0.113 ± 0.059	0.356 ± 0.090
		7	0.243 ± 0.011	0.113 ± 0.060	0.356 ± 0.092
	5000	3	$\mathbf{0.241 \pm 0.010}$	$\mathbf{0.126 \pm 0.055}$	$\mathbf{0.366 \pm 0.091}$
		5	$\underline{0.242 \pm 0.010}$	$\underline{0.125 \pm 0.055}$	$\underline{0.366 \pm 0.092}$
		7	$\underline{0.242 \pm 0.010}$	0.124 ± 0.055	0.365 ± 0.092
	50000	3	0.243 ± 0.011	0.112 ± 0.064	0.353 ± 0.094
		5	0.243 ± 0.011	0.112 ± 0.064	0.354 ± 0.095
		7	0.243 ± 0.012	0.112 ± 0.064	0.354 ± 0.095
DIS-Ssel	5	3	0.902 ± 0.073	0.050 ± 0.111	0.300 ± 0.104
		5	0.903 ± 0.072	0.048 ± 0.117	0.305 ± 0.106
		7	0.904 ± 0.071	0.044 ± 0.116	0.302 ± 0.104
	50	3	0.898 ± 0.069	0.058 ± 0.103	0.308 ± 0.101
		5	0.898 ± 0.069	0.059 ± 0.104	0.311 ± 0.103
		7	0.899 ± 0.068	0.057 ± 0.103	0.308 ± 0.101
	500	3	0.897 ± 0.067	0.061 ± 0.100	0.310 ± 0.101
		5	0.900 ± 0.063	0.052 ± 0.109	0.312 ± 0.103
		7	0.897 ± 0.066	0.060 ± 0.099	0.310 ± 0.101
	5000	3	$\underline{0.895 \pm 0.068}$	0.064 ± 0.107	0.313 ± 0.104
		5	$\underline{0.895 \pm 0.068}$	$\mathbf{0.065 \pm 0.107}$	$\mathbf{0.314 \pm 0.104}$
		7	$\mathbf{0.895 \pm 0.067}$	$\underline{0.064 \pm 0.106}$	$\underline{0.313 \pm 0.103}$
	50000	3	0.904 ± 0.074	0.045 ± 0.117	0.301 ± 0.108
		5	0.903 ± 0.073	0.046 ± 0.116	0.303 ± 0.107
		7	0.904 ± 0.072	0.046 ± 0.114	0.302 ± 0.106

Supplementary Table S9: Performance of CUE across different LTAU hyperparameter settings, the number of histogram bins (B) and the number of nearest neighbors (K), on the DIS dataset via 5-fold cross-validation.

Target	BIN	K	RMSE(↓)	R^2(↑)	PCC(↑)
DIS-WS2	5	3	<u>0.283 ± 0.012</u>	0.210 ± 0.080	0.491 ± 0.060
		5	<u>0.283 ± 0.012</u>	0.210 ± 0.080	0.491 ± 0.060
		7	<u>0.283 ± 0.012</u>	0.210 ± 0.080	0.490 ± 0.060
	50	3	**0.282 ± 0.014**	0.215 ± 0.075	0.491 ± 0.058
		5	0.283 ± 0.013	0.210 ± 0.079	<u>0.492 ± 0.058</u>
		7	0.283 ± 0.013	0.209 ± 0.079	<u>0.492 ± 0.058</u>
	500	3	<u>0.283 ± 0.012</u>	0.211 ± 0.077	<u>0.492 ± 0.058</u>
		5	0.283 ± 0.012	0.212 ± 0.076	**0.493 ± 0.058**
		7	0.283 ± 0.013	0.211 ± 0.077	<u>0.492 ± 0.058</u>
	5000	3	**0.282 ± 0.014**	**0.221 ± 0.061**	0.490 ± 0.052
		5	**0.282 ± 0.014**	**0.221 ± 0.061**	0.490 ± 0.052
		7	**0.282 ± 0.014**	<u>0.220 ± 0.061</u>	0.490 ± 0.052
	50000	3	0.284 ± 0.012	0.208 ± 0.082	0.490 ± 0.060
		5	0.284 ± 0.013	0.207 ± 0.082	0.490 ± 0.060
		7	0.284 ± 0.013	0.205 ± 0.083	0.489 ± 0.061
DIS-RS2	5	3	1.718 ± 0.231	−0.030 ± 0.161	0.282 ± 0.067
		5	1.715 ± 0.232	−0.027 ± 0.160	0.286 ± 0.069
		7	1.714 ± 0.233	−0.025 ± 0.159	0.282 ± 0.067
	50	3	1.714 ± 0.223	−0.026 ± 0.155	0.289 ± 0.062
		5	1.714 ± 0.221	−0.025 ± 0.153	0.289 ± 0.061
		7	1.714 ± 0.220	−0.026 ± 0.150	0.288 ± 0.059
	500	3	1.708 ± 0.210	−0.020 ± 0.151	0.290 ± 0.057
		5	<u>1.707 ± 0.209</u>	−0.020 ± 0.150	0.290 ± 0.057
		7	1.708 ± 0.208	−0.020 ± 0.149	0.289 ± 0.056
	5000	3	**1.691 ± 0.188**	**0.002 ± 0.115**	**0.293 ± 0.057**
		5	**1.691 ± 0.188**	**0.002 ± 0.115**	<u>0.293 ± 0.058</u>
		7	**1.691 ± 0.188**	<u>0.001 ± 0.114</u>	**0.293 ± 0.057**
	50000	3	1.716 ± 0.220	−0.028 ± 0.150	0.287 ± 0.061
		5	1.716 ± 0.220	−0.029 ± 0.151	0.287 ± 0.061
		7	1.717 ± 0.220	−0.029 ± 0.151	0.287 ± 0.060

*Bold: best performance. Underline: second-best performance.

Supplementary Table S10(Cont.): Performance of CUE across different LTAU hyperparameter settings, the number of histogram bins (B) and the number of nearest neighbors (K), on the INH dataset via 5-fold cross-validation.

Target	BIN	K	RMSE(↓)	R^2(↑)	PCC(↑)
INH-PI	5	3	0.166 ± 0.004	0.320 ± 0.054	0.581 ± 0.043
		5	0.166 ± 0.004	0.321 ± 0.054	0.582 ± 0.043
		7	0.166 ± 0.004	0.321 ± 0.053	0.582 ± 0.042
	50	3	0.165 ± 0.005	0.326 ± 0.054	0.585 ± 0.040
		5	0.165 ± 0.005	0.325 ± 0.054	0.585 ± 0.040
		7	0.165 ± 0.005	0.325 ± 0.054	0.585 ± 0.040
	500	3	0.166 ± 0.005	0.320 ± 0.055	0.584 ± 0.041
		5	0.166 ± 0.005	0.321 ± 0.055	0.584 ± 0.041
		7	0.166 ± 0.005	0.321 ± 0.055	0.584 ± 0.041
	5000	3	**0.163 ± 0.004**	**0.344 ± 0.053**	**0.597 ± 0.040**
		5	**0.163 ± 0.004**	**0.344 ± 0.053**	**0.597 ± 0.040**
		7	**0.163 ± 0.004**	**0.344 ± 0.053**	**0.597 ± 0.040**
	50000	3	0.166 ± 0.005	0.319 ± 0.058	0.581 ± 0.044
		5	0.166 ± 0.005	0.318 ± 0.059	0.581 ± 0.043
		7	<u>0.165 ± 0.004</u>	<u>0.329 ± 0.053</u>	<u>0.587 ± 0.043</u>
INH-Ssel	5	3	0.325 ± 0.037	0.451 ± 0.058	0.693 ± 0.037
		5	0.325 ± 0.037	0.453 ± 0.059	0.693 ± 0.037
		7	0.325 ± 0.036	0.451 ± 0.058	0.693 ± 0.037
	50	3	0.324 ± 0.034	0.456 ± 0.056	0.695 ± 0.039
		5	0.323 ± 0.034	0.456 ± 0.055	0.695 ± 0.038
		7	0.324 ± 0.034	0.456 ± 0.056	0.695 ± 0.039
	500	3	0.323 ± 0.034	0.457 ± 0.058	0.695 ± 0.039
		5	0.323 ± 0.034	0.457 ± 0.058	0.695 ± 0.039
		7	0.323 ± 0.034	0.457 ± 0.058	0.695 ± 0.039
	5000	3	**0.322 ± 0.034**	0.459 ± 0.061	0.695 ± 0.040
		5	**0.322 ± 0.034**	**0.460 ± 0.061**	**0.696 ± 0.039**
		7	**0.322 ± 0.034**	0.459 ± 0.062	<u>0.696 ± 0.040</u>
	50000	3	<u>0.322 ± 0.035</u>	0.458 ± 0.068	0.693 ± 0.040
		5	<u>0.322 ± 0.035</u>	0.459 ± 0.065	0.693 ± 0.039
		7	<u>0.322 ± 0.035</u>	<u>0.460 ± 0.067</u>	0.693 ± 0.039

Supplementary Table S10: Performance of CUE across different LTAU hyperparameter settings, the number of histogram bins (B) and the number of nearest neighbors (K), on the INH dataset via 5-fold cross-validation.

Target	BIN	K	RMSE(↓)	R^2(↑)	PCC(↑)
INH-WS2	5	3	<u>0.213 ± 0.011</u>	0.243 ± 0.032	0.499 ± 0.030
		5	**0.212 ± 0.010**	0.244 ± 0.033	0.502 ± 0.028
		7	**0.212 ± 0.010**	0.245 ± 0.033	0.504 ± 0.027
	50	3	**0.212 ± 0.010**	0.247 ± 0.030	0.503 ± 0.028
		5	**0.212 ± 0.010**	0.248 ± 0.029	0.504 ± 0.028
		7	**0.212 ± 0.010**	0.248 ± 0.029	0.505 ± 0.027
	500	3	**0.212 ± 0.010**	0.245 ± 0.033	0.504 ± 0.027
		5	**0.212 ± 0.010**	0.246 ± 0.033	0.505 ± 0.027
		7	**0.212 ± 0.010**	0.246 ± 0.033	0.505 ± 0.027
	5000	3	**0.212 ± 0.010**	0.248 ± 0.030	**0.509 ± 0.028**
		5	**0.212 ± 0.010**	<u>0.249 ± 0.030</u>	**0.509 ± 0.028**
		7	**0.212 ± 0.010**	0.249 ± 0.031	**0.509 ± 0.028**
	50000	3	**0.212 ± 0.010**	**0.249 ± 0.029**	<u>0.505 ± 0.026</u>
		5	**0.212 ± 0.010**	**0.249 ± 0.029**	<u>0.506 ± 0.026</u>
		7	**0.212 ± 0.010**	**0.249 ± 0.029**	<u>0.506 ± 0.026</u>
INH-RS2	5	3	1.444 ± 0.049	0.358 ± 0.060	0.606 ± 0.042
		5	1.446 ± 0.051	0.356 ± 0.062	0.606 ± 0.042
		7	1.447 ± 0.051	0.355 ± 0.063	0.605 ± 0.043
	50	3	<u>1.444 ± 0.048</u>	0.358 ± 0.060	**0.610 ± 0.041**
		5	1.446 ± 0.050	0.356 ± 0.062	0.609 ± 0.041
		7	1.448 ± 0.050	0.355 ± 0.063	0.608 ± 0.042
	500	3	**1.443 ± 0.046**	**0.359 ± 0.057**	<u>0.609 ± 0.040</u>
		5	1.445 ± 0.048	<u>0.358 ± 0.059</u>	0.609 ± 0.040
		7	1.446 ± 0.048	0.357 ± 0.060	0.608 ± 0.041
	5000	3	1.451 ± 0.061	0.351 ± 0.070	0.609 ± 0.041
		5	1.452 ± 0.062	0.350 ± 0.071	0.608 ± 0.041
		7	1.453 ± 0.062	0.349 ± 0.071	0.608 ± 0.041
	50000	3	1.457 ± 0.065	0.346 ± 0.073	0.606 ± 0.041
		5	1.458 ± 0.067	0.344 ± 0.075	0.606 ± 0.041
		7	1.451 ± 0.054	0.352 ± 0.066	0.605 ± 0.041

*Bold: best performance. Underline: second-best performance.

Supplementary Table S11(Cont.): Performance of CUE across different values of the uncertainty-weighting temperature parameter τ on the DIS and INH benchmark datasets via 5-fold cross-validation.

Dataset	τ	RMSE(↓)	R^2(↑)	PCC(↑)
DIS-PI	0.25	**0.243 ± 0.011**	**0.117 ± 0.058**	**0.361 ± 0.087**
	0.5	**0.243 ± 0.011**	0.114 ± 0.060	0.358 ± 0.088
	1	**0.243 ± 0.011**	0.112 ± 0.062	0.355 ± 0.092
	2	**0.243 ± 0.011**	0.111 ± 0.063	0.353 ± 0.095
	4	0.244 ± 0.011	0.110 ± 0.063	0.352 ± 0.096
DIS-Ssel	0.25	**0.890 ± 0.048**	**0.074 ± 0.080**	**0.323 ± 0.088**
	0.5	0.894 ± 0.061	0.067 ± 0.094	0.318 ± 0.098
	1	0.899 ± 0.070	0.058 ± 0.101	0.309 ± 0.103
	2	0.902 ± 0.074	0.050 ± 0.119	0.306 ± 0.108
	4	0.906 ± 0.073	0.040 ± 0.125	0.302 ± 0.108
DIS-WS2	0.25	0.286 ± 0.014	0.195 ± 0.055	0.479 ± 0.044
	0.5	0.284 ± 0.013	0.207 ± 0.076	0.489 ± 0.057
	1	**0.283 ± 0.012**	**0.210 ± 0.079**	**0.490 ± 0.059**
	2	**0.283 ± 0.012**	0.209 ± 0.080	0.490 ± 0.060
	4	0.284 ± 0.013	0.206 ± 0.081	0.488 ± 0.060
DIS-RS2	0.25	**1.686 ± 0.179**	**0.006 ± 0.110**	**0.297 ± 0.046**
	0.5	1.701 ± 0.212	−0.011 ± 0.149	0.293 ± 0.054
	1	1.706 ± 0.210	−0.018 ± 0.152	0.289 ± 0.058
	2	1.714 ± 0.221	−0.026 ± 0.152	0.287 ± 0.062
	4	1.713 ± 0.227	−0.024 ± 0.152	0.284 ± 0.063
INH-PI	0.25	**0.165 ± 0.005**	**0.324 ± 0.049**	**0.585 ± 0.037**
	0.5	**0.165 ± 0.005**	0.322 ± 0.058	0.584 ± 0.040
	1	0.166 ± 0.005	0.321 ± 0.056	0.583 ± 0.042
	2	0.166 ± 0.005	0.320 ± 0.058	0.581 ± 0.043
	4	0.166 ± 0.005	0.320 ± 0.060	0.580 ± 0.045
INH-Ssel	0.25	**0.321 ± 0.035**	**0.464 ± 0.059**	**0.697 ± 0.040**
	0.5	0.323 ± 0.034	0.458 ± 0.059	0.697 ± 0.041
	1	**0.321 ± 0.035**	0.462 ± 0.066	0.694 ± 0.039
	2	0.322 ± 0.035	0.461 ± 0.067	0.693 ± 0.040
	4	0.322 ± 0.036	0.460 ± 0.068	0.693 ± 0.040

Supplementary Table S11: Performance of CUE across different values of the uncertainty-weighting temperature parameter τ on the DIS and INH benchmark datasets via 5-fold cross-validation.

Dataset	τ	RMSE($\downarrow$)	R^2($\uparrow$)	PCC($\uparrow$)
INH-WS2	0.25	$\mathbf{0.212 \pm 0.010}$	0.244 ± 0.033	0.502 ± 0.028
	0.5	$\mathbf{0.212 \pm 0.010}$	$\underline{0.248 \pm 0.031}$	0.505 ± 0.028
	1	$\mathbf{0.212 \pm 0.010}$	0.244 ± 0.032	$\underline{0.505 \pm 0.026}$
	2	$\mathbf{0.211 \pm 0.010}$	$\mathbf{0.251 \pm 0.026}$	$\mathbf{0.506 \pm 0.026}$
	4	$\underline{0.213 \pm 0.010}$	0.241 ± 0.039	0.503 ± 0.026
INH-RS2	0.25	$\mathbf{1.441 \pm 0.043}$	$\mathbf{0.361 \pm 0.054}$	$\underline{0.607 \pm 0.040}$
	0.5	1.448 ± 0.050	0.354 ± 0.064	$\mathbf{0.608 \pm 0.041}$
	1	$\underline{1.446 \pm 0.051}$	$\underline{0.356 \pm 0.063}$	0.607 ± 0.041
	2	1.456 ± 0.065	0.347 ± 0.071	0.606 ± 0.041
	4	1.457 ± 0.064	0.346 ± 0.071	0.606 ± 0.041

*Bold: best performance. Underline: second-best performance.

Supplementary Table S12: Statistical significance test (F-statistic and p-value) of performance variation across hyperparameter settings for each DIS and INH benchmark.

Dataset	Metric	F-statistic	p-value	Significant ($p<0.05$)
DIS-PI	RMSE	0.004	1.0000	False
	R^2	0.008	0.9998	False
	PCC	0.007	0.9999	False
DIS-Ssel	RMSE	0.035	0.9975	False
	R^2	0.065	0.9916	False
	PCC	0.029	0.9982	False
DIS-WS2	RMSE	0.040	0.9967	False
	R^2	0.026	0.9986	False
	PCC	0.028	0.9983	False
DIS-RS2	RMSE	0.012	0.9997	False
	R^2	0.032	0.9978	False
	PCC	0.035	0.9974	False
INH-PI	RMSE	0.004	1.0000	False
	R^2	0.003	1.0000	False
	PCC	0.009	0.9998	False
INH-Ssel	RMSE	0.002	1.0000	False
	R^2	0.005	0.9999	False
	PCC	0.010	0.9998	False
INH-WS2	RMSE	0.012	0.9997	False
	R^2	0.062	0.9922	False
	PCC	0.012	0.9997	False
INH-RS2	RMSE	0.059	0.9930	False
	R^2	0.038	0.9970	False
	PCC	0.002	1.0000	False

Supplementary Table S13: Summary of compounds identified through virtual screening for EGFR(T790M/C797S)-selective inhibitors

Molecule	SMILES
19g (Lategahn et al., 2021)	CCC(=O)Nc1cccc(-c2c(-c3ccc(N4CCN(C)CC4)cc3)[nH]c3ncnc(OC(C)C)c23)c1
CUE1602	Cc1c(NC(=O)[C@H]2CCCNC2)cc(F)cc1C(=O)N[C@@H]1Cc2ccccc2[C@@H]1C
CUE2191	N#Cc1ccc(F)c(NC(=O)[C@H]2C[C@@H]3CCC[C@H]3N2c2ccc3c(c2)COC3)c1F
CUE3201	O=C1Cc2cccc(Oc3ccc(-c4noc([C@]56CC[C@H]5C6)n4)cc3O)c2N1
CUE3417	CN(Cc1ccc(N2CCc3ccc(C=O)cc32)c(F)c1)C1CCC2CC1C2
CUE3886	COc1ccc(F)c(NC(=O)C2[C@H]3CN(c4cccc5c4CC[C@@H]5N)C[C@@H]23)c1
CUE4630	NC1(C(=O)N(c2ccc3c(c2)C(=O)CO3)[C@H]2C[C@H](c3ccccc3)C2)CNC1
CUE5412	Fc1ccc2c(c1)CN(c1cnn3c(N4CCN5CCOC[C@@H]5C4)ccnc13)C2
CUE5664	Cc1nccc2cccc(CN(c3cncc(F)c3C(=O)O)[C@@H]3CC[C@H]4C[C@@H]4C3)c12
CUE6182	O=C1Cc2ccc(N[C@@H]3CCC[C@H](C(=O)N[C@H]4c5ccccc5C[C@H]4O)C3)cc2N1
CUE6940	CCc1cccc(-c2noc([C@@H]3CC[C@H](C)N3C(=O)c3ccc4c(c3)CNC4)n2)n1
CUE8154	Cc1cc(CNc2ccc3c(c2)CNCC3)cc(NC2C[C@H]3[C@H](C2)C3(F)F)c1
CUE8276	Cc1cc(Oc2ccc([C@@H]3CC(=O)N3)cc2)cc(C(=O)N2C[C@@H]3CC(F)(F)[C@H]3C2)c1
CUE8312	N#Cc1ccc(C=C2Cc3c(NC(=O)[C@@H]4[C@@H]5CCC(=O)[C@@H]54)cccc3C2=O)cc1F

Supplementary Table S14: Performance comparison across single-modalities on DIS dataset.

Dataset	Model	RMSE(↓)	R^2(↑)	PCC(↑)
DIS-PI	FP	$\mathbf{0.245 \pm 0.012}$	$\mathbf{0.098 \pm 0.059}$	$\mathbf{0.357 \pm 0.085}$
	IMG	$\underline{0.257 \pm 0.007}$	$\underline{0.008 \pm 0.043}$	0.194 ± 0.084
	SMI	0.261 ± 0.006	-0.025 ± 0.071	0.118 ± 0.107
	Graph	0.260 ± 0.015	-0.014 ± 0.101	$\underline{0.243 \pm 0.115}$
	3D	0.274 ± 0.018	-0.130 ± 0.117	0.122 ± 0.155
DIS-Ssel	FP	$\mathbf{0.887 \pm 0.042}$	$\mathbf{0.081 \pm 0.073}$	$\mathbf{0.326 \pm 0.072}$
	IMG	0.957 ± 0.105	-0.072 ± 0.188	0.189 ± 0.095
	SMI	0.909 ± 0.068	0.038 ± 0.070	0.211 ± 0.138
	Graph	$\underline{0.904 \pm 0.056}$	$\underline{0.043 \pm 0.110}$	$\underline{0.324 \pm 0.096}$
	3D	0.946 ± 0.033	-0.050 ± 0.119	0.201 ± 0.073
DIS-WS2	FP	$\mathbf{0.285 \pm 0.018}$	$\mathbf{0.203 \pm 0.052}$	$\mathbf{0.470 \pm 0.044}$
	IMG	$\underline{0.300 \pm 0.009}$	$\underline{0.114 \pm 0.098}$	$\underline{0.380 \pm 0.096}$
	SMI	0.321 ± 0.027	-0.014 ± 0.124	0.258 ± 0.135
	Graph	0.309 ± 0.040	0.060 ± 0.183	0.368 ± 0.162
	3D	0.305 ± 0.018	0.087 ± 0.077	0.336 ± 0.099
DIS-RS2	FP	1.737 ± 0.195	-0.053 ± 0.122	$\mathbf{0.299 \pm 0.039}$
	IMG	1.806 ± 0.280	-0.131 ± 0.167	0.113 ± 0.046
	SMI	$\underline{1.727 \pm 0.235}$	$\underline{-0.034 \pm 0.102}$	0.157 ± 0.068
	Graph	$\mathbf{1.721 \pm 0.168}$	$\mathbf{-0.034 \pm 0.057}$	0.100 ± 0.099
	3D	1.873 ± 0.181	-0.238 ± 0.205	$\underline{0.191 \pm 0.085}$

*Bold: best performance. Underline: second-best performance.
FP: ECFP4 fingerprints[9]; IMG: MolScribe[10]; SMI: ChemBERTa[11]; Graph: MGSSL[12]; 3D: ConforFormer[13].

Supplementary Table S15: Performance comparison across single-modalities on INH dataset.

Dataset	Model	RMSE(↓)	R^2(↑)	PCC(↑)
INH-PI	FP	**0.166 ± 0.007**	**0.317 ± 0.058**	**0.585 ± 0.033**
	IMG	0.177 ± 0.005	0.220 ± 0.072	0.486 ± 0.066
	SMI	0.185 ± 0.008	0.148 ± 0.105	0.446 ± 0.074
	Graph	<u>0.173 ± 0.006</u>	<u>0.257 ± 0.104</u>	<u>0.532 ± 0.074</u>
	3D	0.179 ± 0.002	0.200 ± 0.086	0.498 ± 0.057
INH-Ssel	FP	**0.327 ± 0.043**	**0.446 ± 0.069**	**0.691 ± 0.038**
	IMG	0.366 ± 0.038	0.302 ± 0.083	0.577 ± 0.048
	SMI	0.381 ± 0.043	0.240 ± 0.130	0.540 ± 0.076
	Graph	<u>0.340 ± 0.031</u>	<u>0.399 ± 0.052</u>	<u>0.650 ± 0.031</u>
	3D	0.359 ± 0.034	0.323 ± 0.103	0.606 ± 0.050
INH-WS2	FP	**0.214 ± 0.009**	**0.234 ± 0.029**	**0.497 ± 0.025**
	IMG	0.230 ± 0.016	0.114 ± 0.072	0.327 ± 0.116
	SMI	0.236 ± 0.011	0.068 ± 0.037	0.314 ± 0.040
	Graph	<u>0.219 ± 0.012</u>	<u>0.201 ± 0.041</u>	<u>0.459 ± 0.037</u>
	3D	0.227 ± 0.013	0.136 ± 0.045	0.394 ± 0.048
INH-RS2	FP	**1.450 ± 0.045**	**0.353 ± 0.053**	**0.605 ± 0.034**
	IMG	<u>1.615 ± 0.060</u>	0.197 ± 0.076	0.450 ± 0.080
	SMI	1.642 ± 0.034	0.172 ± 0.046	0.457 ± 0.056
	Graph	1.569 ± 0.053	<u>0.245 ± 0.035</u>	<u>0.517 ± 0.029</u>
	3D	1.623 ± 0.066	0.187 ± 0.105	0.503 ± 0.035

*Bold: best performance. Underline: second-best performance.
FP: ECFP4 fingerprints[9]; IMG: MolScribe[10]; SMI: ChemBERTa[11]; Graph: MGSSL[12]; 3D: ConforFormer[13].

Supplementary Table S16: Average rank comparison of selectivity prediction across single modalities.

Dataset	RMSE					R^2					PCC				
	FP	IMG	SMI	Graph	3D	FP	IMG	SMI	Graph	3D	FP	IMG	SMI	Graph	3D
DIS-PI	1	2	3	4	5	1	2	3	4	5	1	2	4	5	3
DIS-Ssel	1	4	2	5	3	1	4	2	5	3	1	4	2	5	3
DIS-WS2	1	2	4	5	3	1	2	4	5	3	1	2	4	5	3
DIS-RS2	3	4	2	1	5	3	4	2	1	5	1	4	3	5	2
INH-PI	1	2	5	4	3	1	2	5	4	3	1	3	4	5	2
INH-Ssel	1	3	5	4	2	1	3	5	4	2	1	3	5	4	2
INH-WS2	1	3	5	4	2	1	3	5	4	2	1	3	5	4	2
INH-RS2	1	2	4	5	3	1	2	4	5	3	1	4	3	5	2
Average	**1.2**	<u>2.8</u>	3.8	4	3.2	**1.2**	<u>2.8</u>	3.8	4	3.2	**1**	3.1	3.8	4.8	<u>2.4</u>

*Bold: best performance. Underline: second-best performance.
FP: ECFP4 fingerprints[9]; IMG: MolScribe[10]; SMI: ChemBERTa[11]; Graph: MGSSL[12]; 3D: ConforFormer[13].

References

[1] Swanson, K.; Walther, P.; Leitz, J.; Mukherjee, S.; Wu, J. C.; Shivnaraine, R. V.; Zou, J. ADMET-AI: a machine learning ADMET platform for evaluation of large-scale chemical libraries. *Bioinformatics* **2024**, *40*, btae416.

[2] Lu, X.; Xie, L.; Xu, L.; Mao, R.; Xu, X.; Chang, S. Multimodal fused deep learning for drug property prediction: Integrating chemical language and molecular graph. *Computational and Structural Biotechnology Journal* **2024**, *23*, 1666–1679.

[3] Öztürk, H.; Özgür, A.; Ozkirimli, E. DeepDTA: deep drug–target binding affinity prediction. *Bioinformatics* **2018**, *34*, i821–i829.

[4] Nguyen, T.; Le, H.; Quinn, T. P.; Nguyen, T.; Le, T. D.; Venkatesh, S. GraphDTA: predicting drug–target binding affinity with graph neural networks. *Bioinformatics* **2021**, *37*, 1140–1147.

[5] Li, Z.; Ren, P.; Yang, H.; Zheng, J.; Bai, F. TEFDTA: a transformer encoder and fingerprint representation combined prediction method for bonded and non-bonded drug–target affinities. *Bioinformatics* **2024**, *40*, btad778.

[6] Bidgoli, A. H.; Mahdavi, M.; Malek, H. Structure-free drug–target affinity prediction using protein and molecule language models. *Journal of Cheminformatics* **2026**,

[7] Hunter, F. M.; Ioannidis, H.; Bento, A. P.; Bosc, N.; Corbett, S.; Felix, E.; Magarinos, M. P.; Manners, E.; Smit, I. A.; de Veij, M.; others Drug and Clinical Candidate Drug Data in ChEMBL. *Journal of Medicinal Chemistry* **2025**, *68*, 19800–19827.

[8] Kooistra, A. J.; Kanev, G. K.; van Linden, O. P.; Leurs, R.; de Esch, I. J.; de Graaf, C. KLIFS: a structural kinase-ligand interaction database. *Nucleic acids research* **2016**, *44*, D365–D371.

[9] Rogers, D.; Hahn, M. Extended-connectivity fingerprints. *Journal of chemical information and modeling* **2010**, *50*, 742–754.

[10] Qian, Y.; Guo, J.; Tu, Z.; Li, Z.; Coley, C. W.; Barzilay, R. MolScribe: robust molecular structure recognition with image-to-graph generation. *Journal of Chemical Information and Modeling* **2023**, *63*, 1925–1934.

[11] Chithrananda, S.; Grand, G.; Ramsundar, B. ChemBERTa: large-scale self-supervised pretraining for molecular property prediction. *arXiv preprint arXiv:2010.09885* **2020**,

[12] Zhang, Z.; Liu, Q.; Wang, H.; Lu, C.; Lee, C.-K. Motif-based graph self-supervised learning for molecular property prediction. *Advances in Neural Information Processing Systems* **2021**, *34*, 15870–15882.

[13] Klein, M. P.; Rudenko, I.; Pidko, E. A.; Bushmarinov, I. ConforFormer: representation for molecules through understanding of conformers. *Digital Discovery* **2026**, *5*, 2639–2649.
